# Longitudinal evidence for a mutually reinforcing relationship between white matter hyperintensities and cortical thickness in cognitively unimpaired older adults

**DOI:** 10.1186/s13195-024-01606-5

**Published:** 2024-10-28

**Authors:** Jose Bernal, Inga Menze, Renat Yakupov, Oliver Peters, Julian Hellmann-Regen, Silka Dawn Freiesleben, Josef Priller, Eike Jakob Spruth, Slawek Altenstein, Anja Schneider, Klaus Fliessbach, Jens Wiltfang, Björn H. Schott, Frank Jessen, Ayda Rostamzadeh, Wenzel Glanz, Enise I. Incesoy, Katharina Buerger, Daniel Janowitz, Michael Ewers, Robert Perneczky, Boris-Stephan Rauchmann, Stefan Teipel, Ingo Kilimann, Christoph Laske, Sebastian Sodenkamp, Annika Spottke, Anna Esser, Falk Lüsebrink, Peter Dechent, Stefan Hetzer, Klaus Scheffler, Stefanie Schreiber, Emrah Düzel, Gabriel Ziegler

**Affiliations:** 1 Institute of Cognitive Neurology and Dementia Research, Otto-Von-Guericke University Magdeburg, Magdeburg, Germany; 2 German Centre for Neurodegenerative Diseases (DZNE), Magdeburg, Germany; 3 Centre for Clinical Brain Sciences, the University of Edinburgh, Edinburgh, UK; 4 UK Dementia Research Institute Centre at the University of Edinburgh, Edinburgh, UK; 5 German Centre for Neurodegenerative Diseases (DZNE), Berlin, Germany; 6 Charité – Universitätsmedizin Berlin, Institute of Psychiatry and Psychotherapy, Berlin, Germany; 7 Charité – Universitätsmedizin Berlin, Department of Psychiatry and Neurosciences, Campus Benjamin Franklin, Berlin, Germany; 8 German Centre for Mental Health (DZPG), Berlin, Germany; 9 Department of Psychiatry and Psychotherapy, Charité, Berlin, Germany; 10 School of Medicine, Department of Psychiatry and Psychotherapy, Technical University of Munich, Munich, Germany; 11 German Centre for Neurodegenerative Diseases (DZNE), Bonn, Germany; 12 Department of Old Age Psychiatry and Cognitive Disorders, University Hospital Bonn and University of Bonn, Bonn, Germany; 13 German Centre for Neurodegenerative Diseases (DZNE), Göttingen, Germany; 14 Department of Psychiatry and Psychotherapy, University Medical Centre Göttingen, University of Göttingen, Göttingen, Germany; 15 Neurosciences and Signalling Group, Institute of Biomedicine (iBiMED), Department of Medical Sciences, University of Aveiro, Aveiro, Portugal; 16 Leibniz Institute for Neurobiology, Brenneckestr. 6, 39118 Magdeburg, Germany; 17 Department of Psychiatry, Medical Faculty, University of Cologne, Cologne, Germany; 18 Excellence Cluster On Cellular Stress Responses in Aging-Associated Diseases (CECAD), University of Cologne, Cologne, Germany; 19 Department for Psychiatry and Psychotherapy, University Clinic Magdeburg, Magdeburg, Germany; 20 German Centre for Neurodegenerative Diseases (DZNE), Munich, Germany; 21 Institute for Stroke and Dementia Research (ISD), University Hospital, LMU Munich, Munich, Germany; 22 Department of Psychiatry and Psychotherapy, University Hospital, LMU Munich, Munich, Germany; 23 Munich Cluster for Systems Neurology (SyNergy) Munich, Munich, Germany; 24 Ageing Epidemiology Research Unit (AGE), School of Public Health, Imperial College London, London, UK; 25 Sheffield Institute for Translational Neuroscience (SITraN), University of Sheffield, Sheffield, UK; 26 Department of Neuroradiology, University Hospital LMU, Munich, Germany; 27 German Centre for Neurodegenerative Diseases (DZNE), Rostock, Germany; 28 Department of Psychosomatic Medicine, Rostock University Medical Centre, Rostock, Germany; 29 German Centre for Neurodegenerative Diseases (DZNE), Tübingen, Germany; 30 Section for Dementia Research, Hertie Institute for Clinical Brain Research and Department of Psychiatry and Psychotherapy, University of Tübingen, Tübingen, Germany; 31 Department of Psychiatry and Psychotherapy, University of Tübingen, Tübingen, Germany; 32 Department of Neurology, University of Bonn, Bonn, Germany; 33 Department of Cognitive Neurology, MR-Research in Neurosciences, Georg-August-University, Göttingen, Germany; 34 Berlin Centre for Advanced Neuroimaging, Charité – Universitätsmedizin Berlin, Berlin, Germany; 35 Department for Biomedical Magnetic Resonance, University of Tübingen, Tübingen, Germany; 36 Department of Neurology, University Hospital Magdeburg, Magdeburg, Germany

**Keywords:** White Matter Hyperintensities, Cortical Thickness, Latent Growth Curve Model, Longitudinal Modelling, Structural Magnetic Resonance Imaging

## Abstract

**Background:**

For over three decades, the concomitance of cortical neurodegeneration and white matter hyperintensities (WMH) has sparked discussions about their coupled temporal dynamics. Longitudinal studies supporting this hypothesis nonetheless remain scarce.

**Methods:**

We applied global and regional bivariate latent growth curve modelling to determine the extent to which WMH and cortical thickness were interrelated over a four-year period. For this purpose, we leveraged longitudinal MRI data from 451 cognitively unimpaired participants (DELCODE; median age 69.71 [IQR 65.51, 75.50] years; 52.32% female). Participants underwent MRI sessions annually over a four-year period (1815 sessions in total, with roughly four MRI sessions per participant). We adjusted all models for demographics and cardiovascular risk.

**Results:**

Our findings were three-fold. First, larger WMH volumes were linked to lower cortical thickness (*σ* = -0.165, *SE* = 0.047, *Z* = -3.515, *P* < 0.001). Second, individuals with higher WMH volumes experienced more rapid cortical thinning (*σ* = -0.226, *SE* = 0.093, *Z* = -2.443, *P* = 0.007), particularly in temporal, cingulate, and insular regions. Similarly, those with lower initial cortical thickness had faster WMH progression (*σ* = -0.141, *SE* = 0.060, *Z* = -2.336, *P* = 0.009), with this effect being most pronounced in temporal, cingulate, and insular cortices. Third, faster WMH progression was associated with accelerated cortical thinning (*σ* = -0.239, *SE* = 0.139, *Z* = -1.710, *P* = 0.044), particularly in frontal, occipital, and insular cortical regions.

**Conclusions:**

Our study suggests that cortical thinning and WMH progression could be mutually reinforcing rather than parallel, unrelated processes, which become entangled before cognitive deficits are detectable.

**Trial registration:**

German Clinical Trials Register (DRKS00007966, 04/05/2015).

**Supplementary Information:**

The online version contains supplementary material available at 10.1186/s13195-024-01606-5.

## Introduction

Cortical thinning and white matter hyperintensities (WMH) progression are well-known ageing processes that take place throughout middle and late adulthood [1–9]. Both processes appear to be influenced by genetic and lifestyle factors [2, 10–15] as well as by the onset and progression of neurodegenerative and cerebrovascular diseases [1, 2, 9, 16–20]. Although overlapping risk factors may offer an initial explanation for their concomitance [3, 6, 11, 21, 22], their persistent association after controlling for demographics and traditional cardiovascular risk factors [3, 6, 10, 23–25] has sparked more than three decades of research into coupled temporal dynamics [3, 26].

Coupled temporal dynamics between WMH and cortical atrophy are currently discussed from two non-exclusive perspectives: the cerebrovascular and the neurodegenerative hypotheses [17, 26]. The cerebrovascular hypothesis posits that ischaemic and hypoxic damages—operationalised as WMH [15, 27–29]—may initially result in the depletion of oxygen, nutrients, and trophic support in perilesional regions [16, 28]. Subsequently, these damages may also disrupt the function and metabolic demands of compromised white matter tracts and associated cortical regions, leading to cortical atrophy [6, 9, 17, 27, 30]. On the other hand, the neurodegenerative hypothesis proposes that cortical neurodegeneration could contribute to WMH formation [17, 26, 29, 31–34], especially in conjunction with tau pathologies [26, 29, 34]. Excessive tau phosphorylation could promote microtubule destabilisation, thereby causing axonal transport dysfunction, energy depletion, and calcium imbalance—a hallmark of Wallerian degeneration [34]. In the light of the posterior dominance of WMH in Alzheimer’s disease (AD) [26, 35–38], both hypotheses would require effects of cortical neurodegeneration and WMH to be particularly pronounced in parietooccipital brain regions. Longitudinal evidence and multivariate modelling substantiating these two hypotheses remain nonetheless scarce, especially in cognitively unimpaired older adults [1].

Here, we leveraged bivariate latent growth curve modelling (BLGCM) to examine the bidirectional relationship between WMH and regional cortical thickness over four years in older individuals without objective cognitive impairment. We specifically sought to answer four main research questions:

- Q1. Upon study entry, do individuals with larger total WMH volumes have lower cortical thickness? (*intercept-intercept covariance)*
- Q2. Do individuals with larger total WMH volumes at study entry experience faster cortical thinning? (cerebrovascular hypothesis; *intercept-slope covariance)*
- Q3. Do individuals with thinner cortices at study entry exhibit a faster increase in total WMH volumes? (neurodegenerative hypothesis; *intercept-slope covariance)*
- Q4. Do individuals exhibiting faster total WMH volume increases also undergo faster cortical thinning over time? (*slope-slope covariance)*

## Methods

### Study participants

We used baseline and annual follow-up data for up to 48 months from participants of the observational longitudinal multicentre DELCODE (DZNE Longitudinal Cognitive Impairment and Dementia) Study [39]. DELCODE is a memory-clinic-based observational multicentre study from the German Centre for Neurodegenerative Diseases (DZNE) that uses multimodal assessment of preclinical, prodromal, and clinical stages of AD, with a particular focus on subjective cognitive decline. Study participants were either referred to the university-affiliated memory centres, including self-referrals, or were recruited through standardised public advertisements [39]. In this paper, we focused on cognitively unimpaired participants who underwent at least three MRI scanning sessions and whose follow-up MRI sessions took place within four months prior or after their yearly comprehensive examination. We followed the recommendation of conducting at least three assessments per subject to reliably estimate linear trends [40].

During the baseline visit, participants underwent a thorough evaluation at their local study site, which included medical history checks, a psychiatric and neurological examination, neuropsychological testing, blood and cerebrospinal fluid (CSF) collection, and MRI in accordance with local standards. All DELCODE sites used the Consortium to Establish a Registry for AD (CERAD-plus) neuropsychological test battery to assess cognitive function. Cognitively unimpaired participants performed better than -1.5 standard deviations of the age-, sex-, and education-adjusted normal performance on all subtests of the test battery [39].

The primary inclusion criteria for all groups were being aged 60 or older, fluency in German, the ability to provide informed consent, and having a study partner available [39]. The main exclusion criteria for all groups were conditions that clearly interfered with participation in the study or its procedures, including significant sensory impairment. The following medical conditions were considered exclusion criteria: current or history of major depressive episode and major psychiatric disorders either at baseline (e.g., psychotic disorder, bipolar disorder, substance abuse), neurodegenerative diseases other than AD, vascular dementia, history of stroke with residual clinical symptoms, history of disseminated malignant disease, severe or unstable medical conditions, and clinically significant vitamin B12 deficiency at baseline. Prohibited drugs included chronic use of psychoactive compounds with sedative or anticholinergic effects, use of anti-dementia agents, and investigational drugs for the treatment of dementia or cognitive impairment one month before study entry and throughout the duration of the study [39].

All participants provided their written informed consent in accordance with the Declaration of Helsinki at baseline. DELCODE has been registered within the German Clinical Trials Register (DRKS00007966, 04/05/2015). Ethics committees of the medical faculties of all participating sites (i.e., Berlin (Charité—Universitätsmedizin Berlin), Bonn, Cologne, Göttingen, Magdeburg, Munich (Ludwig-Maximilians-University), Rostock, and Tübingen) approved the DELCODE study protocol before inclusion of the first participants. The ethics committee of the medical faculty of the University of Bonn led and coordinated the process [39].

### Total cardiovascular risk score

We established a total cardiovascular risk score for each participant by tallying their dichotomised (y/n) history of smoking, presence of obesity, hyperlipidemia, arterial hypertension, and diabetes, as reported in their medical records. We corrected the sum of present risk factors by the amount of available information. For example, if an individual had a history of arterial hypertension and diabetes but we did not have data on smoking, obesity, or hyperlipidemia, the final score would be 1.00. The corrected total cardiovascular risk scores ranged from 0.00 to 1.00, where the lowest and highest values denoted the absence or presence of all available risk factors, respectively.

### MRI

MRI data were acquired at nine DZNE sites or associated university medical centres equipped with 3 T Siemens MR scanners. In the present study, we leveraged the following structural sequences: T1w MPRAGE (full head coverage, 3D acquisition, GRAPPA factor 2, 1 mm^3^ isotropic, 256 × 256 px, 192 sagittal slices, TR/TE/TI 2500/4.33/1100 ms, FA 7°) and T2w FLAIR (full head coverage, 3D acquisition, 1 mm^3^ isotropic, 256 × 256 px, 192 sagittal slices, TR/TE/TI 5000/394/1800 ms). The DZNE imaging network oversaw operating procedures, as well as quality assurance and assessment (iNET, Magdeburg) [39].

### MRI-based measurements

#### Cortical thickness

We used the CAT12 longitudinal pipeline [41] (neuro-jena.github.io) to reconstruct cortical thickness surfaces for each subject and for each time point (ageing workflow; default parameters, except for final resolution, which we set to 1 mm^3^). We then estimated mean thickness throughout the whole brain cortex, cerebral lobes, and cingulate and insular cortices.

### WMH segmentation

We segmented WMH using the AI-augmented version of the Lesion Segmentation Toolbox (LST-AI) [42–44] and based the segmentation on both T1w MPRAGE and T2w FLAIR imaging data. We then calculated total WMH volumes.

### Statistical analyses

We conducted all data analyses in RStudio (v1.3.1073; R v4.0.2) using lavaan (v0.6–16). We created figures using ggplot2 (v3.4.3) and the ENIGMA toolbox [45].

We applied LGCM to determine the extent to which WMH and cortical thickness were interrelated over time. (B)LGCMs [46] are a powerful class of structural equation models (SEM) to describe sample average trajectories of one or two constructs over time through the specification of latent intercepts and latent slopes (i.e., initial levels and rates of change). The primary advantage of BLGCM over linear mixed-effect (LME) models is its ability to simultaneously and symmetrically model changes in two outcome variables. BLGCM allows for the simultaneous estimation of growth trajectories for two latent constructs, facilitating the examination of their interrelationships over time [46–49]. In contrast, LME modelling deals with a single construct at a time, requiring separate models for each and post-hoc analyses to establish the association between individual intercepts and slopes.

**Fig. 1:**
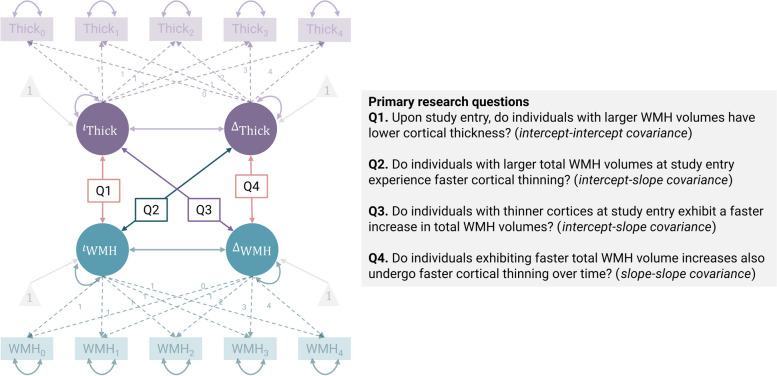
BLGCM to probe the coupling of cortical thickness and WMH over repeated measures. Illustration of the longitudinal structural equation modelling (SEM) model. We employed the conventional notation with squared variables indicating observed and measured variables (manifest variables) and circular ones referring to latent (unobserved) variables. Single-headed solid arrows illustrate a modelled relationship between two variables, with the arrow pointing towards the dependent variable. Single-headed dashed arrows signify a relationship between two variables, where the weight is fixed. Double-headed arrows represent the covariance (hyperparameter) between two variables. Grey triangles represent latent intercept estimates. We further adjusted latent intercepts and slopes for age, sex, years of education, total cardiovascular risk factors, and TICV. We omitted these paths for visualisation purposes

We carried out univariate and bivariate LGCMs. We first used univariate LGCMs for contextualisation purposes, to examine which covariates were associated with the baseline measurements and potential changes over repeated measures. We then focused on BLGCMs to *assessed interrelationships between WMH and cortical thickness* over time, by assessing the covariance between these four latent growth parameters [49] (Fig. 1).

We conducted global and regional analyses to identify associations at two levels of granularity. In the global analysis—with no spatial specificity—we focused on the interrelationship between mean cortical thickness and total WMH volume. In order to elucidate potential region-specific and cross-domain relationships, we additionally examined the relationship between total WMH volume and regional cortical thicknesses. Note that our approach is similar to a mass-univariate analysis scheme, with the difference being that we investigated region-specific effects through LGCM rather than through GLM. To reduce the dimensionality and thereby improve the feasibility of our multivariate SEM analysis, we considered (corresponding) bilateral regions jointly. We present the completely standardised solutions and include both standardised and unstandardised solutions in the supplementary material (see Additional File 2).

#### Adjusting for covariates and confounders

We adjusted latent intercepts and slopes for effects of age, sex, years of education, total cardiovascular risk factor score, and total intracranial volume (TICV) in all models.

#### Data transformation

We applied a Box-Cox transformation to WMH volumes and exponential transformation cortical thickness to address skewness [50]. We z-scored all variables (pooled across timepoints) prior to model fitting. For the purpose of contextualisation and plotting, we back-transformed the fitted growth curve parameters afterwards.

#### Model fitting

We employed the maximum likelihood robust estimator to fit the model. We used the full information maximum likelihood estimation to handle missing values. To check for compliance with the assumption of missingness at random, we tested whether missingness in one column (1: missing; 0: not missing) could be predicted from the remaining ones. In all instances, the resulting p-values exceeded 0.05.

Prior to model fitting and solely to ensure model fit, we used Tukey’s fences to identify and remove outliers in all data points (threshold of 1.5) [51]. The number of individual data points that were removed can be retrieved from Supplementary Table 1 in Additional File 1. We evaluated the fit of global and regional models by analysing their root mean square error of approximation (RMSEA; values ≤ 0.05 indicate good fit), comparative fit index (CFI; values exceeding 0.95 indicate good fit), and standardised root mean residual (SRMR; values < 0.08 suggest good fit) [52]. For the sake of transparency, when discussing the models, we disclosed their convergence and compliance with the aforementioned thresholds.

#### Correction for multiple comparisons

We employed the False Discovery Rate (FDR) correction [53] method to account for the issue of multiple comparisons on all region-wise analyses.

## Results

### Study participants

Among the 722 cognitively unimpaired participants enrolled in the DELCODE study, 451 attended a minimum of three annual visits (1815 MRI sessions; median age 69.71 [IQR 65.51, 74.50] years; 52.32% females; median years of education 14 [IQR 13, 17]). The average number of scans per participant was approximately four (4.192 [95%-CI 4.118, 4.264]), with 191, 155, and 105 participants attending exactly five, four, and three of the five annual visits, respectively. Aside from obesity, which had missing records for seven cognitively unimpaired participants, we had complete information for all other cardiovascular risk variables included in the total cardiovascular risk score.

### Univariate findings

#### WMH volumes

##### Model fit

The univariate LGCM on WMH volumes converged and provided good model fit (*RMSEA* = 0.000, *CFI* = 1.000, *SRMR* = 0.009).

##### Do covariates explain the variability in WMH volumes?

WMH volumes were larger in older individuals (*β*_*Age*_ = 0.374, *SE* = 0.042, *Z* = 8.932, *P* < 0.001) and in those with higher total cardiovascular risk factor scores (*β*_*Vascular Risk*_ = 0.102, *SE* = 0.044, *Z* = 2.303, *P* = 0.021). Females had larger WMH volumes than males (*β*_*Female*_ = 0.189, *SE* = 0.060, *Z* = 3.161, *P* = 0.002), despite females in our sample being on average younger (covariance between female sex and age = -0.165, *SE* = 0.046, *Z* = -3.607, *P* < 0.001) and having lower total cardiovascular risk scores than males (covariance between female sex and cardiovascular risk = -0.184, *SE* = 0.045, *Z* = -4.128, *P* < 0.001). In addition, females had on average fewer years of education than males (covariance between female sex and years of education = -0.238, *SE* = 0.043, *Z* = -5.569, *P* < 0.001).

##### Do WMH volumes change over time, and which covariates relate to change rates?

**Fig. 2:**
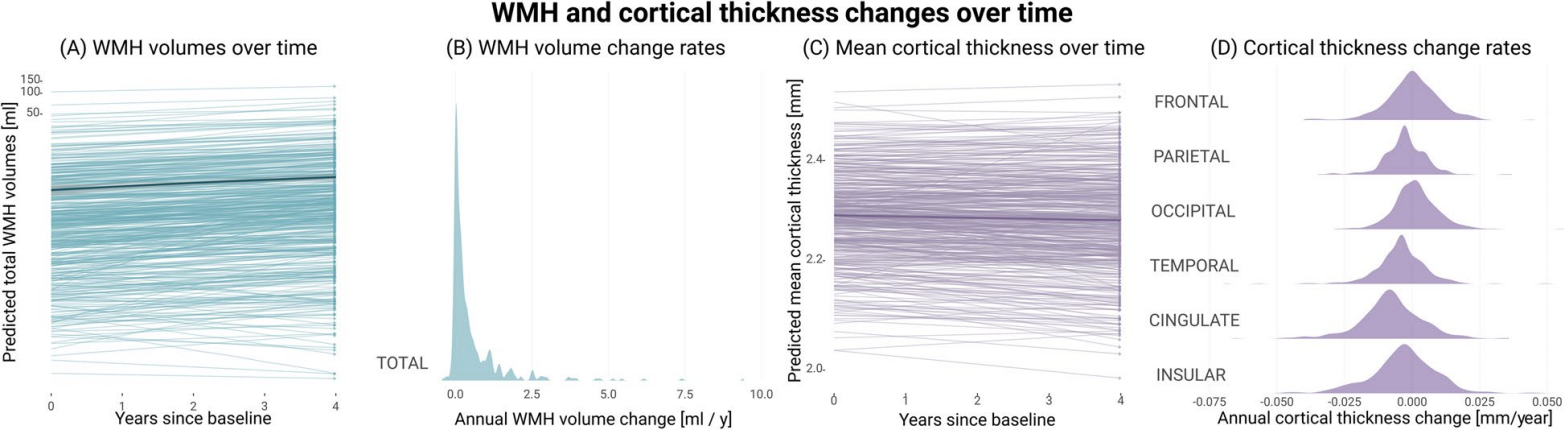
Changes in WMH volumes and cortical thickness over four years. We obtained latent intercepts and slopes for each individual through the application of univariate LGCM to WMH volumes and cortical thickness (separate models for each neuroimaging feature). We used them to compute latent growth curve parameters and predict individual trajectories, corrected for age, sex, years of education, total cardiovascular risk scores, and TICV. Prior to plotting and to enhance interpretability, we back-transformed all predicted measurements. **A** Total WMH volume trajectories, as predicted by the model. Light blue lines represent the predicted trajectories and the dark blue one the average one. **B** Back-transformed individual factor scores of latent slopes for WMH, summarised in the density plots, indicate that WMH volumes generally increased over time. We adjusted density plots such that the modes attain the highest value, irrespective of the actual frequency. The rate of change varied substantially across individuals in both cases. **C** Mean cortical thickness trajectories, as predicted by the model. Light purple lines represent the predicted trajectories and the dark purple one the average one. **D** Back-transformed individual factor scores of cortical thicknesses across the considered brain regions. The variability in change rates indicated significant inter-individual differences in regional cortical thinning

WMH volumes generally increased over the follow-up period of four years (Fig. 2A; intercept of WMH slope = 1.117, *SE* = 0.110, *Z* = 10.177, *P* < 0.001). On average, individuals experienced an increase in WMH volumes of about 0.536 [95%-CI 0.442, 0.630] ml/year. WMH progression rates varied substantially among individuals (Fig. 2B; variance of WMH slope = 0.987, *SE* = 0.015, *Z* = 67.281, *P* < 0.001) and, even though most individuals experienced consistent increases in WMH volumes over time, a few (10%) showed decreases during the same period. The most evident case of WMH volume regression was observed in a female participant in her 60 s, with a total cardiovascular risk score of 0.0, and 15 years of education (higher education). Regression in this participant was most noticeable in occipital brain regions and could be attributed to a loss of periventricular tissue caused by a substantial enlargement of the occipital horns of the lateral ventricles over time (Supplementary Figure S1 in Additional File 1).

## Cortical thickness

### Model fit

All univariate LGCM fitted to cortical thickness converged and had good fit indices (*RMSEA* ≤ 0.05, *CFI* ≥ 0.95, *SRMR* ≤ 0.05).

### Do covariates explain the variability in cortical thickness?

Mean cortical thickness values were generally lower in older individuals (*β*_*Age*_ = -0.334, *SE* = 0.048, *Z* = -6.964, *P* < 0.001). We did not find sex, years of education, or cardiovascular risk factors to relate to baseline cortical measurements.

### Does cortical thickness change over time, and which covariates relate to change rates?

The thickness of cerebral cortex generally decreased over the course of four years at an average rate of approximately -0.002 [95%-CI -0.003, -0.001] mm/year (Fig. 2C; intercept of mean cortical thickness slope = -0.206, *SE* = 0.096, *Z* = -2.152, *P* = 0.031). On average, this reduction was distributed across the cingulate, temporal, and parietal cortices, with average annual thinning rates of 0.008 [95%-CI 0.007, 0.009] mm/year, 0.003 [95%-CI 0.002, 0.004] mm/year, and 0.003 [95%-CI 0.002, 0.003] mm/year, respectively (Fig. 2D). Frontal, occipital, and insular cortices showed, on average, no significant changes over the four-year period (${P}_{FDR}>0.05$).

Cortical thinning rates varied substantially across individuals (variance of mean cortical thickness slope = 0.902, *SE* = 0.051, *Z* = 17.837, *P* < 0.000). The age of the patient accounted for part of this inter-individual variability, with annual cortical thinning rates generally slowing with advancing age (*β*_*Age*_ = -0.275, *SE* = 0.082, *Z* = -3.338, *P* = 0.001). Other covariates, including sex, years of education, and cardiovascular risk score, did not show a clear relationship with cortical thinning.

#### Bivariate findings

##### Model fit

All BLGCMs also converged and had a satisfactory model fit (*RMSEA* ≤ 0.05, *CFI* ≥ 0.95, *SRMR* ≤ 0.05).

##### Q1. Upon study entry, do individuals with larger total WMH volumes have lower cortical thickness?

**Fig. 3:**
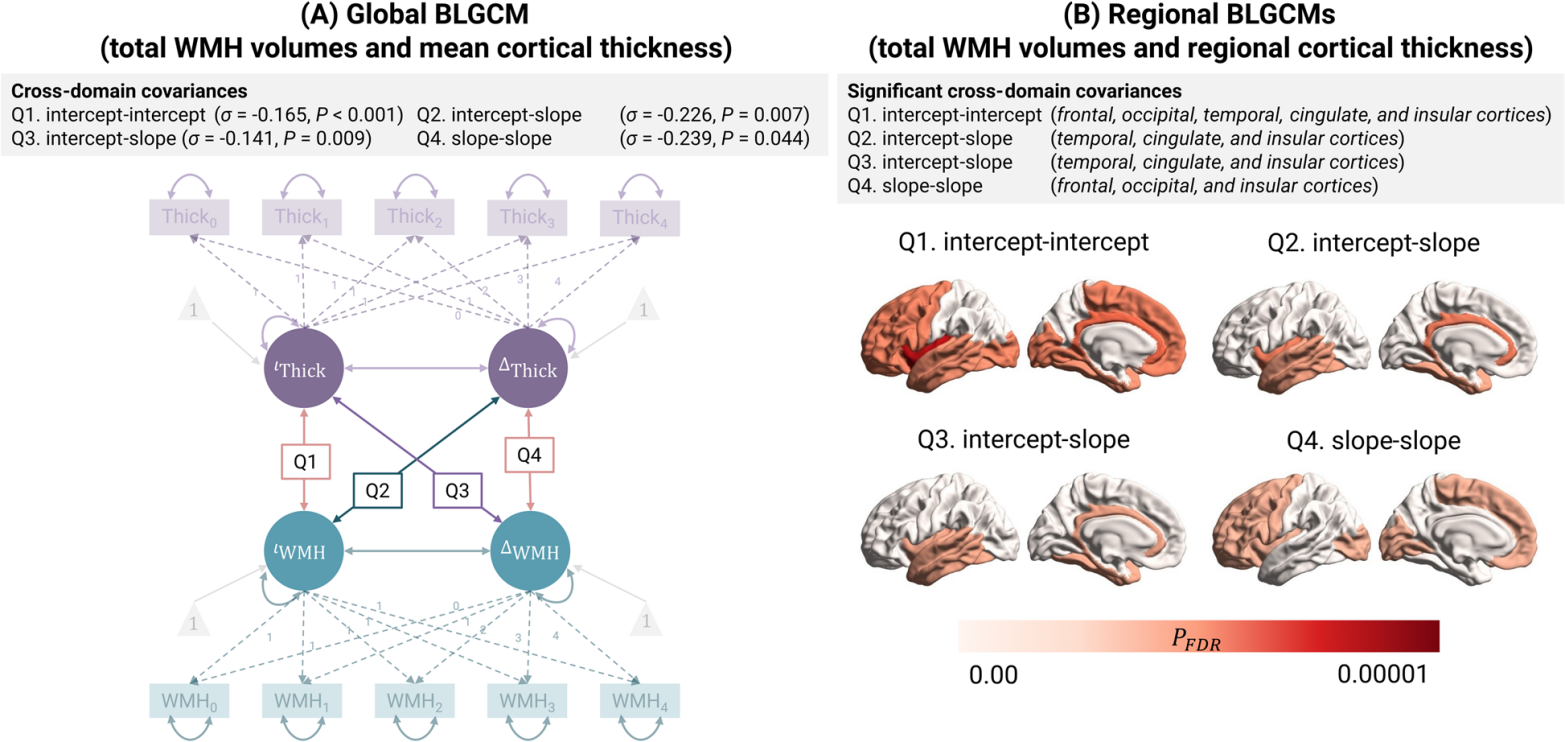
Relationship between latent growth parameters from global and regional BLGCMs. We employed longitudinal BLGCMs to characterise the spatiotemporal interrelation between WMH volumes and cortical thickness over the span of four years. We adjusted latent intercepts and slopes for age, sex, years of education, total cardiovascular risk scores, and TICV. (**A**) Relationship between latent growth curve parameters obtained from the global model. At baseline, individuals with larger WMH volumes had lower cortical thickness. Over time, those experiencing rapid cortical thinning initially had large total WMH volumes. Similarly, those with rapid WMH progression had thinner cortices at baseline. In general, faster WMH progression was linked to more rapid cortical thinning (Q4). (**B**) Regional analyses suggest cross-domain associations have regional specificities. We applied FDR correction to account for multiple comparisons. In regions highlighted in red, we found a statistically significant covariance between latent growth curve parameters after FDR correction (${P}_{FDR}<0.05$)

At baseline, individuals with larger total WMH volumes had lower mean cortical thickness values (Fig. 3A Q1; global model, *σ* = -0.165, *SE* = 0.047, *Z* = -3.515, *P* < 0.001). With the exception of the parietal cortex, this association was generally present across cortical regions (Fig. 3B Q1; regional model).

##### Q2. Do individuals with larger total WMH volumes at study entry experience faster cortical thinning?

**Fig. 4:**
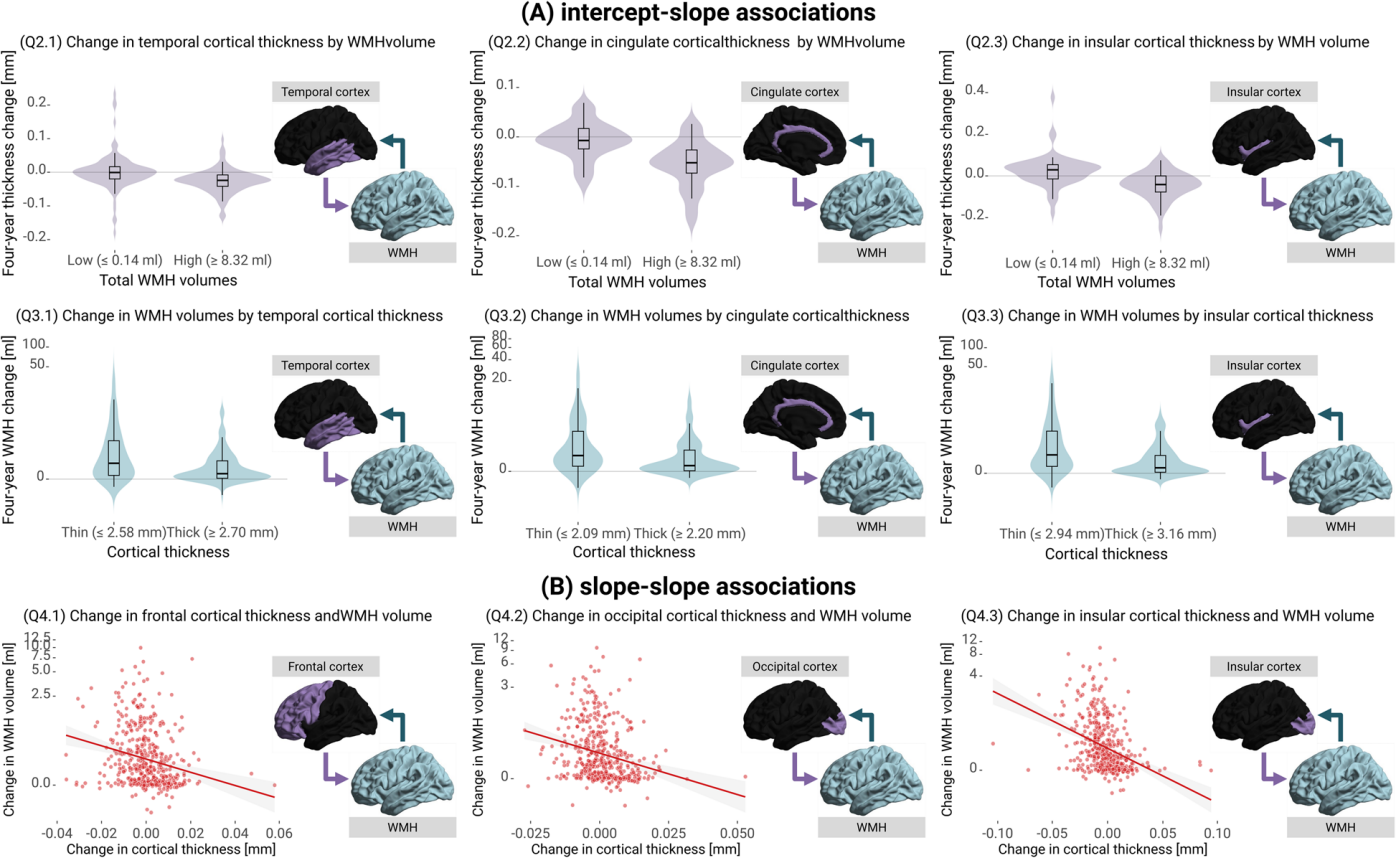
Cross-domain intercept-slope and slope-slope associations.**A** Predicted four-year changes in cortical thickness and WMH trajectories, stratified by baseline WMH volumes and cortical thickness, respectively. We categorised individuals based on whether their latent intercepts were below the 25th or above the 75th percentile, respectively. **B** Relationship between predicted changes in cortical thickness and WMH volumes over four years. We back-transformed all predicted measurements to plotting for interpretability purposes. We adjusted latent intercepts and slopes for age, sex, years of education, total cardiovascular risk scores, and TICV

Individuals with larger baseline WMH volumes had faster thinning of the cerebral cortex (Fig. 3A Q2; global model, *σ* = -0.226, *SE* = 0.093, *Z* = -2.443, *P* = 0.007), especially across temporal, cingulate, and insular cortices (Fig. 3B Q2; regional model, temporal σ = -0.180, *SE* = 0.082, *Z* = -2.197, *P*_*FDR*_ = 0.028; cingulate σ = -0.217, *SE* = 0.074, *Z* = -2.953, *P*_*FDR*_ = 0.008; insular σ = -0.280, *SE* = 0.101, *Z* = -2.773, *P*_*FDR*_ = 0.008). The relative loss in cortical thickness in the temporal, cingulate, and insular cortices was, on average, 1.46% higher in individuals with the highest 25% of WMH volumes compared to those in the lowest 25% (Fig. 4A Q2.1-Q2.3).

##### Q3. Do individuals with thinner cortices at study entry exhibit a faster increase in total WMH volumes?

Individuals who experienced faster progression of WMH had lower mean cortical thickness values at baseline (Fig. 3A Q3; global model, *σ* = -0.141, *SE* = 0.060, *Z* = -2.336, *P* = 0.009). Closer examination of this relationship revealed that it was particularly evident in those with thin temporal, cingulate, and insular cortices (Fig. 3B Q3; regional model, temporal* σ* = -0.135, *SE* = 0.064, *Z* = -2.122, *P*_*FDR*_ = 0.034; cingulate *σ* = -0.148, *SE* = 0.063, *Z* = -2.366, *P*_*FDR*_ = 0.034; insular *σ* = -0.154, *SE* = 0.070, *Z* = -2.202, *P*_*FDR*_ = 0.034). To put into perspective, the relative increase in WMH volumes over a four-year period was, on average, at least 11.01% higher in individuals with the thinnest temporal, cingulate, or insular cortices compared to those with the thickest cortices (thinnest 25% vs thickest 25%) (Fig. 4A Q3.1-Q3.3).

##### Q4. Do individuals exhibiting faster total WMH volume increases also undergo faster cortical thinning over time?

Over time, individuals who underwent faster WMH progression simultaneously experienced faster cortical thinning (Fig. 3A Q4; global model,* σ* = -0.239, *SE* = 0.139, *Z* = -1.710, *P* = 0.044). This association was evident in frontal, occipital and insular regions (Fig. 3B Q4, Fig. 4B; global model, frontal *σ* = -0.261, *SE* = 0.132, *Z* = -1.982, *P*_*FDR*_ = 0.047, occipital *σ* = -0.315, *SE* = 0.140, *Z* = -2.255, *P*_*FDR*_ = 0.047, insular *σ* = -0.274, *SE* = 0.131, *Z* = -2.097, *P*_*FDR*_ = 0.047). Other cortical regions did not show significant evidence of this association.

## Discussion

We studied the interrelationships between WMH and cortical thickness over a four-year period in 451 older adults without objective cognitive impairment (1815 MRI sessions in total) using a longitudinal modelling approach. We made both methodological and clinical contributions to the ongoing efforts to understand the relationship between cerebrovascular dysfunction and neurodegeneration. First, our study demonstrates the potential of integrating surface-based morphometry and BLGCM to investigate interrelationships between neuroimaging markers over time. Second, our findings support the notion that cortical thinning and WMH progression might be mutually reinforcing processes, entangled over a four-year period in a complex and region-specific manner. Our results suggest that this coupling takes place even among individuals with a low vascular risk, given DELCODE's inclusion and exclusion criteria.

### WMH progression

WMH generally progressed over the course of four years, reiterating that ageing is associated with WMH increase and constitutes a major risk factor for white matter pathology [2, 14, 15, 28, 54]. Significant individual differences in WMH volume changes suggest, however, that there are numerous other factors that were not accounted for in our study that might contribute to subject-specific progression of WMH in ageing. For example, heterogeneity of WMH volumes and progression rates could be reflective of the brain’s ability to respond to and heal from white matter injuries. By extension, heterogeneity of WMH volumes and progression rates could be reflective of past and current socioeconomic status and cardiovascular risk factors, as well as the adoption of an unhealthy lifestyle [2, 55, 56]. This might explain why greater cardiovascular risk scores was associated with higher baseline WMH volumes in our sample.

Interestingly, even though, in our study sample, males were generally older than females and had higher cardiovascular risk factor scores than females, females showed significantly larger WMH volumes at baseline compared to males even after accounting for TICV. WMH progression rates over the course of four years between sexes were nonetheless comparable. For these two scenarios to be compatible, WMH would clearly need to evolve faster in females than in males before the age of 70 years (i.e., the median age in this study). Menopause may constitute a potential explanation for this sex-specific susceptibility to WMH. A relatively recent work in the Rhineland study, a large population-based German cohort, found that while pre-menopausal women and men of similar age did not differ in WMH volumes, post-menopausal women did have significantly larger WMH volumes compared to men of similar age [57]. This finding suggests that indeed menopause and accompanying hormonal and physiological changes might be behind this sex-difference [57]. Another explanation could be that elderly women in this ageing cohort had, on average, lower educational attainment, which could also contribute to their vulnerability to CSVD. The likely multifactorial nature of this finding requires careful consideration during modelling and reporting as well as dedicated analysis shedding light on the mechanisms potentially mediating such a vulnerability.

Albeit less commonly, a small number of participants exhibited clear and consistent WMH volume regression throughout the study period, as reported in previous literature [14, 58]. The case with the most regression coincided with the progression of ventricular enlargement. While frequently discussed in the context of a radiological or technical issue [58], our finding suggests that genuine changes in one neuroimaging marker can directly influence another (e.g., enlargement of lateral ventricles). This finding strongly highlights the need for multimodal longitudinal strategies to gain a more comprehensive understanding of the synergistic role of cerebrovascular and neurodegenerative processes.

### Cortical thinning

The thickness of the cerebral cortex decreased over the course of four years, corroborating that ageing also drives cortical thinning [7, 59]. The rate at which thinning occurred was nonetheless subject- and region-specific. The cingulate cortex underwent the fastest thinning over four years, with an average rate of 0.008 [95%-CI 0.007, 0.009] mm/year. This apparent ageing-related vulnerability is consistent with previous research indicating that both the caudal anterior and posterior cingulate cortex shrink during normal ageing [60]. The behavioural consequences of the rate of thinning in terms of decline in cognitive control and integrating behavioural, affective, and cognitive processes [61] remain to be elucidated.

Cortical thinning showed considerable heterogeneity across subjects. Somewhat surprisingly, such inter-subject variability could not be fully explained by age, sex, years of education, or cardiovascular risk factors. This finding ultimately suggests that other factors, such as genetics and lifestyle factors beyond cardiovascular risk factors [10–13], might influence cortical thinning during late life, possibly to a larger extent than demographics and established cardiovascular risk factors. Given that the rate of thinning might affect cognitive performance and activities of daily living, future research should determine the contribution of brain resilience and (modifiable) lifestyle factors to abnormal cortical thinning, as such findings could advance the development of novel interventions [62].

### Co-occurrence beyond common risk factors

Even after adjusting for shared risk factors, we found evidence for a negative correlation between the initial thickness of the cerebral cortex and the initial volume of WMH, in line with previous work [3, 6, 10, 23–25]. While other factors may contribute to this relationship—which we did not include in our analysis (e.g., genetics and lifestyle)—this observation, found in a relatively healthy sample, suggests shared underlying pathological mechanisms.

### WMH and cortical thinning

WMH volumes partially accounted for the rate of cortical thinning across the entire brain over the course of four years, particularly in the temporal, cingulate, and insular cortices. This observation is consistent with the cerebrovascular hypothesis [1, 63–65] and supports the notion that WMH are the visible tip of the iceberg [1], a sign of widespread rather than focal cerebrovascular and metabolic impairment [66, 67].

The apparent region-specific nature of the coupling between WMH volume and regional cortical thickness raises the possibility that white matter fibres could be involved in the downstream effects of WMH. Potential secondary effects of WMH along the inferior fronto-occipital fasciculus may, for instance, explain why individuals could experience rapid thinning concurrently across the temporal, cingulate, and insular cortical regions. Mounting data indeed suggests that abnormal tissue characteristics can be found in intra- and perilesional white matter regions, but also in white matter fibres traversing WMH [1, 27, 67, 68]. Also, cross-sectional investigations conducted in CSVD cohorts have demonstrated that cortical regions connected to incident lacunes, subcortical lacunar infarcts, and WMH through white matter fibres exhibit significantly reduced thickness than those that are not [30, 63–65]. Despite the overall compelling evidence for a contribution of WMH to cortical thinning, additional research leveraging imaging techniques like white matter tractography as well as animal models is needed to shed light on the role of white matter fibres in the long-term and remote effects of WMH in the brain.

### Cortical thickness and WMH progression

The progression of WMH over four years was partly explained by the thickness of the cerebral cortex, with slower WMH progression occurring in individuals with thicker global, temporal, cingulate, and insular cortical thicknesses at baseline. This simultaneous association may indicate potentially higher brain maintenance as a mechanism of healthy ageing [69] and may be multifaceted. Neuronal loss in these cortical regions may be linked to lifestyle adaptations stemming from ageing that contribute to a decline in social interactions, emotional responses, and the integration of sensory information [70–72]. Considering the involvement of the insular cortex in the regulation of autonomic functions, a decline in this region could also result in blood pressure dysregulation [73, 74], a condition which has been extensively shown to be associated with increased progression of WMH, and with more severe manifestations of CSVD [28, 55, 75].

The association between baseline cortical thickness and WMH progression has a fundamental ramification: it supports the multi-factorial origin of WMH, with neurodegeneration contributing to the progression of WMH. Since cortical neurodegeneration accelerates with the pathophysiology of AD, this would explain why posterior WMH appear in subjects with minimal vascular pathology across the AD spectrum and why WMH in deep and periventricular posterior regions appear characteristics of AD [26, 36, 38, 76]. It is also possible that an early (preclinical) increase in biomarkers indicative for AD may cause changes in the insular cortex, which then affects the cardiovascular system [73, 74, 77] and ultimately speeds up the progression of WMH in the brain—a possible explanation for Fig. 3B Q3. While promising, further research in other cohorts—especially with available amyloid- or tau- positron emission tomography [78]—are needed to determine how age- and AD-driven cortical neurodegeneration influences WMH [76].

### WMH progression and cortical thinning

WMH progression and cortical thinning were associated with one another, suggesting a rather consistent and predictable relationship between the two processes, wherein changes in one marker are accompanied by corresponding changes in the other and vice versa. In our group of cognitively unimpaired participants, this slope-slope association was particularly evident across frontal, occipital, and insular brain regions. This pattern seems even more widespread with advanced stages of AD, as highlighted in a recent work with autosomal dominant AD and late-onset AD [33]. Further application of our methodology to cohorts at various stages of AD could, for example, provide further information on the mechanisms underlying the simultaneous progression of both processes.

### Strengths and contextualisation

Longitudinal studies with cognitively unimpaired elderly participants exploring cross-domain associations between WMH and cortical thickness are scarce [1, 4, 79]. Whenever this kind of research has been done, the evidence supporting any kind of coupling has generally been lacking. In septuagenarian community-dwelling participants, Dickie et al. [4] could not find enough evidence supporting the relationship between total WMH volumes and cortical thickness of cortical grey matter structures neighbouring the Sylvian fissures over a three-year period. In a cohort of cognitively unimpaired participants, Hotz et al. [79] investigated cross-domain associations between total WMH volume and thinning of the entorhinal cortex over a duration of seven years using BLGCM. The authors found no evidence for cross-domain coupling and this absence of association was evident both at the study's baseline and throughout its duration. Evidence supporting cross-domain associations has nonetheless been growing in participants symptomatic or more severe presentations of cerebrovascular [63–65, 78, 80] and neurodegenerative pathologies [33, 78], as well as in those with neuroinflammatory conditions, such as multiple sclerosis [81, 82].

A potential explanation for such contradictory results may well lie in the stage of dysfunction at which each participant is situated, i.e., coupling only becomes evident at advanced, symptomatic stages of cerebrovascular and neurodegenerative disease. On the other hand, as emphasised by our study, there are regional nuances to these cross-domain relationships that analyses with a lower level of granularity might fail to capture. This underscores the significance of employing multimodal and regional approaches to gain a more comprehensive understanding of the local and distant effects of one process on the other.

### Limitations

Our research has four main limitations. First, even though our BLGCM aligns with the data, causality remains elusive due to model equivariance. Latent change score models might be promising for further study of specific interactions over discrete time intervals [83]. The mass-univariate application of the BLGCM could be streamlined by using extended measurement models [84]. We can state, however, that our data supports a specific and partial spatiotemporal coupling between cortical neurodegeneration and cerebrovascular dysfunction. The specific circumstances that might lead to such coupling often remain undetermined and likely require the inclusion of more extensive biological parameters including complementary imaging modalities, such as diffusion tensor imaging [27, 78, 81]. If a Wallerian-like degeneration is responsible for the observed coupling—as also discussed in the literature [3, 5, 9, 17, 26, 34, 85]—there should be evidence within the white matter fibres themselves that mediate the interrelationships between cortical thickness and WMH. Second, we considered a relatively healthy sample from a study in which cerebrovascular dysfunction is under-represented and took into account a relatively short time span (48 months, i.e., 4 years). This may have prevented a few cross-domain associations to become more evident. The dynamics over longer time periods, as well as in other cohorts remain elusive, but will be a matter of future investigation. Third, this work did not consider subcortical structures, such as the hippocampus, which may also be affected by ischaemic or hypoxic damage indicated by the presence of WMH. The BLGCM can be easily expanded to investigate the relationship between WMH and atrophy in subcortical structures, and this will be explored in future research. Fourth, we have, thus far, not assessed potential cognitive sequelae of WMH progression, cortical thinning, or their coupling in this study. Because these two processes appear to be coupled prior to any observable objective cognitive deficiencies, it could be that cognitive consequences are not detectable at this asymptomatic stage or that cognitive reserve is still able to compensate for the ongoing pathology or, as a recent study suggests, that cortical measurements predict well chronological age but not memory performance [86]. A trivariate latent change score model with WMH, cortical thickness, and cognitive performance could be used in the future to address this limitation.

## Conclusion

Our work provides longitudinal evidence that cortical thinning and WMH progression could be mutually reinforcing as opposed to parallel, disassociated processes. The coupling between these two neuroradiological features appears to be entangled prior to the onset of any detectable cognitive deficits. Our findings support the ongoing discussion on perilesional and remote impacts of WMH, but, at the same time, provide evidence for the effects of cortical neurodegeneration on white matter integrity. Comprehensive, multimodal approaches, such as the one applied in this study, have the potential to facilitate the detection of downstream damage associated with the synergistic interaction among ageing, CSVD, and neurodegeneration in the brain.

## Supplementary Information

Supplementary Material 1.

Supplementary Material 2.

## Data Availability

No datasets were generated or analysed during the current study.

## Abbreviations

AD: Alzheimer’s Disease
BLGCM: Bivariate Latent Growth Curve Model
CERAD: Consortium to Establish a Registry for AD
CFI: Comparative Fit Index
CI: Confidence Interval
CSF: Cerebrospinal Fluid
CSVD: Cerebral Small Vessel Disease
DELCODE: DZNE Longitudinal Cognitive Impairment and Dementia Study
DZNE: German Centre for Neurodegenerative Diseases
FDR: False Discovery Rate
IQR: Inter-Quartile Range
LGCM: Latent Growth Curve Model
LME: Linear Mixed-Effect Model
MRI: Magnetic Resonance Imaging
RMSEA: Root Mean Square Error of Approximation
SE: Standard Error
SEM: Structural Equation Model
SRMR: Standardised Root Mean Residual
TICV: Total Intracranial Volume
WMH: White Matter Hyperintensities
