# Supplementary material for: Longitudinal evidence for a mutually reinforcing relationship between white matter hyperintensities and cortical thickness in cognitively unimpaired older adults
Source: Alzheimers Res Ther. 2024 Oct 28;16:240. doi: 10.1186/s13195-024-01606-5 (PMC11520063; doi:10.1186/s13195-024-01606-5)
Supplement: Supplementary file 1 — Supplementary Material 1. [file 13195_2024_1606_MOESM1_ESM.pdf]

Additional File 1 – Supplementary Figures and Tables

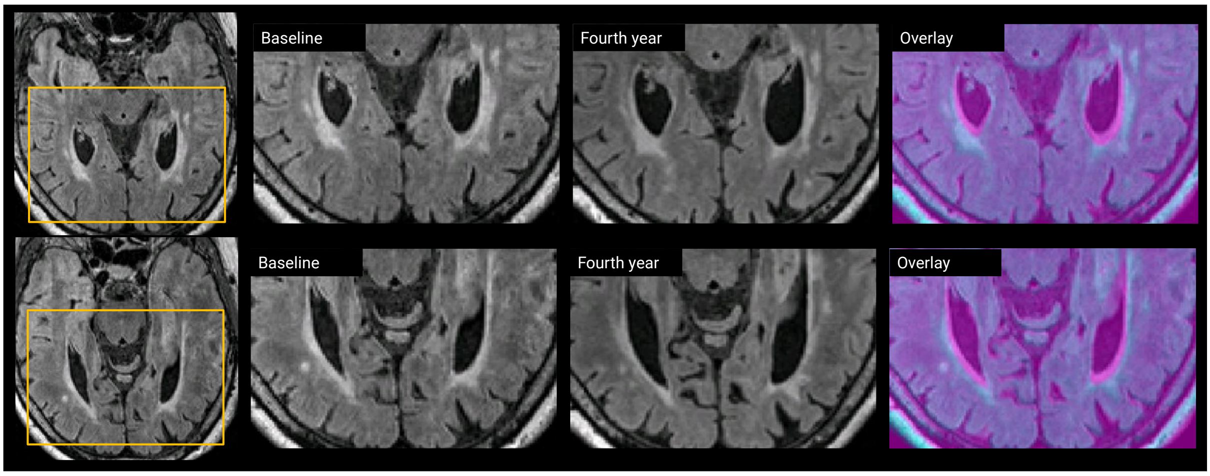

Supplementary Figure 1. Subject with regression of occipital WMH volumes over the course of four years. The expansion of the lateral ventricles (highlighted in purple on the overlay image) causes tissue loss, which ultimately leads to a reduction in occipital WMH volumes.

Supplementary Table 1. Number of data points excluded prior to modelling.

Variable of interest	Number of excluded data points	Patients from which data points were excluded
Total WMH volumes	7 (0.38)	5
Mean cortical thickness	55 (3.03)	32
Frontal cortical thickness	49 (2.69)	31
Parietal cortical thickness	40 (2.20)	25
Occipital cortical thickness	21 (1.57)	16
Temporal cortical thickness	57 (3.14)	35
Cingulate cortical thickness	56 (3.08)	29
Insular cortical thickness	57 (3.14)	27
