# Supplementary material for: Longitudinal evidence for a mutually reinforcing relationship between white matter hyperintensities and cortical thickness in cognitively unimpaired older adults
Source: Alzheimers Res Ther. 2024 Oct 28;16:240. doi: 10.1186/s13195-024-01606-5 (PMC11520063; doi:10.1186/s13195-024-01606-5)
Supplement: Supplementary file 2 — Supplementary Material 2. [file 13195_2024_1606_MOESM2_ESM.pdf]

Additional File 2 – LGCM summaries

ULGCM - Total WMH volumes

Unstandardised solution

```
lavaan 0.6.17 ended normally after 63 iterations

  Estimator                                         ML
  Optimization method                           NLMINB
  Number of model parameters                        30
  Number of equality constraints                     4

  Number of observations                           451
  Number of missing patterns                        19

Model Test User Model:
                                              Standard      Scaled
  Test Statistic                                37.344      30.634
  Degrees of freedom                                39          39
  P-value (Chi-square)                           0.546       0.828
  Scaling correction factor                                  1.219
    Yuan-Bentler correction (Mplus variant)

Model Test Baseline Model:

  Test statistic                              4880.299    3552.411
  Degrees of freedom                                45          45
  P-value                                        0.000       0.000
  Scaling correction factor                                  1.374

User Model versus Baseline Model:

  Comparative Fit Index (CFI)                    1.000       1.000
  Tucker-Lewis Index (TLI)                       1.000       1.003

  Robust Comparative Fit Index (CFI)                         1.000
  Robust Tucker-Lewis Index (TLI)                            1.003

Loglikelihood and Information Criteria:

  Loglikelihood user model (H0)              -3307.490   -3307.490
  Scaling correction factor                                  1.044
```

```
    for the MLR correction
  Loglikelihood unrestricted model (H1)      -3288.818   -3288.818
  Scaling correction factor                                 1.213
    for the MLR correction

  Akaike (AIC)                                6666.980    6666.980
  Bayesian (BIC)                              6773.878    6773.878
  Sample-size adjusted Bayesian (SABIC)       6691.363    6691.363

Root Mean Square Error of Approximation:

  RMSEA                                          0.000       0.000
  90 Percent confidence interval - lower         0.000       0.000
  90 Percent confidence interval - upper         0.031       0.018
  P-value H_0: RMSEA <= 0.050                    1.000       1.000
  P-value H_0: RMSEA >= 0.080                    0.000       0.000

  Robust RMSEA                                               0.000
  90 Percent confidence interval - lower                     0.000
  90 Percent confidence interval - upper                     0.024
  P-value H_0: Robust RMSEA <= 0.050                         0.999
  P-value H_0: Robust RMSEA >= 0.080                         0.000

Standardized Root Mean Square Residual:

  SRMR                                           0.009       0.009

Parameter Estimates:

  Standard errors                             Sandwich
  Information bread                           Observed
  Observed information based on                Hessian

Latent Variables:
                   Estimate  Std.Err  z-value  P(>|z|)   Std.lv  Std.all
  iy =~
    y1                1.000                               0.998    0.991
    y2                1.000                               0.998    0.990
    y3                1.000                               0.998    0.984
    y4                1.000                               0.998    0.975
    y5                1.000                               0.998    0.963
  sy =~
    y1                0.000                               0.000    0.000
    y2                1.000                               0.063    0.062
    y3                2.000                               0.126    0.124
```

```
    y4                3.000                               0.189    0.185
    y5                4.000                               0.252    0.243

Regressions:
                   Estimate  Std.Err  z-value  P(>|z|)   Std.lv  Std.all
  iy ~
    age_M00           0.373    0.044    8.440    0.000    0.374    0.374
    sex               0.188    0.060    3.129    0.002    0.189    0.189
    edyears          -0.027    0.044   -0.620    0.535   -0.027   -0.027
    vsclr_rsk_smcr    0.101    0.045    2.271    0.023    0.102    0.102
    eTIV              0.259    0.056    4.639    0.000    0.260    0.260
  sy ~
    age_M00          -0.005    0.004   -1.094    0.274   -0.076   -0.076
    sex               0.000    0.006    0.080    0.937    0.007    0.007
    edyears          -0.004    0.004   -1.063    0.288   -0.071   -0.071
    vsclr_rsk_smcr   -0.001    0.004   -0.281    0.779   -0.019   -0.019
    eTIV             -0.002    0.005   -0.362    0.717   -0.028   -0.028

Covariances:
                           Estimate  Std.Err  z-value  P(>|z|)   Std.lv  Std.all
 .iy ~~
   .sy                        0.002    0.004    0.425    0.671    0.030    0.030
  age_M00 ~~
    sex                      -0.165    0.046   -3.607    0.000   -0.165   -0.165
    edyears                  -0.119    0.048   -2.478    0.013   -0.119   -0.119
    vsclr_rsk_smcr            0.157    0.047    3.335    0.001    0.157    0.157
    eTIV                      0.057    0.046    1.234    0.217    0.057    0.057
  sex ~~
    edyears                  -0.238    0.043   -5.569    0.000   -0.238   -0.238
    vsclr_rsk_smcr           -0.184    0.045   -4.128    0.000   -0.184   -0.184
    eTIV                     -0.668    0.021  -31.365    0.000   -0.668   -0.668
  edyears ~~
    vsclr_rsk_smcr           -0.156    0.043   -3.625    0.000   -0.156   -0.156
    eTIV                      0.247    0.042    5.956    0.000    0.247    0.247
  vascular_risk_sumcorr ~~
    eTIV                      0.113    0.048    2.362    0.018    0.113    0.113

Intercepts:
                   Estimate  Std.Err  z-value  P(>|z|)   Std.lv  Std.all
   .iy               -0.130    0.043   -3.064    0.002   -0.131   -0.131
   .sy                0.070    0.004   17.701    0.000    1.117    1.117
   .y1                0.000                               0.000    0.000
   .y2                0.000                               0.000    0.000
   .y3                0.000                               0.000    0.000
   .y4                0.000                               0.000    0.000
```

```
   .y5                0.000                                  0.000    0.000
    age_M00           0.000                                  0.000    0.000
    edyears           0.000                                  0.000    0.000
    sex               0.000                                  0.000    0.000
    vsclr_rsk_smcr    0.000                                  0.000    0.000
    eTIV              0.000                                  0.000    0.000

Variances:
                   Estimate  Std.Err  z-value  P(>|z|)   Std.lv  Std.all
   .iy                0.808    0.049   16.427    0.000    0.811    0.811
   .sy                0.004    0.001    4.907    0.000    0.987    0.987
   .y1      (tht_)    0.018    0.002   10.481    0.000    0.018    0.017
   .y2      (tht_)    0.018    0.002   10.481    0.000    0.018    0.017
   .y3      (tht_)    0.018    0.002   10.481    0.000    0.018    0.017
   .y4      (tht_)    0.018    0.002   10.481    0.000    0.018    0.017
   .y5      (tht_)    0.018    0.002   10.481    0.000    0.018    0.016
    age_M00           1.000                                  1.000    1.000
    sex               1.000                                  1.000    1.000
    edyears           1.000                                  1.000    1.000
    vsclr__           1.000                                  1.000    1.000
    eTIV              1.000                                  1.000    1.000

R-Square:
                   Estimate
    iy                0.189
    sy                0.013
    y1                0.983
    y2                0.983
    y3                0.983
    y4                0.983
    y5                0.984
```

Standardised solution

```
Latent Variables:
                    est.std  Std.Err  z-value  P(>|z|) ci.lower ci.upper
  iy =~
    y1                0.991    0.001 1072.923    0.000    0.989    0.993
    y2                0.990    0.005  216.734    0.000    0.981    0.999
    y3                0.984    0.008  116.090    0.000    0.968    1.001
    y4                0.975    0.012   80.829    0.000    0.951    0.999
    y5                0.963    0.015   62.862    0.000    0.933    0.993
  sy =~
    y1                0.000                                  0.000    0.000
```

	est.std	Std.Err	z-value	P(>|z|)	ci.lower	ci.upper
y2	0.062	0.007	9.555	0.000	0.050	0.075
y3	0.124	0.013	9.493	0.000	0.099	0.150
y4	0.185	0.019	9.492	0.000	0.146	0.223
y5	0.243	0.025	9.550	0.000	0.193	0.293

Regressions:

	est.std	Std.Err	z-value	P(>|z|)	ci.lower	ci.upper
iy ~						
age_M00	0.374	0.042	8.932	0.000	0.292	0.456
sex	0.189	0.060	3.161	0.002	0.072	0.306
edyears	-0.027	0.044	-0.621	0.535	-0.114	0.059
vsclr_rsk_smcr	0.102	0.044	2.303	0.021	0.015	0.188
eTIV	0.260	0.054	4.786	0.000	0.154	0.366
sy ~						
age_M00	-0.076	0.069	-1.104	0.270	-0.212	0.059
sex	0.007	0.089	0.079	0.937	-0.167	0.181
edyears	-0.071	0.067	-1.053	0.292	-0.202	0.061
vsclr_rsk_smcr	-0.019	0.067	-0.281	0.779	-0.149	0.112
eTIV	-0.028	0.075	-0.366	0.715	-0.175	0.120

Covariances:

	est.std	Std.Err	z-value	P(>|z|)	ci.lower	ci.upper
.iy ~~						
.sy	0.030	0.072	0.418	0.676	-0.111	0.171
age_M00 ~~						
sex	-0.165	0.046	-3.607	0.000	-0.255	-0.075
edyears	-0.119	0.048	-2.478	0.013	-0.214	-0.025
vsclr_rsk_smcr	0.157	0.047	3.335	0.001	0.065	0.249
eTIV	0.057	0.046	1.234	0.217	-0.033	0.146
sex ~~						
edyears	-0.238	0.043	-5.569	0.000	-0.321	-0.154
vsclr_rsk_smcr	-0.184	0.045	-4.128	0.000	-0.272	-0.097
eTIV	-0.668	0.021	-31.365	0.000	-0.710	-0.626
edyears ~~						
vsclr_rsk_smcr	-0.156	0.043	-3.625	0.000	-0.241	-0.072
eTIV	0.247	0.042	5.956	0.000	0.166	0.329
vascular_risk_sumcorr ~~						
eTIV	0.113	0.048	2.362	0.018	0.019	0.207

Intercepts:

	est.std	Std.Err	z-value	P(>|z|)	ci.lower	ci.upper
.iy	-0.131	0.043	-3.043	0.002	-0.215	-0.047
.sy	1.117	0.110	10.177	0.000	0.902	1.332
.y1	0.000				0.000	0.000
.y2	0.000				0.000	0.000

```
   .y3                0.000                                   0.000    0.000
   .y4                0.000                                   0.000    0.000
   .y5                0.000                                   0.000    0.000
    age_M00           0.000                                   0.000    0.000
    edyears           0.000                                   0.000    0.000
    sex               0.000                                   0.000    0.000
    vsclr_rsk_smcr    0.000                                   0.000    0.000
    eTIV              0.000                                   0.000    0.000

Variances:
                    est.std  Std.Err  z-value  P(>|z|) ci.lower ci.upper
   .iy                0.811    0.032   25.316    0.000    0.748    0.874
   .sy                0.987    0.015   67.281    0.000    0.958    1.016
   .y1      (tht_)    0.017    0.002    9.467    0.000    0.014    0.021
   .y2      (tht_)    0.017    0.002    9.596    0.000    0.014    0.021
   .y3      (tht_)    0.017    0.002    9.667    0.000    0.014    0.021
   .y4      (tht_)    0.017    0.002    9.683    0.000    0.013    0.020
   .y5      (tht_)    0.016    0.002    9.650    0.000    0.013    0.020
    age_M00           1.000                                   1.000    1.000
    sex               1.000                                   1.000    1.000
    edyears           1.000                                   1.000    1.000
    vsclr__           1.000                                   1.000    1.000
    eTIV              1.000                                   1.000    1.000
```

ULGCM – Mean cortical thickness

Unstandardised solution

```
lavaan 0.6.17 ended normally after 47 iterations

  Estimator                                         ML
  Optimization method                           NLMINB
  Number of model parameters                        30
  Number of equality constraints                     4

  Number of observations                           451
  Number of missing patterns                        28

Model Test User Model:
                                              Standard      Scaled
  Test Statistic                                46.524      36.099
  Degrees of freedom                                39          39
  P-value (Chi-square)                           0.190       0.603
  Scaling correction factor                                  1.289
    Yuan-Bentler correction (Mplus variant)

Model Test Baseline Model:

  Test statistic                              2522.398    1760.939
  Degrees of freedom                                45          45
  P-value                                        0.000       0.000
  Scaling correction factor                                  1.432

User Model versus Baseline Model:

  Comparative Fit Index (CFI)                    0.997       1.000
  Tucker-Lewis Index (TLI)                       0.996       1.002

  Robust Comparative Fit Index (CFI)                         1.000
  Robust Tucker-Lewis Index (TLI)                            1.002

Loglikelihood and Information Criteria:

  Loglikelihood user model (H0)              -4370.946   -4370.946
  Scaling correction factor                                  1.069
      for the MLR correction
  Loglikelihood unrestricted model (H1)      -4347.684   -4347.684
  Scaling correction factor                                  1.267
```

```
      for the MLR correction

  Akaike (AIC)                                8793.891    8793.891
  Bayesian (BIC)                              8900.790    8900.790
  Sample-size adjusted Bayesian (SABIC)       8818.275    8818.275

Root Mean Square Error of Approximation:

  RMSEA                                          0.021       0.000
  90 Percent confidence interval - lower         0.000       0.000
  90 Percent confidence interval - upper         0.040       0.026
  P-value H_0: RMSEA <= 0.050                    0.996       1.000
  P-value H_0: RMSEA >= 0.080                    0.000       0.000

  Robust RMSEA                                               0.000
  90 Percent confidence interval - lower                     0.000
  90 Percent confidence interval - upper                     0.041
  P-value H_0: Robust RMSEA <= 0.050                         0.985
  P-value H_0: Robust RMSEA >= 0.080                         0.000

Standardized Root Mean Square Residual:

  SRMR                                           0.014       0.014

Parameter Estimates:

  Standard errors                             Sandwich
  Information bread                           Observed
  Observed information based on                Hessian

Latent Variables:
                   Estimate  Std.Err  z-value  P(>|z|)   Std.lv  Std.all
  ix =~
    x1                1.000                               0.926    0.939
    x2                1.000                               0.926    0.928
    x3                1.000                               0.926    0.909
    x4                1.000                               0.926    0.885
    x5                1.000                               0.926    0.857
  sx =~
    x1                0.000                               0.000    0.000
    x2                1.000                               0.091    0.092
    x3                2.000                               0.183    0.180
    x4                3.000                               0.274    0.262
    x5                4.000                               0.366    0.338
```

```
Regressions:
                   Estimate  Std.Err  z-value  P(>|z|)   Std.lv  Std.all
  ix ~
    age_M00          -0.309    0.046   -6.663    0.000   -0.334   -0.334
    sex              -0.031    0.060   -0.514    0.608   -0.033   -0.033
    edyears          -0.043    0.047   -0.920    0.358   -0.047   -0.047
    vsclr_rsk_smcr   -0.073    0.047   -1.548    0.122   -0.079   -0.079
    eTIV             -0.114    0.061   -1.854    0.064   -0.123   -0.123
  sx ~
    age_M00          -0.025    0.008   -3.055    0.002   -0.275   -0.275
    sex               0.009    0.010    0.823    0.411    0.093    0.093
    edyears           0.009    0.007    1.252    0.211    0.096    0.096
    vsclr_rsk_smcr    0.005    0.008    0.640    0.522    0.055    0.055
    eTIV              0.002    0.010    0.151    0.880    0.017    0.017

Covariances:
                           Estimate  Std.Err  z-value  P(>|z|)   Std.lv  Std.all
 .ix ~~
   .sx                       -0.000    0.009   -0.041    0.968   -0.005   -0.005
  age_M00 ~~
    sex                      -0.165    0.046   -3.607    0.000   -0.165   -0.165
    edyears                  -0.119    0.048   -2.478    0.013   -0.119   -0.119
    vsclr_rsk_smcr            0.157    0.047    3.335    0.001    0.157    0.157
    eTIV                      0.057    0.046    1.234    0.217    0.057    0.057
  sex ~~
    edyears                  -0.238    0.043   -5.569    0.000   -0.238   -0.238
    vsclr_rsk_smcr           -0.184    0.045   -4.128    0.000   -0.184   -0.184
    eTIV                     -0.668    0.021  -31.365    0.000   -0.668   -0.668
  edyears ~~
    vsclr_rsk_smcr           -0.156    0.043   -3.625    0.000   -0.156   -0.156
    eTIV                      0.247    0.042    5.956    0.000    0.247    0.247
  vascular_risk_sumcorr ~~
    eTIV                      0.113    0.048    2.362    0.018    0.113    0.113

Intercepts:
                   Estimate  Std.Err  z-value  P(>|z|)   Std.lv  Std.all
   .ix                0.028    0.044    0.630    0.529    0.030    0.030
   .sx               -0.019    0.008   -2.403    0.016   -0.206   -0.206
   .x1                0.000                                0.000    0.000
   .x2                0.000                                0.000    0.000
   .x3                0.000                                0.000    0.000
   .x4                0.000                                0.000    0.000
   .x5                0.000                                0.000    0.000
    age_M00           0.000                                0.000    0.000
    edyears           0.000                                0.000    0.000
```

```
    sex               0.000                                    0.000    0.000
    vsclr_rsk_smcr    0.000                                    0.000    0.000
    eTIV              0.000                                    0.000    0.000

Variances:
                   Estimate  Std.Err  z-value  P(>|z|)   Std.lv  Std.all
   .ix                0.739    0.062   11.992    0.000    0.862    0.862
   .sx                0.008    0.003    2.835    0.005    0.902    0.902
   .x1      (tht_)    0.115    0.011   10.459    0.000    0.115    0.118
   .x2      (tht_)    0.115    0.011   10.459    0.000    0.115    0.116
   .x3      (tht_)    0.115    0.011   10.459    0.000    0.115    0.111
   .x4      (tht_)    0.115    0.011   10.459    0.000    0.115    0.105
   .x5      (tht_)    0.115    0.011   10.459    0.000    0.115    0.099
    age_M00           1.000                                    1.000    1.000
    sex               1.000                                    1.000    1.000
    edyears           1.000                                    1.000    1.000
    vsclr__           1.000                                    1.000    1.000
    eTIV              1.000                                    1.000    1.000

R-Square:
                   Estimate
    ix                0.138
    sx                0.098
    x1                0.882
    x2                0.884
    x3                0.889
    x4                0.895
    x5                0.901
```

Standardised solution

```
Latent Variables:
                    est.std  Std.Err  z-value  P(>|z|) ci.lower ci.upper
  ix =~
    x1                0.939    0.007  133.127    0.000    0.925    0.953
    x2                0.928    0.011   80.874    0.000    0.905    0.950
    x3                0.909    0.017   52.130    0.000    0.875    0.944
    x4                0.885    0.023   38.461    0.000    0.840    0.930
    x5                0.857    0.028   30.493    0.000    0.802    0.912
  sx =~
    x1                0.000                                0.000    0.000
    x2                0.092    0.016    5.768    0.000    0.060    0.123
    x3                0.180    0.031    5.792    0.000    0.119    0.240
    x4                0.262    0.044    5.894    0.000    0.175    0.349
```

```
    x5                0.338    0.056    6.066    0.000    0.229    0.448

Regressions:
                   est.std  Std.Err  z-value  P(>|z|) ci.lower ci.upper
  ix ~
    age_M00         -0.334    0.048   -6.964    0.000   -0.428   -0.240
    sex             -0.033    0.064   -0.515    0.607   -0.159    0.093
    edyears         -0.047    0.051   -0.926    0.355   -0.146    0.052
    vsclr_rsk_smcr  -0.079    0.051   -1.561    0.118   -0.178    0.020
    eTIV            -0.123    0.066   -1.866    0.062   -0.252    0.006
  sx ~
    age_M00         -0.275    0.082   -3.338    0.001   -0.437   -0.114
    sex              0.093    0.111    0.843    0.400   -0.124    0.310
    edyears          0.096    0.077    1.254    0.210   -0.054    0.247
    vsclr_rsk_smcr   0.055    0.087    0.626    0.531   -0.116    0.226
    eTIV             0.017    0.112    0.151    0.880   -0.203    0.237

Covariances:
                           est.std  Std.Err  z-value  P(>|z|) ci.lower ci.upper
 .ix ~~
   .sx                      -0.005    0.118   -0.041    0.967   -0.237    0.227
  age_M00 ~~
    sex                     -0.165    0.046   -3.607    0.000   -0.255   -0.075
    edyears                 -0.119    0.048   -2.478    0.013   -0.214   -0.025
    vsclr_rsk_smcr           0.157    0.047    3.335    0.001    0.065    0.249
    eTIV                     0.057    0.046    1.234    0.217   -0.033    0.146
  sex ~~
    edyears                 -0.238    0.043   -5.569    0.000   -0.321   -0.154
    vsclr_rsk_smcr          -0.184    0.045   -4.128    0.000   -0.272   -0.097
    eTIV                    -0.668    0.021  -31.365    0.000   -0.710   -0.626
  edyears ~~
    vsclr_rsk_smcr          -0.156    0.043   -3.625    0.000   -0.241   -0.072
    eTIV                     0.247    0.042    5.956    0.000    0.166    0.329
  vascular_risk_sumcorr ~~
    eTIV                     0.113    0.048    2.362    0.018    0.019    0.207

Intercepts:
                   est.std  Std.Err  z-value  P(>|z|) ci.lower ci.upper
   .ix               0.030    0.048    0.630    0.529   -0.063    0.123
   .sx              -0.206    0.096   -2.152    0.031   -0.393   -0.018
   .x1               0.000                                0.000    0.000
   .x2               0.000                                0.000    0.000
   .x3               0.000                                0.000    0.000
   .x4               0.000                                0.000    0.000
   .x5               0.000                                0.000    0.000
```

```
    age_M00           0.000                                   0.000    0.000
    edyears           0.000                                   0.000    0.000
    sex               0.000                                   0.000    0.000
    vsclr_rsk_smcr    0.000                                   0.000    0.000
    eTIV              0.000                                   0.000    0.000

Variances:
                   est.std  Std.Err  z-value  P(>|z|) ci.lower ci.upper
   .ix               0.862    0.034   25.643    0.000    0.796    0.928
   .sx               0.902    0.051   17.837    0.000    0.803    1.002
   .x1      (tht_)   0.118    0.013    8.938    0.000    0.092    0.144
   .x2      (tht_)   0.116    0.013    9.101    0.000    0.091    0.140
   .x3      (tht_)   0.111    0.012    9.174    0.000    0.087    0.135
   .x4      (tht_)   0.105    0.011    9.162    0.000    0.083    0.128
   .x5      (tht_)   0.099    0.011    9.041    0.000    0.077    0.120
    age_M00          1.000                                1.000    1.000
    sex              1.000                                1.000    1.000
    edyears          1.000                                1.000    1.000
    vsclr__          1.000                                1.000    1.000
    eTIV             1.000                                1.000    1.000
```

ULGCM – Frontal cortical thickness

Unstandardised solution

```
lavaan 0.6.17 ended normally after 46 iterations

  Estimator                                         ML
  Optimization method                           NLMINB
  Number of model parameters                        30
  Number of equality constraints                     4

  Number of observations                           451
  Number of missing patterns                        29

Model Test User Model:
                                              Standard      Scaled
  Test Statistic                                36.756      30.836
  Degrees of freedom                                39          39
  P-value (Chi-square)                           0.573       0.822
  Scaling correction factor                                  1.192
    Yuan-Bentler correction (Mplus variant)

Model Test Baseline Model:

  Test statistic                              2456.122    1879.030
  Degrees of freedom                                45          45
  P-value                                        0.000       0.000
  Scaling correction factor                                  1.307

User Model versus Baseline Model:

  Comparative Fit Index (CFI)                    1.000       1.000
  Tucker-Lewis Index (TLI)                       1.001       1.005

  Robust Comparative Fit Index (CFI)                         1.000
  Robust Tucker-Lewis Index (TLI)                            1.005

Loglikelihood and Information Criteria:

  Loglikelihood user model (H0)              -4406.977   -4406.977
  Scaling correction factor                                  0.997
      for the MLR correction
  Loglikelihood unrestricted model (H1)      -4388.599   -4388.599
  Scaling correction factor                                  1.176
```

```
    for the MLR correction

  Akaike (AIC)                                8865.954    8865.954
  Bayesian (BIC)                              8972.853    8972.853
  Sample-size adjusted Bayesian (SABIC)       8890.338    8890.338

Root Mean Square Error of Approximation:

  RMSEA                                          0.000       0.000
  90 Percent confidence interval - lower         0.000       0.000
  90 Percent confidence interval - upper         0.030       0.018
  P-value H_0: RMSEA <= 0.050                    1.000       1.000
  P-value H_0: RMSEA >= 0.080                    0.000       0.000

  Robust RMSEA                                               0.000
  90 Percent confidence interval - lower                     0.000
  90 Percent confidence interval - upper                     0.028
  P-value H_0: Robust RMSEA <= 0.050                         0.998
  P-value H_0: Robust RMSEA >= 0.080                         0.000

Standardized Root Mean Square Residual:

  SRMR                                           0.015       0.015

Parameter Estimates:

  Standard errors                             Sandwich
  Information bread                           Observed
  Observed information based on                Hessian

Latent Variables:
                   Estimate  Std.Err  z-value  P(>|z|)   Std.lv  Std.all
  ix =~
    x1                1.000                               0.924    0.936
    x2                1.000                               0.924    0.925
    x3                1.000                               0.924    0.907
    x4                1.000                               0.924    0.883
    x5                1.000                               0.924    0.854
  sx =~
    x1                0.000                               0.000    0.000
    x2                1.000                               0.094    0.094
    x3                2.000                               0.188    0.185
    x4                3.000                               0.282    0.270
    x5                4.000                               0.376    0.348
```

Regressions:

	Estimate	Std.Err	z-value	P(>|z|)	Std.lv	Std.all
ix ~						
age_M00	-0.325	0.045	-7.247	0.000	-0.351	-0.351
sex	-0.031	0.061	-0.517	0.605	-0.034	-0.034
edyears	-0.070	0.047	-1.477	0.140	-0.076	-0.076
vsclr_rsk_smcr	-0.081	0.048	-1.698	0.089	-0.087	-0.087
eTIV	-0.130	0.061	-2.152	0.031	-0.141	-0.141
sx ~						
age_M00	-0.013	0.008	-1.536	0.125	-0.137	-0.137
sex	0.008	0.010	0.764	0.445	0.084	0.084
edyears	0.008	0.008	0.965	0.334	0.082	0.082
vsclr_rsk_smcr	0.006	0.009	0.712	0.476	0.065	0.065
eTIV	0.004	0.010	0.401	0.688	0.044	0.044

Covariances:

	Estimate	Std.Err	z-value	P(>|z|)	Std.lv	Std.all
.ix ~~						
.sx	0.003	0.009	0.335	0.738	0.040	0.040
age_M00 ~~						
sex	-0.165	0.046	-3.607	0.000	-0.165	-0.165
edyears	-0.119	0.048	-2.478	0.013	-0.119	-0.119
vsclr_rsk_smcr	0.157	0.047	3.335	0.001	0.157	0.157
eTIV	0.057	0.046	1.234	0.217	0.057	0.057
sex ~~						
edyears	-0.238	0.043	-5.569	0.000	-0.238	-0.238
vsclr_rsk_smcr	-0.184	0.045	-4.128	0.000	-0.184	-0.184
eTIV	-0.668	0.021	-31.365	0.000	-0.668	-0.668
edyears ~~						
vsclr_rsk_smcr	-0.156	0.043	-3.625	0.000	-0.156	-0.156
eTIV	0.247	0.042	5.956	0.000	0.247	0.247
vascular_risk_sumcorr ~~						
eTIV	0.113	0.048	2.362	0.018	0.113	0.113

Intercepts:

	Estimate	Std.Err	z-value	P(>|z|)	Std.lv	Std.all
.ix	-0.006	0.044	-0.138	0.890	-0.007	-0.007
.sx	0.004	0.008	0.482	0.630	0.041	0.041
.x1	0.000				0.000	0.000
.x2	0.000				0.000	0.000
.x3	0.000				0.000	0.000
.x4	0.000				0.000	0.000
.x5	0.000				0.000	0.000
age_M00	0.000				0.000	0.000
edyears	0.000				0.000	0.000

```
    sex                0.000                                    0.000    0.000
    vsclr_rsk_smcr     0.000                                    0.000    0.000
    eTIV               0.000                                    0.000    0.000

Variances:
                   Estimate  Std.Err  z-value  P(>|z|)   Std.lv  Std.all
   .ix                0.718    0.057   12.541    0.000    0.840    0.840
   .sx                0.009    0.002    3.521    0.000    0.968    0.968
   .x1      (tht_)    0.121    0.010   12.028    0.000    0.121    0.124
   .x2      (tht_)    0.121    0.010   12.028    0.000    0.121    0.121
   .x3      (tht_)    0.121    0.010   12.028    0.000    0.121    0.116
   .x4      (tht_)    0.121    0.010   12.028    0.000    0.121    0.110
   .x5      (tht_)    0.121    0.010   12.028    0.000    0.121    0.103
    age_M00           1.000                               1.000    1.000
    sex               1.000                               1.000    1.000
    edyears           1.000                               1.000    1.000
    vsclr__           1.000                               1.000    1.000
    eTIV              1.000                               1.000    1.000

R-Square:
                   Estimate
    ix                0.160
    sx                0.032
    x1                0.876
    x2                0.879
    x3                0.884
    x4                0.890
    x5                0.897
```

Standardised solution

```
Latent Variables:
                    est.std  Std.Err  z-value  P(>|z|) ci.lower ci.upper
  ix =~
    x1                0.936    0.007  137.019    0.000    0.923    0.949
    x2                0.925    0.012   76.259    0.000    0.902    0.949
    x3                0.907    0.018   49.589    0.000    0.872    0.943
    x4                0.883    0.024   37.583    0.000    0.837    0.929
    x5                0.854    0.028   30.847    0.000    0.800    0.909
  sx =~
    x1                0.000                               0.000    0.000
    x2                0.094    0.013    7.089    0.000    0.068    0.120
    x3                0.185    0.026    7.047    0.000    0.133    0.236
    x4                0.270    0.038    7.104    0.000    0.195    0.344
```

```
    x5                0.348    0.048    7.250    0.000    0.254    0.442

Regressions:
                   est.std  Std.Err  z-value  P(>|z|) ci.lower ci.upper
  ix ~
    age_M00         -0.351    0.046   -7.688    0.000   -0.441   -0.262
    sex             -0.034    0.066   -0.518    0.605   -0.163    0.095
    edyears         -0.076    0.051   -1.488    0.137   -0.175    0.024
    vsclr_rsk_smcr  -0.087    0.051   -1.728    0.084   -0.186    0.012
    eTIV            -0.141    0.065   -2.157    0.031   -0.269   -0.013
  sx ~
    age_M00         -0.137    0.089   -1.545    0.122   -0.311    0.037
    sex              0.084    0.111    0.752    0.452   -0.135    0.302
    edyears          0.082    0.082    0.998    0.318   -0.079    0.244
    vsclr_rsk_smcr   0.065    0.091    0.711    0.477   -0.114    0.243
    eTIV             0.044    0.111    0.397    0.691   -0.173    0.260

Covariances:
                           est.std  Std.Err  z-value  P(>|z|) ci.lower ci.upper
 .ix ~~
   .sx                       0.040    0.123    0.325    0.745   -0.200    0.280
  age_M00 ~~
    sex                     -0.165    0.046   -3.607    0.000   -0.255   -0.075
    edyears                 -0.119    0.048   -2.478    0.013   -0.214   -0.025
    vsclr_rsk_smcr           0.157    0.047    3.335    0.001    0.065    0.249
    eTIV                     0.057    0.046    1.234    0.217   -0.033    0.146
  sex ~~
    edyears                 -0.238    0.043   -5.569    0.000   -0.321   -0.154
    vsclr_rsk_smcr          -0.184    0.045   -4.128    0.000   -0.272   -0.097
    eTIV                    -0.668    0.021  -31.365    0.000   -0.710   -0.626
  edyears ~~
    vsclr_rsk_smcr          -0.156    0.043   -3.625    0.000   -0.241   -0.072
    eTIV                     0.247    0.042    5.956    0.000    0.166    0.329
  vascular_risk_sumcorr ~~
    eTIV                     0.113    0.048    2.362    0.018    0.019    0.207

Intercepts:
                   est.std  Std.Err  z-value  P(>|z|) ci.lower ci.upper
   .ix              -0.007    0.047   -0.138    0.890   -0.099    0.086
   .sx               0.041    0.085    0.484    0.629   -0.125    0.207
   .x1               0.000                                0.000    0.000
   .x2               0.000                                0.000    0.000
   .x3               0.000                                0.000    0.000
   .x4               0.000                                0.000    0.000
   .x5               0.000                                0.000    0.000
```

```
    age_M00           0.000                                    0.000    0.000
    edyears           0.000                                    0.000    0.000
    sex               0.000                                    0.000    0.000
    vsclr_rsk_smcr    0.000                                    0.000    0.000
    eTIV              0.000                                    0.000    0.000

Variances:
                   est.std  Std.Err  z-value  P(>|z|) ci.lower ci.upper
   .ix               0.840    0.035   24.011    0.000    0.771    0.908
   .sx               0.968    0.030   32.645    0.000    0.910    1.026
   .x1      (tht_)   0.124    0.013    9.689    0.000    0.099    0.149
   .x2      (tht_)   0.121    0.012   10.046    0.000    0.098    0.145
   .x3      (tht_)   0.116    0.011   10.148    0.000    0.094    0.139
   .x4      (tht_)   0.110    0.011   10.048    0.000    0.089    0.132
   .x5      (tht_)   0.103    0.011    9.795    0.000    0.083    0.124
    age_M00          1.000                                      1.000    1.000
    sex              1.000                                      1.000    1.000
    edyears          1.000                                      1.000    1.000
    vsclr__          1.000                                      1.000    1.000
    eTIV             1.000                                      1.000    1.000
```

UGLCM – Parietal cortical thickness

Unstandardised solution

```
lavaan 0.6.17 ended normally after 42 iterations

  Estimator                                         ML
  Optimization method                           NLMINB
  Number of model parameters                        30
  Number of equality constraints                     4

  Number of observations                           451
  Number of missing patterns                        28

Model Test User Model:
                                              Standard      Scaled
  Test Statistic                                29.240      22.340
  Degrees of freedom                                39          39
  P-value (Chi-square)                           0.872       0.985
  Scaling correction factor                                  1.309
    Yuan-Bentler correction (Mplus variant)

Model Test Baseline Model:

  Test statistic                              2424.677    1643.617
  Degrees of freedom                                45          45
  P-value                                        0.000       0.000
  Scaling correction factor                                  1.475

User Model versus Baseline Model:

  Comparative Fit Index (CFI)                    1.000       1.000
  Tucker-Lewis Index (TLI)                       1.005       1.012

  Robust Comparative Fit Index (CFI)                         1.000
  Robust Tucker-Lewis Index (TLI)                            1.011

Loglikelihood and Information Criteria:

  Loglikelihood user model (H0)              -4434.026   -4434.026
  Scaling correction factor                                  1.097
      for the MLR correction
  Loglikelihood unrestricted model (H1)      -4419.406   -4419.406
  Scaling correction factor                                  1.292
```

```
     for the MLR correction

  Akaike (AIC)                                8920.052    8920.052
  Bayesian (BIC)                              9026.950    9026.950
  Sample-size adjusted Bayesian (SABIC)       8944.436    8944.436

Root Mean Square Error of Approximation:

  RMSEA                                          0.000       0.000
  90 Percent confidence interval - lower         0.000       0.000
  90 Percent confidence interval - upper         0.017       0.000
  P-value H_0: RMSEA <= 0.050                    1.000       1.000
  P-value H_0: RMSEA >= 0.080                    0.000       0.000

  Robust RMSEA                                               0.000
  90 Percent confidence interval - lower                     0.000
  90 Percent confidence interval - upper                     0.000
  P-value H_0: Robust RMSEA <= 0.050                         1.000
  P-value H_0: Robust RMSEA >= 0.080                         0.000

Standardized Root Mean Square Residual:

  SRMR                                           0.010       0.010

Parameter Estimates:

  Standard errors                             Sandwich
  Information bread                           Observed
  Observed information based on                Hessian

Latent Variables:
                   Estimate  Std.Err  z-value  P(>|z|)   Std.lv  Std.all
  ix =~
    x1                1.000                               0.918    0.930
    x2                1.000                               0.918    0.923
    x3                1.000                               0.918    0.911
    x4                1.000                               0.918    0.893
    x5                1.000                               0.918    0.871
  sx =~
    x1                0.000                               0.000    0.000
    x2                1.000                               0.079    0.080
    x3                2.000                               0.159    0.158
    x4                3.000                               0.238    0.232
    x5                4.000                               0.318    0.302
```

```
Regressions:
                   Estimate  Std.Err  z-value  P(>|z|)   Std.lv  Std.all
  ix ~
    age_M00          -0.333    0.044   -7.627    0.000   -0.362   -0.362
    sex               0.033    0.060    0.555    0.579    0.036    0.036
    edyears          -0.058    0.047   -1.237    0.216   -0.063   -0.063
    vsclr_rsk_smcr   -0.035    0.048   -0.737    0.461   -0.039   -0.039
    eTIV             -0.089    0.061   -1.464    0.143   -0.097   -0.097
  sx ~
    age_M00          -0.012    0.008   -1.424    0.154   -0.147   -0.147
    sex               0.004    0.010    0.432    0.666    0.054    0.054
    edyears           0.010    0.008    1.304    0.192    0.124    0.124
    vsclr_rsk_smcr   -0.004    0.009   -0.395    0.693   -0.044   -0.044
    eTIV              0.005    0.010    0.498    0.618    0.064    0.064

Covariances:
                           Estimate  Std.Err  z-value  P(>|z|)   Std.lv  Std.all
 .ix ~~
   .sx                        0.000    0.009    0.056    0.955    0.007    0.007
  age_M00 ~~
    sex                      -0.165    0.046   -3.607    0.000   -0.165   -0.165
    edyears                  -0.119    0.048   -2.478    0.013   -0.119   -0.119
    vsclr_rsk_smcr            0.157    0.047    3.335    0.001    0.157    0.157
    eTIV                      0.057    0.046    1.234    0.217    0.057    0.057
  sex ~~
    edyears                  -0.238    0.043   -5.569    0.000   -0.238   -0.238
    vsclr_rsk_smcr           -0.184    0.045   -4.128    0.000   -0.184   -0.184
    eTIV                     -0.668    0.021  -31.365    0.000   -0.668   -0.668
  edyears ~~
    vsclr_rsk_smcr           -0.156    0.043   -3.625    0.000   -0.156   -0.156
    eTIV                      0.247    0.042    5.956    0.000    0.247    0.247
  vascular_risk_sumcorr ~~
    eTIV                      0.113    0.048    2.362    0.018    0.113    0.113

Intercepts:
                   Estimate  Std.Err  z-value  P(>|z|)   Std.lv  Std.all
   .ix                0.047    0.043    1.077    0.281    0.051    0.051
   .sx               -0.021    0.008   -2.686    0.007   -0.266   -0.266
   .x1                0.000                                0.000    0.000
   .x2                0.000                                0.000    0.000
   .x3                0.000                                0.000    0.000
   .x4                0.000                                0.000    0.000
   .x5                0.000                                0.000    0.000
    age_M00           0.000                                0.000    0.000
    edyears           0.000                                0.000    0.000
```

```
    sex                 0.000                                       0.000    0.000
    vsclr_rsk_smcr      0.000                                       0.000    0.000
    eTIV                0.000                                       0.000    0.000

Variances:
                   Estimate  Std.Err  z-value  P(>|z|)   Std.lv  Std.all
   .ix                0.704    0.060   11.760    0.000    0.836    0.836
   .sx                0.006    0.002    2.430    0.015    0.948    0.948
   .x1      (tht_)    0.131    0.015    8.977    0.000    0.131    0.134
   .x2      (tht_)    0.131    0.015    8.977    0.000    0.131    0.132
   .x3      (tht_)    0.131    0.015    8.977    0.000    0.131    0.129
   .x4      (tht_)    0.131    0.015    8.977    0.000    0.131    0.124
   .x5      (tht_)    0.131    0.015    8.977    0.000    0.131    0.118
    age_M00           1.000                                1.000    1.000
    sex               1.000                                1.000    1.000
    edyears           1.000                                1.000    1.000
    vsclr__           1.000                                1.000    1.000
    eTIV              1.000                                1.000    1.000

R-Square:
                   Estimate
    ix                0.164
    sx                0.052
    x1                0.866
    x2                0.868
    x3                0.871
    x4                0.876
    x5                0.882
```

Standardised solution

```
Latent Variables:
                    est.std  Std.Err  z-value  P(>|z|) ci.lower ci.upper
  ix =~
    x1                0.930    0.009  102.697    0.000    0.913    0.948
    x2                0.923    0.013   69.160    0.000    0.897    0.949
    x3                0.911    0.019   49.081    0.000    0.874    0.947
    x4                0.893    0.023   38.256    0.000    0.847    0.939
    x5                0.871    0.028   31.457    0.000    0.817    0.926
  sx =~
    x1                0.000                                0.000    0.000
    x2                0.080    0.016    4.924    0.000    0.048    0.112
    x3                0.158    0.032    4.913    0.000    0.095    0.221
    x4                0.232    0.047    4.957    0.000    0.140    0.324
```

```
    x5                0.302    0.060    5.050    0.000    0.185    0.419

Regressions:
                   est.std  Std.Err  z-value  P(>|z|) ci.lower ci.upper
  ix ~
    age_M00         -0.362    0.046   -7.889    0.000   -0.452   -0.272
    sex              0.036    0.066    0.555    0.579   -0.092    0.165
    edyears         -0.063    0.051   -1.236    0.216   -0.164    0.037
    vsclr_rsk_smcr  -0.039    0.052   -0.739    0.460   -0.141    0.064
    eTIV            -0.097    0.066   -1.475    0.140   -0.225    0.032
  sx ~
    age_M00         -0.147    0.102   -1.444    0.149   -0.347    0.053
    sex              0.054    0.125    0.433    0.665   -0.192    0.300
    edyears          0.124    0.092    1.343    0.179   -0.057    0.305
    vsclr_rsk_smcr  -0.044    0.112   -0.396    0.692   -0.264    0.175
    eTIV             0.064    0.129    0.493    0.622   -0.189    0.316

Covariances:
                           est.std  Std.Err  z-value  P(>|z|) ci.lower ci.upper
 .ix ~~
   .sx                       0.007    0.134    0.056    0.956   -0.256    0.271
  age_M00 ~~
    sex                     -0.165    0.046   -3.607    0.000   -0.255   -0.075
    edyears                 -0.119    0.048   -2.478    0.013   -0.214   -0.025
    vsclr_rsk_smcr           0.157    0.047    3.335    0.001    0.065    0.249
    eTIV                     0.057    0.046    1.234    0.217   -0.033    0.146
  sex ~~
    edyears                 -0.238    0.043   -5.569    0.000   -0.321   -0.154
    vsclr_rsk_smcr          -0.184    0.045   -4.128    0.000   -0.272   -0.097
    eTIV                    -0.668    0.021  -31.365    0.000   -0.710   -0.626
  edyears ~~
    vsclr_rsk_smcr          -0.156    0.043   -3.625    0.000   -0.241   -0.072
    eTIV                     0.247    0.042    5.956    0.000    0.166    0.329
  vascular_risk_sumcorr ~~
    eTIV                     0.113    0.048    2.362    0.018    0.019    0.207

Intercepts:
                   est.std  Std.Err  z-value  P(>|z|) ci.lower ci.upper
   .ix               0.051    0.047    1.078    0.281   -0.042    0.144
   .sx              -0.266    0.113   -2.353    0.019   -0.488   -0.044
   .x1               0.000                               0.000    0.000
   .x2               0.000                               0.000    0.000
   .x3               0.000                               0.000    0.000
   .x4               0.000                               0.000    0.000
   .x5               0.000                               0.000    0.000
```

age_M00		0.000				0.000	0.000
edyears		0.000				0.000	0.000
sex		0.000				0.000	0.000
vsclr_rsk_smcr		0.000				0.000	0.000
eTIV		0.000				0.000	0.000

Variances:

		est.std	Std.Err	z-value	P(>|z|)	ci.lower	ci.upper
.ix		0.836	0.035	23.572	0.000	0.766	0.905
.sx		0.948	0.042	22.627	0.000	0.866	1.030
.x1	(tht_)	0.134	0.017	7.979	0.000	0.101	0.168
.x2	(tht_)	0.132	0.016	8.223	0.000	0.101	0.164
.x3	(tht_)	0.129	0.016	8.293	0.000	0.098	0.159
.x4	(tht_)	0.124	0.015	8.205	0.000	0.094	0.154
.x5	(tht_)	0.118	0.015	7.977	0.000	0.089	0.147
age_M00		1.000				1.000	1.000
sex		1.000				1.000	1.000
edyears		1.000				1.000	1.000
vsclr__		1.000				1.000	1.000
eTIV		1.000				1.000	1.000

UGLCM – Occipital cortical thickness

Unstandardised solution

```
lavaan 0.6.17 ended normally after 42 iterations

  Estimator                                         ML
  Optimization method                           NLMINB
  Number of model parameters                        30
  Number of equality constraints                     4

  Number of observations                           451
  Number of missing patterns                        27

Model Test User Model:
                                              Standard      Scaled
  Test Statistic                                45.839      36.824
  Degrees of freedom                                39          39
  P-value (Chi-square)                           0.210       0.570
  Scaling correction factor                                  1.245
    Yuan-Bentler correction (Mplus variant)

Model Test Baseline Model:

  Test statistic                              2364.269    1688.949
  Degrees of freedom                                45          45
  P-value                                        0.000       0.000
  Scaling correction factor                                  1.400

User Model versus Baseline Model:

  Comparative Fit Index (CFI)                    0.997       1.000
  Tucker-Lewis Index (TLI)                       0.997       1.002

  Robust Comparative Fit Index (CFI)                         1.000
  Robust Tucker-Lewis Index (TLI)                            1.001

Loglikelihood and Information Criteria:

  Loglikelihood user model (H0)              -4489.394   -4489.394
  Scaling correction factor                                  1.067
      for the MLR correction
  Loglikelihood unrestricted model (H1)      -4466.475   -4466.475
  Scaling correction factor                                  1.239
```

```
    for the MLR correction

  Akaike (AIC)                                9030.789    9030.789
  Bayesian (BIC)                              9137.687    9137.687
  Sample-size adjusted Bayesian (SABIC)       9055.173    9055.173

Root Mean Square Error of Approximation:

  RMSEA                                          0.020       0.000
  90 Percent confidence interval - lower         0.000       0.000
  90 Percent confidence interval - upper         0.040       0.028
  P-value H_0: RMSEA <= 0.050                    0.996       1.000
  P-value H_0: RMSEA >= 0.080                    0.000       0.000

  Robust RMSEA                                               0.000
  90 Percent confidence interval - lower                     0.000
  90 Percent confidence interval - upper                     0.043
  P-value H_0: Robust RMSEA <= 0.050                         0.981
  P-value H_0: Robust RMSEA >= 0.080                         0.000

Standardized Root Mean Square Residual:

  SRMR                                           0.011       0.011

Parameter Estimates:

  Standard errors                             Sandwich
  Information bread                           Observed
  Observed information based on                Hessian

Latent Variables:
                   Estimate  Std.Err  z-value  P(>|z|)   Std.lv  Std.all
  ix =~
    x1                1.000                               0.921    0.928
    x2                1.000                               0.921    0.921
    x3                1.000                               0.921    0.909
    x4                1.000                               0.921    0.892
    x5                1.000                               0.921    0.871
  sx =~
    x1                0.000                               0.000    0.000
    x2                1.000                               0.080    0.080
    x3                2.000                               0.160    0.158
    x4                3.000                               0.241    0.233
    x5                4.000                               0.321    0.303
```

```
Regressions:
                   Estimate  Std.Err  z-value  P(>|z|)   Std.lv  Std.all
  ix ~
    age_M00          -0.233    0.048   -4.870    0.000   -0.253   -0.253
    sex               0.083    0.065    1.285    0.199    0.091    0.091
    edyears           0.021    0.048    0.444    0.657    0.023    0.023
    vsclr_rsk_smcr   -0.026    0.045   -0.574    0.566   -0.028   -0.028
    eTIV              0.001    0.061    0.019    0.985    0.001    0.001
  sx ~
    age_M00          -0.018    0.009   -2.051    0.040   -0.224   -0.224
    sex              -0.005    0.011   -0.464    0.642   -0.063   -0.063
    edyears           0.010    0.008    1.302    0.193    0.127    0.127
    vsclr_rsk_smcr    0.004    0.008    0.506    0.613    0.052    0.052
    eTIV             -0.001    0.012   -0.045    0.964   -0.007   -0.007

Covariances:
                           Estimate  Std.Err  z-value  P(>|z|)   Std.lv  Std.all
 .ix ~~
   .sx                       -0.000    0.008   -0.036    0.971   -0.004   -0.004
  age_M00 ~~
    sex                      -0.165    0.046   -3.607    0.000   -0.165   -0.165
    edyears                  -0.119    0.048   -2.478    0.013   -0.119   -0.119
    vsclr_rsk_smcr            0.157    0.047    3.335    0.001    0.157    0.157
    eTIV                      0.057    0.046    1.234    0.217    0.057    0.057
  sex ~~
    edyears                  -0.238    0.043   -5.569    0.000   -0.238   -0.238
    vsclr_rsk_smcr           -0.184    0.045   -4.128    0.000   -0.184   -0.184
    eTIV                     -0.668    0.021  -31.365    0.000   -0.668   -0.668
  edyears ~~
    vsclr_rsk_smcr           -0.156    0.043   -3.625    0.000   -0.156   -0.156
    eTIV                      0.247    0.042    5.956    0.000    0.247    0.247
  vascular_risk_sumcorr ~~
    eTIV                      0.113    0.048    2.362    0.018    0.113    0.113

Intercepts:
                   Estimate  Std.Err  z-value  P(>|z|)   Std.lv  Std.all
   .ix               -0.018    0.045   -0.391    0.696   -0.019   -0.019
   .sx                0.011    0.008    1.387    0.165    0.139    0.139
   .x1                0.000                                0.000    0.000
   .x2                0.000                                0.000    0.000
   .x3                0.000                                0.000    0.000
   .x4                0.000                                0.000    0.000
   .x5                0.000                                0.000    0.000
    age_M00           0.000                                0.000    0.000
    edyears           0.000                                0.000    0.000
```

```
    sex               0.000                                       0.000    0.000
    vsclr_rsk_smcr    0.000                                       0.000    0.000
    eTIV              0.000                                       0.000    0.000

Variances:
                   Estimate  Std.Err  z-value  P(>|z|)   Std.lv  Std.all
   .ix                0.776    0.057   13.567    0.000    0.916    0.916
   .sx                0.006    0.002    2.749    0.006    0.927    0.927
   .x1      (tht_)    0.137    0.015    9.406    0.000    0.137    0.139
   .x2      (tht_)    0.137    0.015    9.406    0.000    0.137    0.137
   .x3      (tht_)    0.137    0.015    9.406    0.000    0.137    0.134
   .x4      (tht_)    0.137    0.015    9.406    0.000    0.137    0.129
   .x5      (tht_)    0.137    0.015    9.406    0.000    0.137    0.123
    age_M00           1.000                                       1.000    1.000
    sex               1.000                                       1.000    1.000
    edyears           1.000                                       1.000    1.000
    vsclr__           1.000                                       1.000    1.000
    eTIV              1.000                                       1.000    1.000

R-Square:
                   Estimate
    ix                0.084
    sx                0.073
    x1                0.861
    x2                0.863
    x3                0.866
    x4                0.871
    x5                0.877
```

Standardised solution

```
Latent Variables:
                    est.std  Std.Err  z-value  P(>|z|) ci.lower ci.upper
  ix =~
    x1                0.928    0.009  106.180    0.000    0.911    0.945
    x2                0.921    0.012   78.936    0.000    0.899    0.944
    x3                0.909    0.016   56.121    0.000    0.878    0.941
    x4                0.892    0.021   42.881    0.000    0.851    0.933
    x5                0.871    0.025   34.519    0.000    0.822    0.921
  sx =~
    x1                0.000                                0.000    0.000
    x2                0.080    0.014    5.773    0.000    0.053    0.107
    x3                0.158    0.027    5.781    0.000    0.105    0.212
    x4                0.233    0.040    5.852    0.000    0.155    0.311
```

x5	0.303	0.051	5.981	0.000	0.204	0.403

Regressions:

	est.std	Std.Err	z-value	P(>|z|)	ci.lower	ci.upper
ix ~						
age_M00	-0.253	0.051	-4.971	0.000	-0.352	-0.153
sex	0.091	0.070	1.289	0.197	-0.047	0.228
edyears	0.023	0.052	0.444	0.657	-0.079	0.126
vsclr_rsk_smcr	-0.028	0.048	-0.574	0.566	-0.123	0.067
eTIV	0.001	0.066	0.019	0.985	-0.128	0.131
sx ~						
age_M00	-0.224	0.111	-2.015	0.044	-0.441	-0.006
sex	-0.063	0.134	-0.468	0.640	-0.325	0.199
edyears	0.127	0.097	1.305	0.192	-0.064	0.317
vsclr_rsk_smcr	0.052	0.104	0.503	0.615	-0.152	0.257
eTIV	-0.007	0.153	-0.045	0.964	-0.307	0.293

Covariances:

	est.std	Std.Err	z-value	P(>|z|)	ci.lower	ci.upper
.ix ~~						
.sx	-0.004	0.111	-0.036	0.971	-0.221	0.213
age_M00 ~~						
sex	-0.165	0.046	-3.607	0.000	-0.255	-0.075
edyears	-0.119	0.048	-2.478	0.013	-0.214	-0.025
vsclr_rsk_smcr	0.157	0.047	3.335	0.001	0.065	0.249
eTIV	0.057	0.046	1.234	0.217	-0.033	0.146
sex ~~						
edyears	-0.238	0.043	-5.569	0.000	-0.321	-0.154
vsclr_rsk_smcr	-0.184	0.045	-4.128	0.000	-0.272	-0.097
eTIV	-0.668	0.021	-31.365	0.000	-0.710	-0.626
edyears ~~						
vsclr_rsk_smcr	-0.156	0.043	-3.625	0.000	-0.241	-0.072
eTIV	0.247	0.042	5.956	0.000	0.166	0.329
vascular_risk_sumcorr ~~						
eTIV	0.113	0.048	2.362	0.018	0.019	0.207

Intercepts:

	est.std	Std.Err	z-value	P(>|z|)	ci.lower	ci.upper
.ix	-0.019	0.049	-0.390	0.696	-0.116	0.078
.sx	0.139	0.097	1.432	0.152	-0.051	0.330
.x1	0.000				0.000	0.000
.x2	0.000				0.000	0.000
.x3	0.000				0.000	0.000
.x4	0.000				0.000	0.000
.x5	0.000				0.000	0.000

```
    age_M00           0.000                                    0.000    0.000
    edyears           0.000                                    0.000    0.000
    sex               0.000                                    0.000    0.000
    vsclr_rsk_smcr    0.000                                    0.000    0.000
    eTIV              0.000                                    0.000    0.000

Variances:
                   est.std  Std.Err  z-value  P(>|z|) ci.lower ci.upper
   .ix              0.916    0.027   34.131    0.000    0.863    0.968
   .sx              0.927    0.053   17.395    0.000    0.822    1.031
   .x1      (tht_)  0.139    0.016    8.581    0.000    0.107    0.171
   .x2      (tht_)  0.137    0.016    8.671    0.000    0.106    0.168
   .x3      (tht_)  0.134    0.015    8.656    0.000    0.103    0.164
   .x4      (tht_)  0.129    0.015    8.549    0.000    0.099    0.158
   .x5      (tht_)  0.123    0.015    8.356    0.000    0.094    0.151
    age_M00         1.000                                      1.000    1.000
    sex             1.000                                      1.000    1.000
    edyears         1.000                                      1.000    1.000
    vsclr__         1.000                                      1.000    1.000
    eTIV            1.000                                      1.000    1.000
```

ULGCM – Temporal cortical thickness

Unstandardised solution

```
lavaan 0.6.17 ended normally after 43 iterations

  Estimator                                         ML
  Optimization method                           NLMINB
  Number of model parameters                        30
  Number of equality constraints                     4

  Number of observations                           451
  Number of missing patterns                        28

Model Test User Model:
                                              Standard      Scaled
  Test Statistic                                56.062      42.040
  Degrees of freedom                                39          39
  P-value (Chi-square)                           0.038       0.341
  Scaling correction factor                                  1.334
    Yuan-Bentler correction (Mplus variant)

Model Test Baseline Model:

  Test statistic                              2591.223    1699.637
  Degrees of freedom                                45          45
  P-value                                        0.000       0.000
  Scaling correction factor                                  1.525

User Model versus Baseline Model:

  Comparative Fit Index (CFI)                    0.993       0.998
  Tucker-Lewis Index (TLI)                       0.992       0.998

  Robust Comparative Fit Index (CFI)                         0.999
  Robust Tucker-Lewis Index (TLI)                            0.999

Loglikelihood and Information Criteria:

  Loglikelihood user model (H0)              -4330.759   -4330.759
  Scaling correction factor                                  1.147
      for the MLR correction
  Loglikelihood unrestricted model (H1)      -4302.728   -4302.728
  Scaling correction factor                                  1.329
```

```
    for the MLR correction

  Akaike (AIC)                                8713.518    8713.518
  Bayesian (BIC)                              8820.416    8820.416
  Sample-size adjusted Bayesian (SABIC)       8737.901    8737.901

Root Mean Square Error of Approximation:

  RMSEA                                          0.031       0.013
  90 Percent confidence interval - lower         0.008       0.000
  90 Percent confidence interval - upper         0.048       0.034
  P-value H_0: RMSEA <= 0.050                    0.966       1.000
  P-value H_0: RMSEA >= 0.080                    0.000       0.000

  Robust RMSEA                                               0.013
  90 Percent confidence interval - lower                     0.000
  90 Percent confidence interval - upper                     0.051
  P-value H_0: Robust RMSEA <= 0.050                         0.944
  P-value H_0: Robust RMSEA >= 0.080                         0.000

Standardized Root Mean Square Residual:

  SRMR                                           0.019       0.019

Parameter Estimates:

  Standard errors                             Sandwich
  Information bread                           Observed
  Observed information based on                Hessian

Latent Variables:
                   Estimate  Std.Err  z-value  P(>|z|)   Std.lv  Std.all
  ix =~
    x1                1.000                               0.952    0.946
    x2                1.000                               0.952    0.945
    x3                1.000                               0.952    0.934
    x4                1.000                               0.952    0.916
    x5                1.000                               0.952    0.890
  sx =~
    x1                0.000                               0.000    0.000
    x2                1.000                               0.099    0.098
    x3                2.000                               0.197    0.194
    x4                3.000                               0.296    0.285
    x5                4.000                               0.394    0.369
```

```
Regressions:
                   Estimate  Std.Err  z-value  P(>|z|)   Std.lv  Std.all
  ix ~
    age_M00          -0.322    0.046   -7.032    0.000   -0.339   -0.339
    sex              -0.071    0.060   -1.183    0.237   -0.075   -0.075
    edyears          -0.039    0.049   -0.805    0.421   -0.041   -0.041
    vsclr_rsk_smcr   -0.079    0.046   -1.710    0.087   -0.083   -0.083
    eTIV             -0.115    0.062   -1.848    0.065   -0.120   -0.120
  sx ~
    age_M00          -0.024    0.009   -2.828    0.005   -0.248   -0.248
    sex               0.011    0.011    1.026    0.305    0.116    0.116
    edyears           0.012    0.008    1.583    0.113    0.126    0.126
    vsclr_rsk_smcr    0.001    0.008    0.133    0.894    0.011    0.011
    eTIV              0.008    0.011    0.762    0.446    0.082    0.082

Covariances:
                           Estimate  Std.Err  z-value  P(>|z|)   Std.lv  Std.all
 .ix ~~
   .sx                       -0.011    0.010   -1.143    0.253   -0.132   -0.132
  age_M00 ~~
    sex                      -0.165    0.046   -3.607    0.000   -0.165   -0.165
    edyears                  -0.119    0.048   -2.478    0.013   -0.119   -0.119
    vsclr_rsk_smcr            0.157    0.047    3.335    0.001    0.157    0.157
    eTIV                      0.057    0.046    1.234    0.217    0.057    0.057
  sex ~~
    edyears                  -0.238    0.043   -5.569    0.000   -0.238   -0.238
    vsclr_rsk_smcr           -0.184    0.045   -4.128    0.000   -0.184   -0.184
    eTIV                     -0.668    0.021  -31.365    0.000   -0.668   -0.668
  edyears ~~
    vsclr_rsk_smcr           -0.156    0.043   -3.625    0.000   -0.156   -0.156
    eTIV                      0.247    0.042    5.956    0.000    0.247    0.247
  vascular_risk_sumcorr ~~
    eTIV                      0.113    0.048    2.362    0.018    0.113    0.113

Intercepts:
                   Estimate  Std.Err  z-value  P(>|z|)   Std.lv  Std.all
   .ix                0.058    0.045    1.290    0.197    0.061    0.061
   .sx               -0.027    0.008   -3.488    0.000   -0.277   -0.277
   .x1                0.000                                0.000    0.000
   .x2                0.000                                0.000    0.000
   .x3                0.000                                0.000    0.000
   .x4                0.000                                0.000    0.000
   .x5                0.000                                0.000    0.000
    age_M00           0.000                                0.000    0.000
    edyears           0.000                                0.000    0.000
```

```
    sex                0.000                                     0.000    0.000
    vsclr_rsk_smcr     0.000                                     0.000    0.000
    eTIV               0.000                                     0.000    0.000

Variances:
                   Estimate  Std.Err  z-value  P(>|z|)   Std.lv  Std.all
   .ix                0.785    0.064   12.212    0.000    0.867    0.867
   .sx                0.009    0.003    2.874    0.004    0.904    0.904
   .x1      (tht_)    0.105    0.012    9.024    0.000    0.105    0.104
   .x2      (tht_)    0.105    0.012    9.024    0.000    0.105    0.104
   .x3      (tht_)    0.105    0.012    9.024    0.000    0.105    0.102
   .x4      (tht_)    0.105    0.012    9.024    0.000    0.105    0.098
   .x5      (tht_)    0.105    0.012    9.024    0.000    0.105    0.092
    age_M00           1.000                                1.000    1.000
    sex               1.000                                1.000    1.000
    edyears           1.000                                1.000    1.000
    vsclr__           1.000                                1.000    1.000
    eTIV              1.000                                1.000    1.000

R-Square:
                   Estimate
    ix                0.133
    sx                0.096
    x1                0.896
    x2                0.896
    x3                0.898
    x4                0.902
    x5                0.908
```

Standardised solution

```
Latent Variables:
                    est.std  Std.Err  z-value  P(>|z|) ci.lower ci.upper
  ix =~
    x1                0.946    0.007  138.894    0.000    0.933    0.960
    x2                0.945    0.012   79.667    0.000    0.922    0.968
    x3                0.934    0.018   51.137    0.000    0.898    0.970
    x4                0.916    0.024   38.381    0.000    0.869    0.962
    x5                0.890    0.029   31.043    0.000    0.834    0.947
  sx =~
    x1                0.000                                0.000    0.000
    x2                0.098    0.016    6.035    0.000    0.066    0.130
    x3                0.194    0.032    5.995    0.000    0.130    0.257
    x4                0.285    0.047    6.054    0.000    0.192    0.377
```

```
    x5                0.369    0.059    6.205    0.000    0.253    0.486

Regressions:
                   est.std  Std.Err  z-value  P(>|z|) ci.lower ci.upper
  ix ~
    age_M00         -0.339    0.046   -7.295    0.000   -0.430   -0.248
    sex             -0.075    0.063   -1.191    0.234   -0.198    0.048
    edyears         -0.041    0.051   -0.809    0.419   -0.141    0.059
    vsclr_rsk_smcr  -0.083    0.048   -1.727    0.084   -0.177    0.011
    eTIV            -0.120    0.065   -1.861    0.063   -0.247    0.006
  sx ~
    age_M00         -0.248    0.099   -2.513    0.012   -0.441   -0.055
    sex              0.116    0.108    1.082    0.279   -0.094    0.327
    edyears          0.126    0.073    1.715    0.086   -0.018    0.270
    vsclr_rsk_smcr   0.011    0.083    0.132    0.895   -0.151    0.173
    eTIV             0.082    0.105    0.785    0.433   -0.124    0.289

Covariances:
                           est.std  Std.Err  z-value  P(>|z|) ci.lower ci.upper
 .ix ~~
   .sx                      -0.132    0.100   -1.320    0.187   -0.328    0.064
  age_M00 ~~
    sex                     -0.165    0.046   -3.607    0.000   -0.255   -0.075
    edyears                 -0.119    0.048   -2.478    0.013   -0.214   -0.025
    vsclr_rsk_smcr           0.157    0.047    3.335    0.001    0.065    0.249
    eTIV                     0.057    0.046    1.234    0.217   -0.033    0.146
  sex ~~
    edyears                 -0.238    0.043   -5.569    0.000   -0.321   -0.154
    vsclr_rsk_smcr          -0.184    0.045   -4.128    0.000   -0.272   -0.097
    eTIV                    -0.668    0.021  -31.365    0.000   -0.710   -0.626
  edyears ~~
    vsclr_rsk_smcr          -0.156    0.043   -3.625    0.000   -0.241   -0.072
    eTIV                     0.247    0.042    5.956    0.000    0.166    0.329
  vascular_risk_sumcorr ~~
    eTIV                     0.113    0.048    2.362    0.018    0.019    0.207

Intercepts:
                   est.std  Std.Err  z-value  P(>|z|) ci.lower ci.upper
   .ix               0.061    0.047    1.296    0.195   -0.031    0.153
   .sx              -0.277    0.091   -3.025    0.002   -0.456   -0.097
   .x1               0.000                                0.000    0.000
   .x2               0.000                                0.000    0.000
   .x3               0.000                                0.000    0.000
   .x4               0.000                                0.000    0.000
   .x5               0.000                                0.000    0.000
```

```
    age_M00           0.000                                     0.000    0.000
    edyears           0.000                                     0.000    0.000
    sex               0.000                                     0.000    0.000
    vsclr_rsk_smcr    0.000                                     0.000    0.000
    eTIV              0.000                                     0.000    0.000

Variances:
                   est.std  Std.Err  z-value  P(>|z|) ci.lower ci.upper
   .ix               0.867    0.032   27.059    0.000    0.804    0.930
   .sx               0.904    0.047   19.274    0.000    0.812    0.996
   .x1      (tht_)   0.104    0.013    8.077    0.000    0.079    0.129
   .x2      (tht_)   0.104    0.013    8.209    0.000    0.079    0.129
   .x3      (tht_)   0.102    0.012    8.262    0.000    0.077    0.126
   .x4      (tht_)   0.098    0.012    8.266    0.000    0.074    0.121
   .x5      (tht_)   0.092    0.011    8.209    0.000    0.070    0.114
    age_M00          1.000                                1.000    1.000
    sex              1.000                                1.000    1.000
    edyears          1.000                                1.000    1.000
    vsclr__          1.000                                1.000    1.000
    eTIV             1.000                                1.000    1.000
```

ULGCM – Cingulate cortical thickness

Unstandardised solution

```
lavaan 0.6.17 ended normally after 39 iterations

  Estimator                                         ML
  Optimization method                           NLMINB
  Number of model parameters                        30
  Number of equality constraints                     4

  Number of observations                           451
  Number of missing patterns                        30

Model Test User Model:
                                              Standard      Scaled
  Test Statistic                                29.894      25.234
  Degrees of freedom                                39          39
  P-value (Chi-square)                           0.853       0.957
  Scaling correction factor                                  1.185
    Yuan-Bentler correction (Mplus variant)

Model Test Baseline Model:

  Test statistic                              2381.862    1864.781
  Degrees of freedom                                45          45
  P-value                                        0.000       0.000
  Scaling correction factor                                  1.277

User Model versus Baseline Model:

  Comparative Fit Index (CFI)                    1.000       1.000
  Tucker-Lewis Index (TLI)                       1.004       1.009

  Robust Comparative Fit Index (CFI)                         1.000
  Robust Tucker-Lewis Index (TLI)                            1.007

Loglikelihood and Information Criteria:

  Loglikelihood user model (H0)              -4415.219   -4415.219
  Scaling correction factor                                  0.969
      for the MLR correction
  Loglikelihood unrestricted model (H1)      -4400.272   -4400.272
  Scaling correction factor                                  1.158
```

```
      for the MLR correction

  Akaike (AIC)                                8882.438    8882.438
  Bayesian (BIC)                              8989.336    8989.336
  Sample-size adjusted Bayesian (SABIC)       8906.822    8906.822

Root Mean Square Error of Approximation:

  RMSEA                                          0.000       0.000
  90 Percent confidence interval - lower         0.000       0.000
  90 Percent confidence interval - upper         0.019       0.000
  P-value H_0: RMSEA <= 0.050                    1.000       1.000
  P-value H_0: RMSEA >= 0.080                    0.000       0.000

  Robust RMSEA                                               0.000
  90 Percent confidence interval - lower                     0.000
  90 Percent confidence interval - upper                     0.021
  P-value H_0: Robust RMSEA <= 0.050                         0.999
  P-value H_0: Robust RMSEA >= 0.080                         0.000

Standardized Root Mean Square Residual:

  SRMR                                           0.015       0.015

Parameter Estimates:

  Standard errors                             Sandwich
  Information bread                           Observed
  Observed information based on                Hessian

Latent Variables:
                   Estimate  Std.Err  z-value  P(>|z|)   Std.lv  Std.all
  ix =~
    x1                1.000                               0.960    0.943
    x2                1.000                               0.960    0.950
    x3                1.000                               0.960    0.946
    x4                1.000                               0.960    0.930
    x5                1.000                               0.960    0.903
  sx =~
    x1                0.000                               0.000    0.000
    x2                1.000                               0.115    0.114
    x3                2.000                               0.230    0.227
    x4                3.000                               0.345    0.334
    x5                4.000                               0.460    0.433
```

```
Regressions:
                   Estimate  Std.Err  z-value  P(>|z|)   Std.lv  Std.all
  ix ~
    age_M00          -0.212    0.052   -4.089    0.000   -0.221   -0.221
    sex              -0.030    0.060   -0.494    0.621   -0.031   -0.031
    edyears          -0.058    0.050   -1.143    0.253   -0.060   -0.060
    vsclr_rsk_smcr   -0.101    0.046   -2.198    0.028   -0.105   -0.105
    eTIV             -0.090    0.067   -1.347    0.178   -0.093   -0.093
  sx ~
    age_M00          -0.027    0.009   -3.058    0.002   -0.234   -0.234
    sex               0.002    0.011    0.165    0.869    0.016    0.016
    edyears           0.018    0.009    2.093    0.036    0.155    0.155
    vsclr_rsk_smcr   -0.003    0.009   -0.403    0.687   -0.030   -0.030
    eTIV             -0.017    0.012   -1.432    0.152   -0.149   -0.149

Covariances:
                           Estimate  Std.Err  z-value  P(>|z|)   Std.lv  Std.all
 .ix ~~
   .sx                       -0.023    0.009   -2.461    0.014   -0.228   -0.228
  age_M00 ~~
    sex                      -0.165    0.046   -3.607    0.000   -0.165   -0.165
    edyears                  -0.119    0.048   -2.478    0.013   -0.119   -0.119
    vsclr_rsk_smcr            0.157    0.047    3.335    0.001    0.157    0.157
    eTIV                      0.057    0.046    1.234    0.217    0.057    0.057
  sex ~~
    edyears                  -0.238    0.043   -5.569    0.000   -0.238   -0.238
    vsclr_rsk_smcr           -0.184    0.045   -4.128    0.000   -0.184   -0.184
    eTIV                     -0.668    0.021  -31.365    0.000   -0.668   -0.668
  edyears ~~
    vsclr_rsk_smcr           -0.156    0.043   -3.625    0.000   -0.156   -0.156
    eTIV                      0.247    0.042    5.956    0.000    0.247    0.247
  vascular_risk_sumcorr ~~
    eTIV                      0.113    0.048    2.362    0.018    0.113    0.113

Intercepts:
                   Estimate  Std.Err  z-value  P(>|z|)   Std.lv  Std.all
   .ix                0.152    0.047    3.192    0.001    0.158    0.158
   .sx               -0.076    0.009   -8.892    0.000   -0.664   -0.664
   .x1                0.000                                0.000    0.000
   .x2                0.000                                0.000    0.000
   .x3                0.000                                0.000    0.000
   .x4                0.000                                0.000    0.000
   .x5                0.000                                0.000    0.000
    age_M00           0.000                                0.000    0.000
    edyears           0.000                                0.000    0.000
```

```
    sex               0.000                                     0.000    0.000
    vsclr_rsk_smcr    0.000                                     0.000    0.000
    eTIV              0.000                                     0.000    0.000

Variances:
                   Estimate  Std.Err  z-value  P(>|z|)   Std.lv  Std.all
   .ix                0.853    0.064   13.377    0.000    0.926    0.926
   .sx                0.012    0.002    5.292    0.000    0.889    0.889
   .x1      (tht_)    0.116    0.010   11.318    0.000    0.116    0.112
   .x2      (tht_)    0.116    0.010   11.318    0.000    0.116    0.113
   .x3      (tht_)    0.116    0.010   11.318    0.000    0.116    0.112
   .x4      (tht_)    0.116    0.010   11.318    0.000    0.116    0.109
   .x5      (tht_)    0.116    0.010   11.318    0.000    0.116    0.103
    age_M00           1.000                                     1.000    1.000
    sex               1.000                                     1.000    1.000
    edyears           1.000                                     1.000    1.000
    vsclr__           1.000                                     1.000    1.000
    eTIV              1.000                                     1.000    1.000

R-Square:
                   Estimate
    ix                0.074
    sx                0.111
    x1                0.888
    x2                0.887
    x3                0.888
    x4                0.891
    x5                0.897
```

Standardised solution

```
Latent Variables:
                    est.std  Std.Err  z-value  P(>|z|) ci.lower ci.upper
  ix =~
    x1                0.943    0.006  157.391    0.000    0.931    0.954
    x2                0.950    0.010   93.819    0.000    0.930    0.970
    x3                0.946    0.017   55.540    0.000    0.912    0.979
    x4                0.930    0.024   39.466    0.000    0.883    0.976
    x5                0.903    0.029   31.033    0.000    0.846    0.961
  sx =~
    x1                0.000                                0.000    0.000
    x2                0.114    0.011   10.470    0.000    0.093    0.135
    x3                0.227    0.022   10.415    0.000    0.184    0.269
    x4                0.334    0.032   10.551    0.000    0.272    0.396
```

```
    x5                0.433    0.040   10.867    0.000    0.355    0.511

Regressions:
                   est.std  Std.Err  z-value  P(>|z|) ci.lower ci.upper
  ix ~
    age_M00          -0.221    0.052   -4.240    0.000   -0.323   -0.119
    sex              -0.031    0.063   -0.495    0.621   -0.154    0.092
    edyears          -0.060    0.052   -1.146    0.252   -0.163    0.043
    vsclr_rsk_smcr   -0.105    0.047   -2.217    0.027   -0.198   -0.012
    eTIV             -0.093    0.069   -1.350    0.177   -0.229    0.042
  sx ~
    age_M00          -0.234    0.075   -3.114    0.002   -0.381   -0.087
    sex               0.016    0.099    0.166    0.868   -0.178    0.211
    edyears           0.155    0.073    2.130    0.033    0.012    0.297
    vsclr_rsk_smcr   -0.030    0.075   -0.401    0.689   -0.176    0.117
    eTIV             -0.149    0.103   -1.444    0.149   -0.351    0.053

Covariances:
                           est.std  Std.Err  z-value  P(>|z|) ci.lower ci.upper
 .ix ~~
   .sx                       -0.228    0.080   -2.835    0.005   -0.386   -0.070
  age_M00 ~~
    sex                      -0.165    0.046   -3.607    0.000   -0.255   -0.075
    edyears                  -0.119    0.048   -2.478    0.013   -0.214   -0.025
    vsclr_rsk_smcr            0.157    0.047    3.335    0.001    0.065    0.249
    eTIV                      0.057    0.046    1.234    0.217   -0.033    0.146
  sex ~~
    edyears                  -0.238    0.043   -5.569    0.000   -0.321   -0.154
    vsclr_rsk_smcr           -0.184    0.045   -4.128    0.000   -0.272   -0.097
    eTIV                     -0.668    0.021  -31.365    0.000   -0.710   -0.626
  edyears ~~
    vsclr_rsk_smcr           -0.156    0.043   -3.625    0.000   -0.241   -0.072
    eTIV                      0.247    0.042    5.956    0.000    0.166    0.329
  vascular_risk_sumcorr ~~
    eTIV                      0.113    0.048    2.362    0.018    0.019    0.207

Intercepts:
                   est.std  Std.Err  z-value  P(>|z|) ci.lower ci.upper
   .ix                0.158    0.049    3.192    0.001    0.061    0.255
   .sx               -0.664    0.092   -7.198    0.000   -0.845   -0.483
   .x1                0.000                               0.000    0.000
   .x2                0.000                               0.000    0.000
   .x3                0.000                               0.000    0.000
   .x4                0.000                               0.000    0.000
   .x5                0.000                               0.000    0.000
```

age_M00		0.000				0.000	0.000
edyears		0.000				0.000	0.000
sex		0.000				0.000	0.000
vsclr_rsk_smcr		0.000				0.000	0.000
eTIV		0.000				0.000	0.000

Variances:

		est.std	Std.Err	z-value	P(>|z|)	ci.lower	ci.upper
.ix		0.926	0.026	36.022	0.000	0.875	0.976
.sx		0.889	0.044	20.382	0.000	0.803	0.974
.x1	(tht_)	0.112	0.011	9.883	0.000	0.089	0.134
.x2	(tht_)	0.113	0.012	9.725	0.000	0.091	0.136
.x3	(tht_)	0.112	0.012	9.428	0.000	0.089	0.136
.x4	(tht_)	0.109	0.012	9.067	0.000	0.085	0.132
.x5	(tht_)	0.103	0.012	8.688	0.000	0.079	0.126
age_M00		1.000				1.000	1.000
sex		1.000				1.000	1.000
edyears		1.000				1.000	1.000
vsclr__		1.000				1.000	1.000
eTIV		1.000				1.000	1.000

ULGCM – Insular cortical thickness

Unstandardised solution

```
lavaan 0.6.17 ended normally after 41 iterations

  Estimator                                         ML
  Optimization method                           NLMINB
  Number of model parameters                        30
  Number of equality constraints                     4

  Number of observations                           451
  Number of missing patterns                        30

Model Test User Model:
                                              Standard      Scaled
  Test Statistic                                22.438      17.652
  Degrees of freedom                                39          39
  P-value (Chi-square)                           0.984       0.999
  Scaling correction factor                                  1.271
    Yuan-Bentler correction (Mplus variant)

Model Test Baseline Model:

  Test statistic                              2195.918    1564.402
  Degrees of freedom                                45          45
  P-value                                        0.000       0.000
  Scaling correction factor                                  1.404

User Model versus Baseline Model:

  Comparative Fit Index (CFI)                    1.000       1.000
  Tucker-Lewis Index (TLI)                       1.009       1.016

  Robust Comparative Fit Index (CFI)                         1.000
  Robust Tucker-Lewis Index (TLI)                            1.017

Loglikelihood and Information Criteria:

  Loglikelihood user model (H0)              -4513.453   -4513.453
  Scaling correction factor                                  1.049
      for the MLR correction
  Loglikelihood unrestricted model (H1)      -4502.234   -4502.234
  Scaling correction factor                                  1.247
```

```
    for the MLR correction

  Akaike (AIC)                                9078.906    9078.906
  Bayesian (BIC)                              9185.804    9185.804
  Sample-size adjusted Bayesian (SABIC)       9103.289    9103.289

Root Mean Square Error of Approximation:

  RMSEA                                          0.000       0.000
  90 Percent confidence interval - lower         0.000       0.000
  90 Percent confidence interval - upper         0.000       0.000
  P-value H_0: RMSEA <= 0.050                    1.000       1.000
  P-value H_0: RMSEA >= 0.080                    0.000       0.000

  Robust RMSEA                                               0.000
  90 Percent confidence interval - lower                     0.000
  90 Percent confidence interval - upper                     0.000
  P-value H_0: Robust RMSEA <= 0.050                         1.000
  P-value H_0: Robust RMSEA >= 0.080                         0.000

Standardized Root Mean Square Residual:

  SRMR                                           0.014       0.014

Parameter Estimates:

  Standard errors                             Sandwich
  Information bread                           Observed
  Observed information based on                Hessian

Latent Variables:
                   Estimate  Std.Err  z-value  P(>|z|)   Std.lv  Std.all
  ix =~
    x1                1.000                                0.938    0.929
    x2                1.000                                0.938    0.931
    x3                1.000                                0.938    0.924
    x4                1.000                                0.938    0.907
    x5                1.000                                0.938    0.882
  sx =~
    x1                0.000                                0.000    0.000
    x2                1.000                                0.104    0.103
    x3                2.000                                0.208    0.205
    x4                3.000                                0.312    0.301
    x5                4.000                                0.416    0.391
```

```
Regressions:
                   Estimate  Std.Err  z-value  P(>|z|)   Std.lv  Std.all
  ix ~
    age_M00          -0.191    0.050   -3.825    0.000   -0.203   -0.203
    sex              -0.077    0.064   -1.211    0.226   -0.083   -0.083
    edyears          -0.025    0.050   -0.502    0.616   -0.027   -0.027
    vsclr_rsk_smcr   -0.093    0.052   -1.784    0.074   -0.099   -0.099
    eTIV             -0.074    0.069   -1.076    0.282   -0.079   -0.079
  sx ~
    age_M00          -0.016    0.009   -1.767    0.077   -0.155   -0.155
    sex              -0.002    0.012   -0.153    0.878   -0.018   -0.018
    edyears           0.003    0.009    0.363    0.717    0.031    0.031
    vsclr_rsk_smcr    0.000    0.008    0.040    0.968    0.003    0.003
    eTIV             -0.011    0.011   -0.979    0.328   -0.107   -0.107

Covariances:
                           Estimate  Std.Err  z-value  P(>|z|)   Std.lv  Std.all
 .ix ~~
   .sx                       -0.011    0.010   -1.080    0.280   -0.120   -0.120
  age_M00 ~~
    sex                      -0.165    0.046   -3.607    0.000   -0.165   -0.165
    edyears                  -0.119    0.048   -2.478    0.013   -0.119   -0.119
    vsclr_rsk_smcr            0.157    0.047    3.335    0.001    0.157    0.157
    eTIV                      0.057    0.046    1.234    0.217    0.057    0.057
  sex ~~
    edyears                  -0.238    0.043   -5.569    0.000   -0.238   -0.238
    vsclr_rsk_smcr           -0.184    0.045   -4.128    0.000   -0.184   -0.184
    eTIV                     -0.668    0.021  -31.365    0.000   -0.668   -0.668
  edyears ~~
    vsclr_rsk_smcr           -0.156    0.043   -3.625    0.000   -0.156   -0.156
    eTIV                      0.247    0.042    5.956    0.000    0.247    0.247
  vascular_risk_sumcorr ~~
    eTIV                      0.113    0.048    2.362    0.018    0.113    0.113

Intercepts:
                   Estimate  Std.Err  z-value  P(>|z|)   Std.lv  Std.all
   .ix                0.024    0.047    0.518    0.605    0.026    0.026
   .sx               -0.011    0.009   -1.288    0.198   -0.108   -0.108
   .x1                0.000                               0.000    0.000
   .x2                0.000                               0.000    0.000
   .x3                0.000                               0.000    0.000
   .x4                0.000                               0.000    0.000
   .x5                0.000                               0.000    0.000
    age_M00           0.000                               0.000    0.000
    edyears           0.000                               0.000    0.000
```

```
    sex                0.000                                      0.000    0.000
    vsclr_rsk_smcr     0.000                                      0.000    0.000
    eTIV               0.000                                      0.000    0.000

Variances:
                   Estimate  Std.Err  z-value  P(>|z|)   Std.lv  Std.all
   .ix                0.831    0.063   13.202    0.000    0.945    0.945
   .sx                0.010    0.004    2.580    0.010    0.965    0.965
   .x1      (tht_)    0.139    0.012   11.837    0.000    0.139    0.136
   .x2      (tht_)    0.139    0.012   11.837    0.000    0.139    0.137
   .x3      (tht_)    0.139    0.012   11.837    0.000    0.139    0.134
   .x4      (tht_)    0.139    0.012   11.837    0.000    0.139    0.129
   .x5      (tht_)    0.139    0.012   11.837    0.000    0.139    0.123
    age_M00           1.000                                1.000    1.000
    sex               1.000                                1.000    1.000
    edyears           1.000                                1.000    1.000
    vsclr__           1.000                                1.000    1.000
    eTIV              1.000                                1.000    1.000

R-Square:
                   Estimate
    ix                0.055
    sx                0.035
    x1                0.864
    x2                0.863
    x3                0.866
    x4                0.871
    x5                0.877
```

Standardised solution

```
Latent Variables:
                    est.std  Std.Err  z-value  P(>|z|) ci.lower ci.upper
  ix =~
    x1                0.929    0.007  128.361    0.000    0.915    0.944
    x2                0.931    0.012   80.637    0.000    0.909    0.954
    x3                0.924    0.017   53.502    0.000    0.890    0.957
    x4                0.907    0.023   39.969    0.000    0.862    0.951
    x5                0.882    0.028   31.248    0.000    0.827    0.937
  sx =~
    x1                0.000                               0.000    0.000
    x2                0.103    0.020    5.165    0.000    0.064    0.142
    x3                0.205    0.040    5.167    0.000    0.127    0.282
    x4                0.301    0.057    5.267    0.000    0.189    0.413
```

```
    x5                0.391    0.072    5.461    0.000    0.250    0.531

Regressions:
                   est.std  Std.Err  z-value  P(>|z|) ci.lower ci.upper
  ix ~
    age_M00         -0.203    0.052   -3.907    0.000   -0.306   -0.101
    sex             -0.083    0.068   -1.212    0.225   -0.216    0.051
    edyears         -0.027    0.053   -0.502    0.615   -0.130    0.077
    vsclr_rsk_smcr  -0.099    0.055   -1.793    0.073   -0.207    0.009
    eTIV            -0.079    0.073   -1.077    0.281   -0.222    0.065
  sx ~
    age_M00         -0.155    0.086   -1.815    0.069   -0.323    0.012
    sex             -0.018    0.114   -0.154    0.877   -0.242    0.207
    edyears          0.031    0.084    0.370    0.711   -0.133    0.195
    vsclr_rsk_smcr   0.003    0.079    0.040    0.968   -0.153    0.159
    eTIV            -0.107    0.109   -0.983    0.326   -0.321    0.106

Covariances:
                           est.std  Std.Err  z-value  P(>|z|) ci.lower ci.upper
 .ix ~~
   .sx                      -0.120    0.096   -1.249    0.212   -0.307    0.068
  age_M00 ~~
    sex                     -0.165    0.046   -3.607    0.000   -0.255   -0.075
    edyears                 -0.119    0.048   -2.478    0.013   -0.214   -0.025
    vsclr_rsk_smcr           0.157    0.047    3.335    0.001    0.065    0.249
    eTIV                     0.057    0.046    1.234    0.217   -0.033    0.146
  sex ~~
    edyears                 -0.238    0.043   -5.569    0.000   -0.321   -0.154
    vsclr_rsk_smcr          -0.184    0.045   -4.128    0.000   -0.272   -0.097
    eTIV                    -0.668    0.021  -31.365    0.000   -0.710   -0.626
  edyears ~~
    vsclr_rsk_smcr          -0.156    0.043   -3.625    0.000   -0.241   -0.072
    eTIV                     0.247    0.042    5.956    0.000    0.166    0.329
  vascular_risk_sumcorr ~~
    eTIV                     0.113    0.048    2.362    0.018    0.019    0.207

Intercepts:
                   est.std  Std.Err  z-value  P(>|z|) ci.lower ci.upper
   .ix               0.026    0.050    0.517    0.605   -0.072    0.124
   .sx              -0.108    0.088   -1.234    0.217   -0.280    0.064
   .x1               0.000                                0.000    0.000
   .x2               0.000                                0.000    0.000
   .x3               0.000                                0.000    0.000
   .x4               0.000                                0.000    0.000
   .x5               0.000                                0.000    0.000
```

```
    age_M00          0.000                                    0.000    0.000
    edyears          0.000                                    0.000    0.000
    sex              0.000                                    0.000    0.000
    vsclr_rsk_smcr   0.000                                    0.000    0.000
    eTIV             0.000                                    0.000    0.000

Variances:
                   est.std  Std.Err  z-value  P(>|z|) ci.lower ci.upper
   .ix               0.945    0.023   41.532    0.000    0.900    0.989
   .sx               0.965    0.031   30.938    0.000    0.904    1.026
   .x1      (tht_)   0.136    0.013   10.109    0.000    0.110    0.162
   .x2      (tht_)   0.137    0.013   10.199    0.000    0.110    0.163
   .x3      (tht_)   0.134    0.013   10.122    0.000    0.108    0.160
   .x4      (tht_)   0.129    0.013    9.910    0.000    0.104    0.155
   .x5      (tht_)   0.123    0.013    9.497    0.000    0.097    0.148
    age_M00          1.000                                    1.000    1.000
    sex              1.000                                    1.000    1.000
    edyears          1.000                                    1.000    1.000
    vsclr__          1.000                                    1.000    1.000
    eTIV             1.000                                    1.000    1.000
```

BLGCM - Total WMH volumes vs mean cortical thickness

Unstandardised solution

```
lavaan 0.6.17 ended normally after 102 iterations

  Estimator                                         ML
  Optimization method                           NLMINB
  Number of model parameters                        59
  Number of equality constraints                    12

  Number of observations                           451
  Number of missing patterns                        78

Model Test User Model:
                                              Standard      Scaled
  Test Statistic                               123.722     101.530
  Degrees of freedom                                88          88
  P-value (Chi-square)                           0.007       0.154
  Scaling correction factor                                  1.219
    Yuan-Bentler correction (Mplus variant)

Model Test Baseline Model:

  Test statistic                              7114.266    5385.354
  Degrees of freedom                               105         105
  P-value                                        0.000       0.000
  Scaling correction factor                                  1.321

User Model versus Baseline Model:

  Comparative Fit Index (CFI)                    0.995       0.997
  Tucker-Lewis Index (TLI)                       0.994       0.997

  Robust Comparative Fit Index (CFI)                         0.998
  Robust Tucker-Lewis Index (TLI)                            0.997

Loglikelihood and Information Criteria:

  Loglikelihood user model (H0)              -4645.374   -4645.374
  Scaling correction factor                                  0.987
      for the MLR correction
  Loglikelihood unrestricted model (H1)      -4583.514   -4583.514
  Scaling correction factor                                  1.226
```

```
     for the MLR correction

  Akaike (AIC)                                9384.749    9384.749
  Bayesian (BIC)                              9577.988    9577.988
  Sample-size adjusted Bayesian (SABIC)       9428.827    9428.827

Root Mean Square Error of Approximation:

  RMSEA                                          0.030       0.018
  90 Percent confidence interval - lower         0.016       0.000
  90 Percent confidence interval - upper         0.042       0.032
  P-value H_0: RMSEA <= 0.050                    0.998       1.000
  P-value H_0: RMSEA >= 0.080                    0.000       0.000

  Robust RMSEA                                               0.024
  90 Percent confidence interval - lower                     0.000
  90 Percent confidence interval - upper                     0.046
  P-value H_0: Robust RMSEA <= 0.050                         0.980
  P-value H_0: Robust RMSEA >= 0.080                         0.000

Standardized Root Mean Square Residual:

  SRMR                                           0.015       0.015

Parameter Estimates:

  Standard errors                             Sandwich
  Information bread                           Observed
  Observed information based on                Hessian

Latent Variables:
                   Estimate  Std.Err  z-value  P(>|z|)   Std.lv  Std.all
  iy =~
    y1                1.000                               0.998    0.991
    y2                1.000                               0.998    0.990
    y3                1.000                               0.998    0.984
    y4                1.000                               0.998    0.975
    y5                1.000                               0.998    0.963
  sy =~
    y1                0.000                               0.000    0.000
    y2                1.000                               0.063    0.063
    y3                2.000                               0.127    0.125
    y4                3.000                               0.190    0.185
    y5                4.000                               0.253    0.244
  ix =~
```

```
    x1                1.000                                       0.928    0.939
    x2                1.000                                       0.928    0.928
    x3                1.000                                       0.928    0.909
    x4                1.000                                       0.928    0.885
    x5                1.000                                       0.928    0.857
  sx =~
    x1                0.000                                       0.000    0.000
    x2                1.000                                       0.091    0.091
    x3                2.000                                       0.182    0.179
    x4                3.000                                       0.273    0.261
    x5                4.000                                       0.364    0.336

Regressions:
                   Estimate  Std.Err  z-value  P(>|z|)   Std.lv  Std.all
  iy ~
    age_M00           0.373    0.044    8.441    0.000    0.374    0.374
    sex               0.188    0.060    3.126    0.002    0.189    0.189
    edyears          -0.027    0.044   -0.619    0.536   -0.027   -0.027
    vsclr_rsk_smcr    0.102    0.045    2.275    0.023    0.102    0.102
    eTIV              0.259    0.056    4.633    0.000    0.260    0.260
  sy ~
    age_M00          -0.005    0.004   -1.118    0.263   -0.078   -0.078
    sex               0.000    0.006    0.090    0.929    0.008    0.008
    edyears          -0.004    0.004   -1.063    0.288   -0.070   -0.070
    vsclr_rsk_smcr   -0.001    0.004   -0.320    0.749   -0.021   -0.021
    eTIV             -0.002    0.005   -0.322    0.747   -0.024   -0.024
  ix ~
    age_M00          -0.311    0.046   -6.750    0.000   -0.336   -0.336
    sex              -0.034    0.059   -0.577    0.564   -0.037   -0.037
    edyears          -0.044    0.047   -0.936    0.349   -0.048   -0.048
    vsclr_rsk_smcr   -0.072    0.047   -1.510    0.131   -0.077   -0.077
    eTIV             -0.116    0.061   -1.899    0.058   -0.125   -0.125
  sx ~
    age_M00          -0.025    0.008   -3.114    0.002   -0.279   -0.279
    sex               0.009    0.010    0.911    0.362    0.103    0.103
    edyears           0.009    0.007    1.238    0.216    0.096    0.096
    vsclr_rsk_smcr    0.005    0.008    0.623    0.533    0.053    0.053
    eTIV              0.002    0.010    0.244    0.807    0.027    0.027

Covariances:
                           Estimate  Std.Err  z-value  P(>|z|)   Std.lv  Std.all
 .iy ~~
   .sy                        0.002    0.004    0.393    0.695    0.028    0.028
 .ix ~~
   .sx                       -0.000    0.009   -0.024    0.981   -0.003   -0.003
```

```
  .y1 ~~
    .x1      (tht_)         -0.002    0.002   -1.085    0.278   -0.002   -0.037
  .y2 ~~
    .x2      (tht_)         -0.002    0.002   -1.085    0.278   -0.002   -0.037
  .y3 ~~
    .x3      (tht_)         -0.002    0.002   -1.085    0.278   -0.002   -0.037
  .y4 ~~
    .x4      (tht_)         -0.002    0.002   -1.085    0.278   -0.002   -0.037
  .y5 ~~
    .x5      (tht_)         -0.002    0.002   -1.085    0.278   -0.002   -0.037
  .iy ~~
    .ix                     -0.128    0.038   -3.400    0.001   -0.165   -0.165
    .sx                     -0.018    0.007   -2.413    0.016   -0.226   -0.226
  .sy ~~
    .ix                     -0.008    0.003   -2.190    0.029   -0.141   -0.141
    .sx                     -0.001    0.001   -1.733    0.083   -0.239   -0.239
   age_M00 ~~
     sex                    -0.165    0.046   -3.607    0.000   -0.165   -0.165
     edyears                -0.119    0.048   -2.478    0.013   -0.119   -0.119
     vsclr__                 0.157    0.047    3.335    0.001    0.157    0.157
     eTIV                    0.057    0.046    1.234    0.217    0.057    0.057
   sex ~~
     edyears                -0.238    0.043   -5.569    0.000   -0.238   -0.238
     vsclr__                -0.184    0.045   -4.128    0.000   -0.184   -0.184
     eTIV                   -0.668    0.021  -31.365    0.000   -0.668   -0.668
   edyears ~~
     vsclr__                -0.156    0.043   -3.625    0.000   -0.156   -0.156
     eTIV                    0.247    0.042    5.956    0.000    0.247    0.247
   vascular_risk_sumcorr ~~
     eTIV                    0.113    0.048    2.362    0.018    0.113    0.113

Intercepts:
                   Estimate  Std.Err  z-value  P(>|z|)   Std.lv  Std.all
    .iy              -0.131    0.043   -3.066    0.002   -0.131   -0.131
    .sy               0.070    0.004   17.654    0.000    1.112    1.112
    .y1               0.000                                0.000    0.000
    .y2               0.000                                0.000    0.000
    .y3               0.000                                0.000    0.000
    .y4               0.000                                0.000    0.000
    .y5               0.000                                0.000    0.000
    .ix               0.027    0.044    0.607    0.544    0.029    0.029
    .sx              -0.020    0.008   -2.524    0.012   -0.216   -0.216
    .x1               0.000                                0.000    0.000
    .x2               0.000                                0.000    0.000
    .x3               0.000                                0.000    0.000
```

```
   .x4                0.000                                     0.000    0.000
   .x5                0.000                                     0.000    0.000
    age_M00           0.000                                     0.000    0.000
    edyears           0.000                                     0.000    0.000
    sex               0.000                                     0.000    0.000
    vsclr_rsk_smcr    0.000                                     0.000    0.000
    eTIV              0.000                                     0.000    0.000

Variances:
                   Estimate  Std.Err  z-value  P(>|z|)   Std.lv  Std.all
   .iy                0.808    0.049   16.410    0.000    0.811    0.811
   .sy                0.004    0.001    4.935    0.000    0.987    0.987
   .y1      (tht_y)   0.018    0.002   10.506    0.000    0.018    0.017
   .y2      (tht_y)   0.018    0.002   10.506    0.000    0.018    0.017
   .y3      (tht_y)   0.018    0.002   10.506    0.000    0.018    0.017
   .y4      (tht_y)   0.018    0.002   10.506    0.000    0.018    0.017
   .y5      (tht_y)   0.018    0.002   10.506    0.000    0.018    0.016
   .ix                0.741    0.062   11.937    0.000    0.861    0.861
   .sx                0.007    0.003    2.832    0.005    0.899    0.899
   .x1      (tht_x)   0.115    0.011   10.396    0.000    0.115    0.118
   .x2      (tht_x)   0.115    0.011   10.396    0.000    0.115    0.115
   .x3      (tht_x)   0.115    0.011   10.396    0.000    0.115    0.111
   .x4      (tht_x)   0.115    0.011   10.396    0.000    0.115    0.105
   .x5      (tht_x)   0.115    0.011   10.396    0.000    0.115    0.098
    ag_M00            1.000                                     1.000    1.000
    sex               1.000                                     1.000    1.000
    edyers            1.000                                     1.000    1.000
    vscl__            1.000                                     1.000    1.000
    eTIV              1.000                                     1.000    1.000

R-Square:
                   Estimate
    iy                0.189
    sy                0.013
    y1                0.983
    y2                0.983
    y3                0.983
    y4                0.983
    y5                0.984
    ix                0.139
    sx                0.101
    x1                0.882
    x2                0.885
    x3                0.889
    x4                0.895
```

```
    x5                 0.902
```

Standardised solution

```
Latent Variables:
                    est.std  Std.Err  z-value  P(>|z|) ci.lower ci.upper
  iy =~
    y1                0.991    0.001 1077.640    0.000    0.990    0.993
    y2                0.990    0.005  215.849    0.000    0.981    0.999
    y3                0.984    0.009  115.480    0.000    0.968    1.001
    y4                0.975    0.012   80.332    0.000    0.952    0.999
    y5                0.963    0.015   62.428    0.000    0.933    0.993
  sy =~
    y1                0.000                               0.000    0.000
    y2                0.063    0.007    9.607    0.000    0.050    0.076
    y3                0.125    0.013    9.547    0.000    0.099    0.150
    y4                0.185    0.019    9.548    0.000    0.147    0.224
    y5                0.244    0.025    9.608    0.000    0.194    0.294
  ix =~
    x1                0.939    0.007  132.378    0.000    0.925    0.953
    x2                0.928    0.012   80.214    0.000    0.905    0.950
    x3                0.909    0.018   51.752    0.000    0.875    0.944
    x4                0.885    0.023   38.257    0.000    0.840    0.931
    x5                0.857    0.028   30.410    0.000    0.802    0.912
  sx =~
    x1                0.000                               0.000    0.000
    x2                0.091    0.016    5.782    0.000    0.060    0.122
    x3                0.179    0.031    5.803    0.000    0.118    0.239
    x4                0.261    0.044    5.901    0.000    0.174    0.347
    x5                0.336    0.055    6.069    0.000    0.228    0.445

Regressions:
                    est.std  Std.Err  z-value  P(>|z|) ci.lower ci.upper
  iy ~
    age_M00           0.374    0.042    8.934    0.000    0.292    0.456
    sex               0.189    0.060    3.158    0.002    0.072    0.306
    edyears          -0.027    0.044   -0.620    0.536   -0.114    0.059
    vsclr_rsk_smcr    0.102    0.044    2.307    0.021    0.015    0.188
    eTIV              0.260    0.054    4.779    0.000    0.153    0.366
  sy ~
    age_M00          -0.078    0.069   -1.128    0.259   -0.212    0.057
    sex               0.008    0.088    0.089    0.929   -0.165    0.181
    edyears          -0.070    0.067   -1.053    0.292   -0.202    0.061
    vsclr_rsk_smcr   -0.021    0.066   -0.321    0.748   -0.151    0.109
```

```
    eTIV              -0.024    0.075   -0.325    0.745   -0.172    0.123
  ix ~
    age_M00           -0.336    0.048   -7.061    0.000   -0.429   -0.243
    sex               -0.037    0.064   -0.578    0.563   -0.162    0.088
    edyears           -0.048    0.051   -0.943    0.346   -0.147    0.051
    vsclr_rsk_smcr    -0.077    0.051   -1.522    0.128   -0.176    0.022
    eTIV              -0.125    0.066   -1.912    0.056   -0.254    0.003
  sx ~
    age_M00           -0.279    0.083   -3.384    0.001   -0.441   -0.118
    sex                0.103    0.110    0.934    0.350   -0.113    0.319
    edyears            0.096    0.077    1.241    0.215   -0.055    0.246
    vsclr_rsk_smcr     0.053    0.087    0.610    0.542   -0.118    0.224
    eTIV               0.027    0.112    0.245    0.807   -0.191    0.246

Covariances:
                          est.std  Std.Err  z-value  P(>|z|) ci.lower ci.upper
 .iy ~~
   .sy                      0.028    0.072    0.386    0.699   -0.113    0.169
 .ix ~~
   .sx                     -0.003    0.120   -0.024    0.981   -0.238    0.232
 .y1 ~~
   .x1      (tht_)         -0.037    0.034   -1.086    0.278   -0.103    0.030
 .y2 ~~
   .x2      (tht_)         -0.037    0.034   -1.086    0.278   -0.103    0.030
 .y3 ~~
   .x3      (tht_)         -0.037    0.034   -1.086    0.278   -0.103    0.030
 .y4 ~~
   .x4      (tht_)         -0.037    0.034   -1.086    0.278   -0.103    0.030
 .y5 ~~
   .x5      (tht_)         -0.037    0.034   -1.086    0.278   -0.103    0.030
 .iy ~~
   .ix                     -0.165    0.047   -3.515    0.000   -0.257   -0.073
   .sx                     -0.226    0.093   -2.443    0.015   -0.408   -0.045
 .sy ~~
   .ix                     -0.141    0.060   -2.336    0.020   -0.259   -0.023
   .sx                     -0.239    0.139   -1.710    0.087   -0.512    0.035
  age_M00 ~~
    sex                    -0.165    0.046   -3.607    0.000   -0.255   -0.075
    edyears                -0.119    0.048   -2.478    0.013   -0.214   -0.025
    vsclr__                 0.157    0.047    3.335    0.001    0.065    0.249
    eTIV                    0.057    0.046    1.234    0.217   -0.033    0.146
  sex ~~
    edyears                -0.238    0.043   -5.569    0.000   -0.321   -0.154
    vsclr__                -0.184    0.045   -4.128    0.000   -0.272   -0.097
    eTIV                   -0.668    0.021  -31.365    0.000   -0.710   -0.626
```

```
  edyears ~~
    vsclr__                -0.156    0.043   -3.625    0.000   -0.241   -0.072
    eTIV                    0.247    0.042    5.956    0.000    0.166    0.329
  vascular_risk_sumcorr ~~
    eTIV                    0.113    0.048    2.362    0.018    0.019    0.207

Intercepts:
                   est.std  Std.Err  z-value  P(>|z|) ci.lower ci.upper
   .iy              -0.131    0.043   -3.045    0.002   -0.215   -0.047
   .sy               1.112    0.109   10.187    0.000    0.898    1.327
   .y1               0.000                               0.000    0.000
   .y2               0.000                               0.000    0.000
   .y3               0.000                               0.000    0.000
   .y4               0.000                               0.000    0.000
   .y5               0.000                               0.000    0.000
   .ix               0.029    0.047    0.607    0.544   -0.064    0.122
   .sx              -0.216    0.096   -2.238    0.025   -0.404   -0.027
   .x1               0.000                               0.000    0.000
   .x2               0.000                               0.000    0.000
   .x3               0.000                               0.000    0.000
   .x4               0.000                               0.000    0.000
   .x5               0.000                               0.000    0.000
    age_M00          0.000                               0.000    0.000
    edyears          0.000                               0.000    0.000
    sex              0.000                               0.000    0.000
    vsclr_rsk_smcr   0.000                               0.000    0.000
    eTIV             0.000                               0.000    0.000

Variances:
                   est.std  Std.Err  z-value  P(>|z|) ci.lower ci.upper
   .iy               0.811    0.032   25.314    0.000    0.748    0.874
   .sy               0.987    0.015   67.574    0.000    0.959    1.016
   .y1     (tht_y)   0.017    0.002    9.481    0.000    0.014    0.021
   .y2     (tht_y)   0.017    0.002    9.612    0.000    0.014    0.021
   .y3     (tht_y)   0.017    0.002    9.685    0.000    0.014    0.021
   .y4     (tht_y)   0.017    0.002    9.704    0.000    0.013    0.020
   .y5     (tht_y)   0.016    0.002    9.672    0.000    0.013    0.020
   .ix               0.861    0.034   25.671    0.000    0.795    0.927
   .sx               0.899    0.051   17.466    0.000    0.798    1.000
   .x1     (tht_x)   0.118    0.013    8.867    0.000    0.092    0.144
   .x2     (tht_x)   0.115    0.013    9.032    0.000    0.090    0.140
   .x3     (tht_x)   0.111    0.012    9.100    0.000    0.087    0.135
   .x4     (tht_x)   0.105    0.012    9.080    0.000    0.082    0.128
   .x5     (tht_x)   0.098    0.011    8.954    0.000    0.077    0.120
    ag_M00           1.000                               1.000    1.000
```

sex	1.000	1.000	1.000
edyers	1.000	1.000	1.000
vscl__	1.000	1.000	1.000
eTIV	1.000	1.000	1.000

BLGCM - Total WMH volumes vs frontal cortical thickness

Unstandardised solution

```
lavaan 0.6.17 ended normally after 99 iterations

  Estimator                                         ML
  Optimization method                           NLMINB
  Number of model parameters                        59
  Number of equality constraints                    12

  Number of observations                           451
  Number of missing patterns                        77

Model Test User Model:
                                              Standard      Scaled
  Test Statistic                               107.531      90.972
  Degrees of freedom                                88          88
  P-value (Chi-square)                           0.077       0.393
  Scaling correction factor                                  1.182
    Yuan-Bentler correction (Mplus variant)

Model Test Baseline Model:

  Test statistic                              7026.565    5515.681
  Degrees of freedom                               105         105
  P-value                                        0.000       0.000
  Scaling correction factor                                  1.274

User Model versus Baseline Model:

  Comparative Fit Index (CFI)                    0.997       0.999
  Tucker-Lewis Index (TLI)                       0.997       0.999

  Robust Comparative Fit Index (CFI)                         1.000
  Robust Tucker-Lewis Index (TLI)                            1.000

Loglikelihood and Information Criteria:

  Loglikelihood user model (H0)              -4688.907   -4688.907
  Scaling correction factor                                  0.953
      for the MLR correction
  Loglikelihood unrestricted model (H1)      -4635.141   -4635.141
  Scaling correction factor                                  1.187
```

```
      for the MLR correction

  Akaike (AIC)                                9471.814    9471.814
  Bayesian (BIC)                              9665.053    9665.053
  Sample-size adjusted Bayesian (SABIC)       9515.892    9515.892

Root Mean Square Error of Approximation:

  RMSEA                                          0.022       0.009
  90 Percent confidence interval - lower         0.000       0.000
  90 Percent confidence interval - upper         0.035       0.026
  P-value H_0: RMSEA <= 0.050                    1.000       1.000
  P-value H_0: RMSEA >= 0.080                    0.000       0.000

  Robust RMSEA                                               0.000
  90 Percent confidence interval - lower                     0.000
  90 Percent confidence interval - upper                     0.036
  P-value H_0: Robust RMSEA <= 0.050                         0.998
  P-value H_0: Robust RMSEA >= 0.080                         0.000

Standardized Root Mean Square Residual:

  SRMR                                           0.015       0.015

Parameter Estimates:

  Standard errors                             Sandwich
  Information bread                           Observed
  Observed information based on                Hessian

Latent Variables:
                   Estimate  Std.Err  z-value  P(>|z|)   Std.lv  Std.all
  iy =~
    y1                1.000                               0.999    0.991
    y2                1.000                               0.999    0.990
    y3                1.000                               0.999    0.985
    y4                1.000                               0.999    0.976
    y5                1.000                               0.999    0.963
  sy =~
    y1                0.000                               0.000    0.000
    y2                1.000                               0.063    0.063
    y3                2.000                               0.127    0.125
    y4                3.000                               0.190    0.185
    y5                4.000                               0.253    0.244
  ix =~
```

```
    x1                1.000                               0.925    0.936
    x2                1.000                               0.925    0.925
    x3                1.000                               0.925    0.907
    x4                1.000                               0.925    0.882
    x5                1.000                               0.925    0.853
  sx =~
    x1                0.000                               0.000    0.000
    x2                1.000                               0.094    0.094
    x3                2.000                               0.188    0.185
    x4                3.000                               0.283    0.270
    x5                4.000                               0.377    0.348

Regressions:
                   Estimate  Std.Err  z-value  P(>|z|)   Std.lv  Std.all
  iy ~
    age_M00           0.373    0.044    8.441    0.000    0.374    0.374
    sex               0.188    0.060    3.124    0.002    0.189    0.189
    edyears          -0.027    0.044   -0.622    0.534   -0.027   -0.027
    vsclr_rsk_smcr    0.102    0.045    2.275    0.023    0.102    0.102
    eTIV              0.259    0.056    4.633    0.000    0.260    0.260
  sy ~
    age_M00          -0.005    0.004   -1.141    0.254   -0.079   -0.079
    sex               0.001    0.006    0.109    0.913    0.010    0.010
    edyears          -0.004    0.004   -1.041    0.298   -0.069   -0.069
    vsclr_rsk_smcr   -0.001    0.004   -0.316    0.752   -0.021   -0.021
    eTIV             -0.002    0.005   -0.328    0.743   -0.025   -0.025
  ix ~
    age_M00          -0.326    0.045   -7.311    0.000   -0.352   -0.352
    sex              -0.034    0.061   -0.564    0.573   -0.037   -0.037
    edyears          -0.071    0.047   -1.493    0.135   -0.076   -0.076
    vsclr_rsk_smcr   -0.079    0.048   -1.655    0.098   -0.085   -0.085
    eTIV             -0.132    0.060   -2.190    0.029   -0.143   -0.143
  sx ~
    age_M00          -0.014    0.008   -1.621    0.105   -0.143   -0.143
    sex               0.009    0.010    0.855    0.392    0.093    0.093
    edyears           0.008    0.008    0.946    0.344    0.080    0.080
    vsclr_rsk_smcr    0.006    0.009    0.709    0.478    0.064    0.064
    eTIV              0.005    0.010    0.476    0.634    0.051    0.051

Covariances:
                           Estimate  Std.Err  z-value  P(>|z|)   Std.lv  Std.all
 .iy ~~
   .sy                        0.002    0.004    0.386    0.700    0.027    0.027
 .ix ~~
   .sx                        0.003    0.009    0.362    0.717    0.043    0.043
```

```
 .y1 ~~
   .x1      (tht_)          -0.002    0.002   -1.182    0.237   -0.002   -0.040
 .y2 ~~
   .x2      (tht_)          -0.002    0.002   -1.182    0.237   -0.002   -0.040
 .y3 ~~
   .x3      (tht_)          -0.002    0.002   -1.182    0.237   -0.002   -0.040
 .y4 ~~
   .x4      (tht_)          -0.002    0.002   -1.182    0.237   -0.002   -0.040
 .y5 ~~
   .x5      (tht_)          -0.002    0.002   -1.182    0.237   -0.002   -0.040
 .iy ~~
   .ix                      -0.096    0.039   -2.479    0.013   -0.126   -0.126
   .sx                      -0.010    0.008   -1.372    0.170   -0.124   -0.124
 .sy ~~
   .ix                      -0.005    0.003   -1.348    0.178   -0.088   -0.088
   .sx                      -0.002    0.001   -1.908    0.056   -0.261   -0.261
  age_M00 ~~
    sex                     -0.165    0.046   -3.607    0.000   -0.165   -0.165
    edyears                 -0.119    0.048   -2.478    0.013   -0.119   -0.119
    vsclr__                  0.157    0.047    3.335    0.001    0.157    0.157
    eTIV                     0.057    0.046    1.234    0.217    0.057    0.057
  sex ~~
    edyears                 -0.238    0.043   -5.569    0.000   -0.238   -0.238
    vsclr__                 -0.184    0.045   -4.128    0.000   -0.184   -0.184
    eTIV                    -0.668    0.021  -31.365    0.000   -0.668   -0.668
  edyears ~~
    vsclr__                 -0.156    0.043   -3.625    0.000   -0.156   -0.156
    eTIV                     0.247    0.042    5.956    0.000    0.247    0.247
  vascular_risk_sumcorr ~~
    eTIV                     0.113    0.048    2.362    0.018    0.113    0.113

Intercepts:
                   Estimate  Std.Err  z-value  P(>|z|)   Std.lv  Std.all
   .iy               -0.131    0.043   -3.066    0.002   -0.131   -0.131
   .sy                0.070    0.004   17.662    0.000    1.112    1.112
   .y1                0.000                                0.000    0.000
   .y2                0.000                                0.000    0.000
   .y3                0.000                                0.000    0.000
   .y4                0.000                                0.000    0.000
   .y5                0.000                                0.000    0.000
   .ix               -0.006    0.044   -0.147    0.883   -0.007   -0.007
   .sx                0.003    0.008    0.396    0.692    0.033    0.033
   .x1                0.000                                0.000    0.000
   .x2                0.000                                0.000    0.000
   .x3                0.000                                0.000    0.000
```

```
    .x4                0.000                                       0.000    0.000
    .x5                0.000                                       0.000    0.000
     age_M00           0.000                                       0.000    0.000
     edyears           0.000                                       0.000    0.000
     sex               0.000                                       0.000    0.000
     vsclr_rsk_smcr    0.000                                       0.000    0.000
     eTIV              0.000                                       0.000    0.000

Variances:
                    Estimate  Std.Err  z-value  P(>|z|)   Std.lv  Std.all
    .iy                0.808    0.049   16.409    0.000    0.811    0.811
    .sy                0.004    0.001    4.934    0.000    0.987    0.987
    .y1      (tht_y)   0.018    0.002   10.485    0.000    0.018    0.017
    .y2      (tht_y)   0.018    0.002   10.485    0.000    0.018    0.017
    .y3      (tht_y)   0.018    0.002   10.485    0.000    0.018    0.017
    .y4      (tht_y)   0.018    0.002   10.485    0.000    0.018    0.017
    .y5      (tht_y)   0.018    0.002   10.485    0.000    0.018    0.016
    .ix                0.719    0.058   12.480    0.000    0.840    0.840
    .sx                0.009    0.002    3.553    0.000    0.965    0.965
    .x1      (tht_x)   0.121    0.010   12.002    0.000    0.121    0.124
    .x2      (tht_x)   0.121    0.010   12.002    0.000    0.121    0.121
    .x3      (tht_x)   0.121    0.010   12.002    0.000    0.121    0.116
    .x4      (tht_x)   0.121    0.010   12.002    0.000    0.121    0.110
    .x5      (tht_x)   0.121    0.010   12.002    0.000    0.121    0.103
     ag_M00            1.000                                       1.000    1.000
     sex               1.000                                       1.000    1.000
     edyers            1.000                                       1.000    1.000
     vscl__            1.000                                       1.000    1.000
     eTIV              1.000                                       1.000    1.000

R-Square:
                    Estimate
     iy                0.189
     sy                0.013
     y1                0.983
     y2                0.983
     y3                0.983
     y4                0.983
     y5                0.984
     ix                0.160
     sx                0.035
     x1                0.876
     x2                0.879
     x3                0.884
     x4                0.890
```

```
    x5                 0.897
```

Standardised solution

```
Latent Variables:
                      est.std  Std.Err  z-value  P(>|z|) ci.lower ci.upper
  iy =~
    y1                  0.991    0.001 1075.926    0.000    0.990    0.993
    y2                  0.990    0.005  215.663    0.000    0.981    0.999
    y3                  0.985    0.009  115.484    0.000    0.968    1.001
    y4                  0.976    0.012   80.400    0.000    0.952    0.999
    y5                  0.963    0.015   62.529    0.000    0.933    0.994
  sy =~
    y1                  0.000                               0.000    0.000
    y2                  0.063    0.007    9.603    0.000    0.050    0.076
    y3                  0.125    0.013    9.539    0.000    0.099    0.150
    y4                  0.185    0.019    9.537    0.000    0.147    0.224
    y5                  0.244    0.025    9.594    0.000    0.194    0.294
  ix =~
    x1                  0.936    0.007  136.771    0.000    0.923    0.950
    x2                  0.925    0.012   76.024    0.000    0.901    0.949
    x3                  0.907    0.018   49.401    0.000    0.871    0.943
    x4                  0.882    0.024   37.420    0.000    0.836    0.928
    x5                  0.853    0.028   30.710    0.000    0.798    0.907
  sx =~
    x1                  0.000                               0.000    0.000
    x2                  0.094    0.013    7.173    0.000    0.068    0.120
    x3                  0.185    0.026    7.132    0.000    0.134    0.235
    x4                  0.270    0.037    7.191    0.000    0.196    0.343
    x5                  0.348    0.047    7.340    0.000    0.255    0.440

Regressions:
                      est.std  Std.Err  z-value  P(>|z|) ci.lower ci.upper
  iy ~
    age_M00             0.374    0.042    8.936    0.000    0.292    0.456
    sex                 0.189    0.060    3.156    0.002    0.071    0.306
    edyears            -0.027    0.044   -0.622    0.534   -0.114    0.059
    vsclr_rsk_smcr      0.102    0.044    2.307    0.021    0.015    0.188
    eTIV                0.260    0.054    4.779    0.000    0.153    0.366
  sy ~
    age_M00            -0.079    0.069   -1.152    0.249   -0.214    0.056
    sex                 0.010    0.088    0.109    0.913   -0.164    0.183
    edyears            -0.069    0.067   -1.030    0.303   -0.200    0.062
    vsclr_rsk_smcr     -0.021    0.066   -0.317    0.751   -0.151    0.109
```

```
    eTIV              -0.025    0.075   -0.331    0.741   -0.172    0.122
  ix ~
    age_M00           -0.352    0.045   -7.759    0.000   -0.441   -0.263
    sex               -0.037    0.065   -0.564    0.573   -0.165    0.091
    edyears           -0.076    0.051   -1.505    0.132   -0.176    0.023
    vsclr_rsk_smcr    -0.085    0.051   -1.683    0.092   -0.184    0.014
    eTIV              -0.143    0.065   -2.195    0.028   -0.271   -0.015
  sx ~
    age_M00           -0.143    0.088   -1.631    0.103   -0.316    0.029
    sex                0.093    0.110    0.842    0.400   -0.123    0.308
    edyears            0.080    0.082    0.976    0.329   -0.081    0.242
    vsclr_rsk_smcr     0.064    0.091    0.707    0.480   -0.114    0.242
    eTIV               0.051    0.109    0.470    0.638   -0.163    0.266

Covariances:
                          est.std  Std.Err  z-value  P(>|z|) ci.lower ci.upper
 .iy ~~
   .sy                      0.027    0.072    0.380    0.704   -0.114    0.168
 .ix ~~
   .sx                      0.043    0.123    0.350    0.726   -0.198    0.284
 .y1 ~~
   .x1      (tht_)         -0.040    0.034   -1.192    0.233   -0.107    0.026
 .y2 ~~
   .x2      (tht_)         -0.040    0.034   -1.192    0.233   -0.107    0.026
 .y3 ~~
   .x3      (tht_)         -0.040    0.034   -1.192    0.233   -0.107    0.026
 .y4 ~~
   .x4      (tht_)         -0.040    0.034   -1.192    0.233   -0.107    0.026
 .y5 ~~
   .x5      (tht_)         -0.040    0.034   -1.192    0.233   -0.107    0.026
 .iy ~~
   .ix                     -0.126    0.050   -2.505    0.012   -0.225   -0.027
   .sx                     -0.124    0.087   -1.430    0.153   -0.295    0.046
 .sy ~~
   .ix                     -0.088    0.064   -1.383    0.167   -0.212    0.037
   .sx                     -0.261    0.132   -1.982    0.047   -0.519   -0.003
  age_M00 ~~
    sex                    -0.165    0.046   -3.607    0.000   -0.255   -0.075
    edyears                -0.119    0.048   -2.478    0.013   -0.214   -0.025
    vsclr__                 0.157    0.047    3.335    0.001    0.065    0.249
    eTIV                    0.057    0.046    1.234    0.217   -0.033    0.146
  sex ~~
    edyears                -0.238    0.043   -5.569    0.000   -0.321   -0.154
    vsclr__                -0.184    0.045   -4.128    0.000   -0.272   -0.097
    eTIV                   -0.668    0.021  -31.365    0.000   -0.710   -0.626
```

```
  edyears ~~
    vsclr__                -0.156    0.043   -3.625    0.000   -0.241   -0.072
    eTIV                    0.247    0.042    5.956    0.000    0.166    0.329
  vascular_risk_sumcorr ~~
    eTIV                    0.113    0.048    2.362    0.018    0.019    0.207

Intercepts:
                   est.std  Std.Err  z-value  P(>|z|) ci.lower ci.upper
   .iy              -0.131    0.043   -3.046    0.002   -0.215   -0.047
   .sy               1.112    0.109   10.228    0.000    0.899    1.325
   .y1               0.000                               0.000    0.000
   .y2               0.000                               0.000    0.000
   .y3               0.000                               0.000    0.000
   .y4               0.000                               0.000    0.000
   .y5               0.000                               0.000    0.000
   .ix              -0.007    0.047   -0.147    0.883   -0.100    0.086
   .sx               0.033    0.084    0.397    0.691   -0.132    0.198
   .x1               0.000                               0.000    0.000
   .x2               0.000                               0.000    0.000
   .x3               0.000                               0.000    0.000
   .x4               0.000                               0.000    0.000
   .x5               0.000                               0.000    0.000
    age_M00          0.000                               0.000    0.000
    edyears          0.000                               0.000    0.000
    sex              0.000                               0.000    0.000
    vsclr_rsk_smcr   0.000                               0.000    0.000
    eTIV             0.000                               0.000    0.000

Variances:
                   est.std  Std.Err  z-value  P(>|z|) ci.lower ci.upper
   .iy               0.811    0.032   25.311    0.000    0.748    0.873
   .sy               0.987    0.015   66.643    0.000    0.958    1.016
   .y1     (tht_y)   0.017    0.002    9.464    0.000    0.014    0.021
   .y2     (tht_y)   0.017    0.002    9.597    0.000    0.014    0.021
   .y3     (tht_y)   0.017    0.002    9.672    0.000    0.014    0.021
   .y4     (tht_y)   0.017    0.002    9.692    0.000    0.013    0.020
   .y5     (tht_y)   0.016    0.002    9.662    0.000    0.013    0.020
   .ix               0.840    0.035   24.131    0.000    0.772    0.908
   .sx               0.965    0.031   31.543    0.000    0.905    1.025
   .x1     (tht_x)   0.124    0.013    9.653    0.000    0.099    0.149
   .x2     (tht_x)   0.121    0.012   10.010    0.000    0.097    0.144
   .x3     (tht_x)   0.116    0.011   10.112    0.000    0.094    0.139
   .x4     (tht_x)   0.110    0.011   10.009    0.000    0.088    0.131
   .x5     (tht_x)   0.103    0.011    9.755    0.000    0.082    0.123
    ag_M00           1.000                               1.000    1.000
```

sex	1.000	1.000	1.000
edyers	1.000	1.000	1.000
vscl__	1.000	1.000	1.000
eTIV	1.000	1.000	1.000

BLGCM - Total WMH volumes vs parietal cortical thickness

Unstandardised solution

```
lavaan 0.6.17 ended normally after 124 iterations

  Estimator                                         ML
  Optimization method                           NLMINB
  Number of model parameters                        59
  Number of equality constraints                    12

  Number of observations                           451
  Number of missing patterns                        74

Model Test User Model:
                                              Standard      Scaled
  Test Statistic                                99.225      81.042
  Degrees of freedom                                88          88
  P-value (Chi-square)                           0.194       0.687
  Scaling correction factor                                  1.224
    Yuan-Bentler correction (Mplus variant)

Model Test Baseline Model:

  Test statistic                              6982.351    5228.556
  Degrees of freedom                               105         105
  P-value                                        0.000       0.000
  Scaling correction factor                                  1.335

User Model versus Baseline Model:

  Comparative Fit Index (CFI)                    0.998       1.000
  Tucker-Lewis Index (TLI)                       0.998       1.002

  Robust Comparative Fit Index (CFI)                         1.000
  Robust Tucker-Lewis Index (TLI)                            1.002

Loglikelihood and Information Criteria:

  Loglikelihood user model (H0)              -4721.946   -4721.946
  Scaling correction factor                                  0.999
      for the MLR correction
  Loglikelihood unrestricted model (H1)      -4672.333   -4672.333
  Scaling correction factor                                  1.235
```

```
    for the MLR correction

  Akaike (AIC)                                9537.891    9537.891
  Bayesian (BIC)                              9731.130    9731.130
  Sample-size adjusted Bayesian (SABIC)       9581.970    9581.970

Root Mean Square Error of Approximation:

  RMSEA                                          0.017       0.000
  90 Percent confidence interval - lower         0.000       0.000
  90 Percent confidence interval - upper         0.032       0.019
  P-value H_0: RMSEA <= 0.050                    1.000       1.000
  P-value H_0: RMSEA >= 0.080                    0.000       0.000

  Robust RMSEA                                               0.000
  90 Percent confidence interval - lower                     0.000
  90 Percent confidence interval - upper                     0.029
  P-value H_0: Robust RMSEA <= 0.050                         1.000
  P-value H_0: Robust RMSEA >= 0.080                         0.000

Standardized Root Mean Square Residual:

  SRMR                                           0.013       0.013

Parameter Estimates:

  Standard errors                             Sandwich
  Information bread                           Observed
  Observed information based on                Hessian

Latent Variables:
                   Estimate  Std.Err  z-value  P(>|z|)   Std.lv  Std.all
  iy =~
    y1                1.000                               0.998    0.991
    y2                1.000                               0.998    0.990
    y3                1.000                               0.998    0.984
    y4                1.000                               0.998    0.975
    y5                1.000                               0.998    0.963
  sy =~
    y1                0.000                               0.000    0.000
    y2                1.000                               0.063    0.063
    y3                2.000                               0.126    0.125
    y4                3.000                               0.190    0.185
    y5                4.000                               0.253    0.244
  ix =~
```

```
    x1                1.000                               0.918    0.930
    x2                1.000                               0.918    0.923
    x3                1.000                               0.918    0.910
    x4                1.000                               0.918    0.892
    x5                1.000                               0.918    0.871
  sx =~
    x1                0.000                               0.000    0.000
    x2                1.000                               0.079    0.080
    x3                2.000                               0.159    0.158
    x4                3.000                               0.238    0.232
    x5                4.000                               0.318    0.301

Regressions:
                   Estimate  Std.Err  z-value  P(>|z|)   Std.lv  Std.all
  iy ~
    age_M00           0.373    0.044    8.437    0.000    0.374    0.374
    sex               0.188    0.060    3.128    0.002    0.189    0.189
    edyears          -0.027    0.044   -0.621    0.535   -0.027   -0.027
    vsclr_rsk_smcr    0.102    0.045    2.273    0.023    0.102    0.102
    eTIV              0.259    0.056    4.637    0.000    0.260    0.260
  sy ~
    age_M00          -0.005    0.004   -1.094    0.274   -0.076   -0.076
    sex               0.000    0.006    0.083    0.934    0.007    0.007
    edyears          -0.004    0.004   -1.052    0.293   -0.070   -0.070
    vsclr_rsk_smcr   -0.001    0.004   -0.315    0.753   -0.021   -0.021
    eTIV             -0.002    0.005   -0.351    0.726   -0.027   -0.027
  ix ~
    age_M00          -0.334    0.043   -7.680    0.000   -0.364   -0.364
    sex               0.031    0.060    0.514    0.607    0.034    0.034
    edyears          -0.059    0.047   -1.252    0.211   -0.064   -0.064
    vsclr_rsk_smcr   -0.034    0.048   -0.715    0.475   -0.037   -0.037
    eTIV             -0.091    0.061   -1.501    0.133   -0.099   -0.099
  sx ~
    age_M00          -0.012    0.008   -1.430    0.153   -0.147   -0.147
    sex               0.005    0.010    0.532    0.595    0.066    0.066
    edyears           0.010    0.008    1.278    0.201    0.121    0.121
    vsclr_rsk_smcr   -0.004    0.009   -0.400    0.689   -0.045   -0.045
    eTIV              0.006    0.010    0.585    0.558    0.074    0.074

Covariances:
                           Estimate  Std.Err  z-value  P(>|z|)   Std.lv  Std.all
 .iy ~~
   .sy                        0.002    0.004    0.393    0.694    0.028    0.028
 .ix ~~
   .sx                        0.001    0.009    0.076    0.939    0.010    0.010
```

```
  .y1 ~~
    .x1      (tht_)            0.001    0.002    0.818    0.413    0.001    0.026
  .y2 ~~
    .x2      (tht_)            0.001    0.002    0.818    0.413    0.001    0.026
  .y3 ~~
    .x3      (tht_)            0.001    0.002    0.818    0.413    0.001    0.026
  .y4 ~~
    .x4      (tht_)            0.001    0.002    0.818    0.413    0.001    0.026
  .y5 ~~
    .x5      (tht_)            0.001    0.002    0.818    0.413    0.001    0.026
  .iy ~~
    .ix                       -0.059    0.037   -1.594    0.111   -0.079   -0.079
    .sx                       -0.012    0.007   -1.658    0.097   -0.170   -0.170
  .sy ~~
    .ix                       -0.003    0.003   -0.758    0.449   -0.048   -0.048
    .sx                       -0.001    0.001   -1.145    0.252   -0.179   -0.179
   age_M00 ~~
     sex                      -0.165    0.046   -3.607    0.000   -0.165   -0.165
     edyears                  -0.119    0.048   -2.478    0.013   -0.119   -0.119
     vsclr__                   0.157    0.047    3.335    0.001    0.157    0.157
     eTIV                      0.057    0.046    1.234    0.217    0.057    0.057
   sex ~~
     edyears                  -0.238    0.043   -5.569    0.000   -0.238   -0.238
     vsclr__                  -0.184    0.045   -4.128    0.000   -0.184   -0.184
     eTIV                     -0.668    0.021  -31.365    0.000   -0.668   -0.668
   edyears ~~
     vsclr__                  -0.156    0.043   -3.625    0.000   -0.156   -0.156
     eTIV                      0.247    0.042    5.956    0.000    0.247    0.247
   vascular_risk_sumcorr ~~
     eTIV                      0.113    0.048    2.362    0.018    0.113    0.113

Intercepts:
                   Estimate  Std.Err  z-value  P(>|z|)   Std.lv  Std.all
    .iy              -0.131    0.043   -3.065    0.002   -0.131   -0.131
    .sy               0.070    0.004   17.682    0.000    1.114    1.114
    .y1               0.000                               0.000    0.000
    .y2               0.000                               0.000    0.000
    .y3               0.000                               0.000    0.000
    .y4               0.000                               0.000    0.000
    .y5               0.000                               0.000    0.000
    .ix               0.047    0.043    1.074    0.283    0.051    0.051
    .sx              -0.022    0.008   -2.786    0.005   -0.274   -0.274
    .x1               0.000                               0.000    0.000
    .x2               0.000                               0.000    0.000
    .x3               0.000                               0.000    0.000
```

```
   .x4                0.000                                      0.000    0.000
   .x5                0.000                                      0.000    0.000
    age_M00           0.000                                      0.000    0.000
    edyears           0.000                                      0.000    0.000
    sex               0.000                                      0.000    0.000
    vsclr_rsk_smcr    0.000                                      0.000    0.000
    eTIV              0.000                                      0.000    0.000

Variances:
                   Estimate  Std.Err  z-value  P(>|z|)   Std.lv  Std.all
   .iy                0.808    0.049   16.417    0.000    0.811    0.811
   .sy                0.004    0.001    4.911    0.000    0.987    0.987
   .y1      (tht_y)   0.018    0.002   10.479    0.000    0.018    0.017
   .y2      (tht_y)   0.018    0.002   10.479    0.000    0.018    0.017
   .y3      (tht_y)   0.018    0.002   10.479    0.000    0.018    0.017
   .y4      (tht_y)   0.018    0.002   10.479    0.000    0.018    0.017
   .y5      (tht_y)   0.018    0.002   10.479    0.000    0.018    0.016
   .ix                0.704    0.060   11.735    0.000    0.835    0.835
   .sx                0.006    0.002    2.453    0.014    0.947    0.947
   .x1      (tht_x)   0.131    0.015    8.960    0.000    0.131    0.135
   .x2      (tht_x)   0.131    0.015    8.960    0.000    0.131    0.132
   .x3      (tht_x)   0.131    0.015    8.960    0.000    0.131    0.129
   .x4      (tht_x)   0.131    0.015    8.960    0.000    0.131    0.124
   .x5      (tht_x)   0.131    0.015    8.960    0.000    0.131    0.118
    ag_M00            1.000                                      1.000    1.000
    sex               1.000                                      1.000    1.000
    edyers            1.000                                      1.000    1.000
    vscl__            1.000                                      1.000    1.000
    eTIV              1.000                                      1.000    1.000

R-Square:
                   Estimate
    iy                0.189
    sy                0.013
    y1                0.983
    y2                0.983
    y3                0.983
    y4                0.983
    y5                0.984
    ix                0.165
    sx                0.053
    x1                0.865
    x2                0.868
    x3                0.871
    x4                0.876
```

```
    x5                  0.882
```

Standardised solution

```
Latent Variables:
                     est.std  Std.Err  z-value  P(>|z|) ci.lower ci.upper
  iy =~
    y1                 0.991    0.001 1074.186    0.000    0.989    0.993
    y2                 0.990    0.005  215.152    0.000    0.981    0.999
    y3                 0.984    0.009  115.272    0.000    0.968    1.001
    y4                 0.975    0.012   80.294    0.000    0.952    0.999
    y5                 0.963    0.015   62.478    0.000    0.933    0.993
  sy =~
    y1                 0.000                               0.000    0.000
    y2                 0.063    0.007    9.557    0.000    0.050    0.075
    y3                 0.125    0.013    9.493    0.000    0.099    0.150
    y4                 0.185    0.020    9.490    0.000    0.147    0.223
    y5                 0.244    0.026    9.546    0.000    0.194    0.294
  ix =~
    x1                 0.930    0.009  102.377    0.000    0.912    0.948
    x2                 0.923    0.013   68.787    0.000    0.897    0.949
    x3                 0.910    0.019   48.779    0.000    0.874    0.947
    x4                 0.892    0.023   38.031    0.000    0.846    0.938
    x5                 0.871    0.028   31.310    0.000    0.816    0.925
  sx =~
    x1                 0.000                               0.000    0.000
    x2                 0.080    0.016    4.975    0.000    0.048    0.111
    x3                 0.158    0.032    4.964    0.000    0.095    0.220
    x4                 0.232    0.046    5.006    0.000    0.141    0.322
    x5                 0.301    0.059    5.098    0.000    0.186    0.417

Regressions:
                     est.std  Std.Err  z-value  P(>|z|) ci.lower ci.upper
  iy ~
    age_M00            0.374    0.042    8.929    0.000    0.292    0.456
    sex                0.189    0.060    3.160    0.002    0.072    0.306
    edyears           -0.027    0.044   -0.622    0.534   -0.114    0.059
    vsclr_rsk_smcr     0.102    0.044    2.305    0.021    0.015    0.188
    eTIV               0.260    0.054    4.784    0.000    0.153    0.366
  sy ~
    age_M00           -0.076    0.069   -1.104    0.269   -0.211    0.059
    sex                0.007    0.089    0.083    0.934   -0.166    0.181
    edyears           -0.070    0.067   -1.042    0.297   -0.201    0.061
    vsclr_rsk_smcr    -0.021    0.066   -0.316    0.752   -0.151    0.109
```

```
    eTIV              -0.027    0.075   -0.354    0.723   -0.174    0.121
  ix ~
    age_M00           -0.364    0.046   -7.949    0.000   -0.453   -0.274
    sex                0.034    0.066    0.513    0.608   -0.095    0.162
    edyears           -0.064    0.051   -1.251    0.211   -0.164    0.036
    vsclr_rsk_smcr    -0.037    0.052   -0.717    0.473   -0.140    0.065
    eTIV              -0.099    0.065   -1.513    0.130   -0.227    0.029
  sx ~
    age_M00           -0.147    0.102   -1.444    0.149   -0.347    0.053
    sex                0.066    0.124    0.534    0.593   -0.177    0.309
    edyears            0.121    0.092    1.316    0.188   -0.059    0.302
    vsclr_rsk_smcr    -0.045    0.112   -0.402    0.688   -0.264    0.174
    eTIV               0.074    0.129    0.578    0.563   -0.178    0.327

Covariances:
                           est.std  Std.Err  z-value  P(>|z|) ci.lower ci.upper
 .iy ~~
   .sy                       0.028    0.072    0.387    0.699   -0.114    0.170
 .ix ~~
   .sx                       0.010    0.135    0.075    0.940   -0.255    0.275
 .y1 ~~
   .x1      (tht_)           0.026    0.032    0.827    0.408   -0.036    0.089
 .y2 ~~
   .x2      (tht_)           0.026    0.032    0.827    0.408   -0.036    0.089
 .y3 ~~
   .x3      (tht_)           0.026    0.032    0.827    0.408   -0.036    0.089
 .y4 ~~
   .x4      (tht_)           0.026    0.032    0.827    0.408   -0.036    0.089
 .y5 ~~
   .x5      (tht_)           0.026    0.032    0.827    0.408   -0.036    0.089
 .iy ~~
   .ix                      -0.079    0.049   -1.600    0.110   -0.175    0.018
   .sx                      -0.170    0.101   -1.690    0.091   -0.368    0.027
 .sy ~~
   .ix                      -0.048    0.062   -0.776    0.438   -0.169    0.073
   .sx                      -0.179    0.161   -1.111    0.267   -0.495    0.137
  age_M00 ~~
    sex                     -0.165    0.046   -3.607    0.000   -0.255   -0.075
    edyears                 -0.119    0.048   -2.478    0.013   -0.214   -0.025
    vsclr__                  0.157    0.047    3.335    0.001    0.065    0.249
    eTIV                     0.057    0.046    1.234    0.217   -0.033    0.146
  sex ~~
    edyears                 -0.238    0.043   -5.569    0.000   -0.321   -0.154
    vsclr__                 -0.184    0.045   -4.128    0.000   -0.272   -0.097
    eTIV                    -0.668    0.021  -31.365    0.000   -0.710   -0.626
```

```
  edyears ~~
    vsclr__                 -0.156    0.043   -3.625    0.000   -0.241   -0.072
    eTIV                     0.247    0.042    5.956    0.000    0.166    0.329
  vascular_risk_sumcorr ~~
    eTIV                     0.113    0.048    2.362    0.018    0.019    0.207

Intercepts:
                    est.std  Std.Err  z-value  P(>|z|) ci.lower ci.upper
   .iy               -0.131    0.043   -3.044    0.002   -0.215   -0.047
   .sy                1.114    0.109   10.187    0.000    0.900    1.328
   .y1                0.000                                0.000    0.000
   .y2                0.000                                0.000    0.000
   .y3                0.000                                0.000    0.000
   .y4                0.000                                0.000    0.000
   .y5                0.000                                0.000    0.000
   .ix                0.051    0.047    1.075    0.282   -0.042    0.144
   .sx               -0.274    0.113   -2.426    0.015   -0.496   -0.053
   .x1                0.000                                0.000    0.000
   .x2                0.000                                0.000    0.000
   .x3                0.000                                0.000    0.000
   .x4                0.000                                0.000    0.000
   .x5                0.000                                0.000    0.000
    age_M00           0.000                                0.000    0.000
    edyears           0.000                                0.000    0.000
    sex               0.000                                0.000    0.000
    vsclr_rsk_smcr    0.000                                0.000    0.000
    eTIV              0.000                                0.000    0.000

Variances:
                    est.std  Std.Err  z-value  P(>|z|) ci.lower ci.upper
   .iy                0.811    0.032   25.314    0.000    0.748    0.874
   .sy                0.987    0.015   67.762    0.000    0.959    1.016
   .y1     (tht_y)    0.017    0.002    9.460    0.000    0.014    0.021
   .y2     (tht_y)    0.017    0.002    9.594    0.000    0.014    0.021
   .y3     (tht_y)    0.017    0.002    9.669    0.000    0.014    0.021
   .y4     (tht_y)    0.017    0.002    9.688    0.000    0.013    0.020
   .y5     (tht_y)    0.016    0.002    9.657    0.000    0.013    0.020
   .ix                0.835    0.035   23.604    0.000    0.766    0.905
   .sx                0.947    0.042   22.435    0.000    0.864    1.030
   .x1     (tht_x)    0.135    0.017    7.959    0.000    0.101    0.168
   .x2     (tht_x)    0.132    0.016    8.206    0.000    0.101    0.164
   .x3     (tht_x)    0.129    0.016    8.278    0.000    0.098    0.159
   .x4     (tht_x)    0.124    0.015    8.191    0.000    0.094    0.153
   .x5     (tht_x)    0.118    0.015    7.965    0.000    0.089    0.147
    ag_M00            1.000                                1.000    1.000
```

sex	1.000	1.000	1.000
edyers	1.000	1.000	1.000
vscl__	1.000	1.000	1.000
eTIV	1.000	1.000	1.000

BLGCM - Total WMH volumes vs occipital cortical thickness

Unstandardised solution

```
lavaan 0.6.17 ended normally after 121 iterations

  Estimator                                         ML
  Optimization method                           NLMINB
  Number of model parameters                        59
  Number of equality constraints                    12

  Number of observations                           451
  Number of missing patterns                        76

Model Test User Model:
                                              Standard      Scaled
  Test Statistic                               106.896      88.886
  Degrees of freedom                                88          88
  P-value (Chi-square)                           0.083       0.454
  Scaling correction factor                                  1.203
    Yuan-Bentler correction (Mplus variant)

Model Test Baseline Model:

  Test statistic                              6920.259    5301.418
  Degrees of freedom                               105         105
  P-value                                        0.000       0.000
  Scaling correction factor                                  1.305

User Model versus Baseline Model:

  Comparative Fit Index (CFI)                    0.997       1.000
  Tucker-Lewis Index (TLI)                       0.997       1.000

  Robust Comparative Fit Index (CFI)                         1.000
  Robust Tucker-Lewis Index (TLI)                            1.000

Loglikelihood and Information Criteria:

  Loglikelihood user model (H0)              -4773.692   -4773.692
  Scaling correction factor                                  0.978
      for the MLR correction
  Loglikelihood unrestricted model (H1)      -4720.244   -4720.244
  Scaling correction factor                                  1.211
```

```
      for the MLR correction

  Akaike (AIC)                                9641.384    9641.384
  Bayesian (BIC)                              9834.623    9834.623
  Sample-size adjusted Bayesian (SABIC)       9685.462    9685.462

Root Mean Square Error of Approximation:

  RMSEA                                          0.022       0.005
  90 Percent confidence interval - lower         0.000       0.000
  90 Percent confidence interval - upper         0.035       0.025
  P-value H_0: RMSEA <= 0.050                    1.000       1.000
  P-value H_0: RMSEA >= 0.080                    0.000       0.000

  Robust RMSEA                                               0.000
  90 Percent confidence interval - lower                     0.000
  90 Percent confidence interval - upper                     0.036
  P-value H_0: Robust RMSEA <= 0.050                         0.998
  P-value H_0: Robust RMSEA >= 0.080                         0.000

Standardized Root Mean Square Residual:

  SRMR                                           0.013       0.013

Parameter Estimates:

  Standard errors                             Sandwich
  Information bread                           Observed
  Observed information based on                Hessian

Latent Variables:
                   Estimate  Std.Err  z-value  P(>|z|)   Std.lv  Std.all
  iy =~
    y1                1.000                               0.998    0.991
    y2                1.000                               0.998    0.990
    y3                1.000                               0.998    0.984
    y4                1.000                               0.998    0.975
    y5                1.000                               0.998    0.963
  sy =~
    y1                0.000                               0.000    0.000
    y2                1.000                               0.063    0.063
    y3                2.000                               0.126    0.125
    y4                3.000                               0.190    0.185
    y5                4.000                               0.253    0.244
  ix =~
```

```
    x1                1.000                               0.920    0.928
    x2                1.000                               0.920    0.921
    x3                1.000                               0.920    0.909
    x4                1.000                               0.920    0.891
    x5                1.000                               0.920    0.869
  sx =~
    x1                0.000                               0.000    0.000
    x2                1.000                               0.082    0.082
    x3                2.000                               0.163    0.161
    x4                3.000                               0.245    0.237
    x5                4.000                               0.326    0.308

Regressions:
                   Estimate  Std.Err  z-value  P(>|z|)   Std.lv  Std.all
  iy ~
    age_M00           0.373    0.044    8.438    0.000    0.374    0.374
    sex               0.188    0.060    3.125    0.002    0.189    0.189
    edyears          -0.027    0.044   -0.620    0.535   -0.027   -0.027
    vsclr_rsk_smcr    0.102    0.045    2.274    0.023    0.102    0.102
    eTIV              0.259    0.056    4.635    0.000    0.260    0.260
  sy ~
    age_M00          -0.005    0.004   -1.086    0.277   -0.075   -0.075
    sex               0.001    0.006    0.107    0.915    0.009    0.009
    edyears          -0.004    0.004   -1.072    0.284   -0.071   -0.071
    vsclr_rsk_smcr   -0.001    0.004   -0.350    0.727   -0.023   -0.023
    eTIV             -0.002    0.005   -0.316    0.752   -0.024   -0.024
  ix ~
    age_M00          -0.233    0.048   -4.899    0.000   -0.253   -0.253
    sex               0.082    0.065    1.268    0.205    0.089    0.089
    edyears           0.021    0.048    0.429    0.668    0.022    0.022
    vsclr_rsk_smcr   -0.023    0.045   -0.522    0.602   -0.025   -0.025
    eTIV             -0.000    0.061   -0.002    0.999   -0.000   -0.000
  sx ~
    age_M00          -0.019    0.009   -2.091    0.037   -0.227   -0.227
    sex              -0.004    0.011   -0.374    0.708   -0.049   -0.049
    edyears           0.010    0.008    1.270    0.204    0.122    0.122
    vsclr_rsk_smcr    0.004    0.008    0.516    0.606    0.053    0.053
    eTIV              0.000    0.012    0.026    0.979    0.004    0.004

Covariances:
                           Estimate  Std.Err  z-value  P(>|z|)   Std.lv  Std.all
 .iy ~~
   .sy                        0.002    0.004    0.380    0.704    0.027    0.027
 .ix ~~
   .sx                       -0.000    0.008   -0.041    0.968   -0.004   -0.004
```

```
 .y1 ~~
   .x1      (tht_)            0.000    0.002    0.208    0.835    0.000    0.007
 .y2 ~~
   .x2      (tht_)            0.000    0.002    0.208    0.835    0.000    0.007
 .y3 ~~
   .x3      (tht_)            0.000    0.002    0.208    0.835    0.000    0.007
 .y4 ~~
   .x4      (tht_)            0.000    0.002    0.208    0.835    0.000    0.007
 .y5 ~~
   .x5      (tht_)            0.000    0.002    0.208    0.835    0.000    0.007
 .iy ~~
   .ix                       -0.097    0.040   -2.436    0.015   -0.123   -0.123
   .sx                       -0.007    0.007   -1.052    0.293   -0.102   -0.102
 .sy ~~
   .ix                       -0.004    0.004   -1.062    0.288   -0.071   -0.071
   .sx                       -0.002    0.001   -2.144    0.032   -0.315   -0.315
  age_M00 ~~
    sex                      -0.165    0.046   -3.607    0.000   -0.165   -0.165
    edyears                  -0.119    0.048   -2.478    0.013   -0.119   -0.119
    vsclr__                   0.157    0.047    3.335    0.001    0.157    0.157
    eTIV                      0.057    0.046    1.234    0.217    0.057    0.057
  sex ~~
    edyears                  -0.238    0.043   -5.569    0.000   -0.238   -0.238
    vsclr__                  -0.184    0.045   -4.128    0.000   -0.184   -0.184
    eTIV                     -0.668    0.021  -31.365    0.000   -0.668   -0.668
  edyears ~~
    vsclr__                  -0.156    0.043   -3.625    0.000   -0.156   -0.156
    eTIV                      0.247    0.042    5.956    0.000    0.247    0.247
  vascular_risk_sumcorr ~~
    eTIV                      0.113    0.048    2.362    0.018    0.113    0.113

Intercepts:
                   Estimate  Std.Err  z-value  P(>|z|)   Std.lv  Std.all
   .iy               -0.130    0.043   -3.064    0.002   -0.131   -0.131
   .sy                0.070    0.004   17.740    0.000    1.113    1.113
   .y1                0.000                               0.000    0.000
   .y2                0.000                               0.000    0.000
   .y3                0.000                               0.000    0.000
   .y4                0.000                               0.000    0.000
   .y5                0.000                               0.000    0.000
   .ix               -0.019    0.045   -0.411    0.681   -0.020   -0.020
   .sx                0.010    0.008    1.284    0.199    0.126    0.126
   .x1                0.000                               0.000    0.000
   .x2                0.000                               0.000    0.000
   .x3                0.000                               0.000    0.000
```

```
   .x4                0.000                               0.000    0.000
   .x5                0.000                               0.000    0.000
    age_M00           0.000                               0.000    0.000
    edyears           0.000                               0.000    0.000
    sex               0.000                               0.000    0.000
    vsclr_rsk_smcr    0.000                               0.000    0.000
    eTIV              0.000                               0.000    0.000

Variances:
                   Estimate  Std.Err  z-value  P(>|z|)   Std.lv  Std.all
   .iy                0.808    0.049   16.423    0.000    0.811    0.811
   .sy                0.004    0.001    4.917    0.000    0.987    0.987
   .y1      (tht_y)   0.018    0.002   10.502    0.000    0.018    0.017
   .y2      (tht_y)   0.018    0.002   10.502    0.000    0.018    0.017
   .y3      (tht_y)   0.018    0.002   10.502    0.000    0.018    0.017
   .y4      (tht_y)   0.018    0.002   10.502    0.000    0.018    0.017
   .y5      (tht_y)   0.018    0.002   10.502    0.000    0.018    0.016
   .ix                0.775    0.057   13.579    0.000    0.916    0.916
   .sx                0.006    0.002    2.822    0.005    0.927    0.927
   .x1      (tht_x)   0.137    0.015    9.418    0.000    0.137    0.139
   .x2      (tht_x)   0.137    0.015    9.418    0.000    0.137    0.137
   .x3      (tht_x)   0.137    0.015    9.418    0.000    0.137    0.133
   .x4      (tht_x)   0.137    0.015    9.418    0.000    0.137    0.128
   .x5      (tht_x)   0.137    0.015    9.418    0.000    0.137    0.122
    ag_M00            1.000                               1.000    1.000
    sex               1.000                               1.000    1.000
    edyers            1.000                               1.000    1.000
    vscl__            1.000                               1.000    1.000
    eTIV              1.000                               1.000    1.000

R-Square:
                   Estimate
    iy                0.189
    sy                0.013
    y1                0.983
    y2                0.983
    y3                0.983
    y4                0.983
    y5                0.984
    ix                0.084
    sx                0.073
    x1                0.861
    x2                0.863
    x3                0.867
    x4                0.872
```

```
    x5                0.878
```

Standardised solution

```
Latent Variables:
                   est.std  Std.Err  z-value  P(>|z|) ci.lower ci.upper
  iy =~
    y1               0.991    0.001 1077.087    0.000    0.990    0.993
    y2               0.990    0.005  216.090    0.000    0.981    0.999
    y3               0.984    0.009  115.664    0.000    0.968    1.001
    y4               0.975    0.012   80.504    0.000    0.952    0.999
    y5               0.963    0.015   62.593    0.000    0.933    0.993
  sy =~
    y1               0.000                               0.000    0.000
    y2               0.063    0.007    9.570    0.000    0.050    0.076
    y3               0.125    0.013    9.508    0.000    0.099    0.150
    y4               0.185    0.020    9.508    0.000    0.147    0.224
    y5               0.244    0.026    9.566    0.000    0.194    0.294
  ix =~
    x1               0.928    0.009  106.162    0.000    0.911    0.945
    x2               0.921    0.012   78.556    0.000    0.898    0.944
    x3               0.909    0.016   55.614    0.000    0.877    0.941
    x4               0.891    0.021   42.407    0.000    0.850    0.932
    x5               0.869    0.025   34.116    0.000    0.819    0.919
  sx =~
    x1               0.000                               0.000    0.000
    x2               0.082    0.014    5.922    0.000    0.055    0.109
    x3               0.161    0.027    5.933    0.000    0.108    0.214
    x4               0.237    0.039    6.009    0.000    0.160    0.314
    x5               0.308    0.050    6.146    0.000    0.210    0.406

Regressions:
                   est.std  Std.Err  z-value  P(>|z|) ci.lower ci.upper
  iy ~
    age_M00          0.374    0.042    8.929    0.000    0.292    0.456
    sex              0.189    0.060    3.157    0.002    0.071    0.306
    edyears         -0.027    0.044   -0.621    0.535   -0.114    0.059
    vsclr_rsk_smcr   0.102    0.044    2.305    0.021    0.015    0.188
    eTIV             0.260    0.054    4.781    0.000    0.153    0.366
  sy ~
    age_M00         -0.075    0.069   -1.095    0.274   -0.210    0.060
    sex              0.009    0.088    0.107    0.915   -0.164    0.182
    edyears         -0.071    0.067   -1.062    0.288   -0.202    0.060
    vsclr_rsk_smcr  -0.023    0.067   -0.351    0.726   -0.154    0.107
```

```
    eTIV             -0.024    0.075   -0.318    0.750   -0.171    0.123
  ix ~
    age_M00          -0.253    0.051   -5.005    0.000   -0.353   -0.154
    sex               0.089    0.070    1.272    0.203   -0.048    0.226
    edyears           0.022    0.052    0.429    0.668   -0.080    0.125
    vsclr_rsk_smcr   -0.025    0.048   -0.522    0.602   -0.120    0.070
    eTIV             -0.000    0.066   -0.002    0.999   -0.129    0.129
  sx ~
    age_M00          -0.227    0.110   -2.059    0.039   -0.443   -0.011
    sex              -0.049    0.131   -0.376    0.707   -0.306    0.207
    edyears           0.122    0.096    1.275    0.202   -0.066    0.310
    vsclr_rsk_smcr    0.053    0.103    0.514    0.607   -0.149    0.255
    eTIV              0.004    0.150    0.026    0.979   -0.290    0.298

Covariances:
                           est.std  Std.Err  z-value  P(>|z|) ci.lower ci.upper
 .iy ~~
   .sy                       0.027    0.072    0.374    0.708   -0.114    0.168
 .ix ~~
   .sx                      -0.004    0.110   -0.041    0.967   -0.220    0.211
 .y1 ~~
   .x1      (tht_)           0.007    0.032    0.209    0.835   -0.056    0.070
 .y2 ~~
   .x2      (tht_)           0.007    0.032    0.209    0.835   -0.056    0.070
 .y3 ~~
   .x3      (tht_)           0.007    0.032    0.209    0.835   -0.056    0.070
 .y4 ~~
   .x4      (tht_)           0.007    0.032    0.209    0.835   -0.056    0.070
 .y5 ~~
   .x5      (tht_)           0.007    0.032    0.209    0.835   -0.056    0.070
 .iy ~~
   .ix                      -0.123    0.049   -2.480    0.013   -0.220   -0.026
   .sx                      -0.102    0.096   -1.062    0.288   -0.291    0.086
 .sy ~~
   .ix                      -0.071    0.065   -1.093    0.274   -0.199    0.057
   .sx                      -0.315    0.140   -2.255    0.024   -0.589   -0.041
  age_M00 ~~
    sex                     -0.165    0.046   -3.607    0.000   -0.255   -0.075
    edyears                 -0.119    0.048   -2.478    0.013   -0.214   -0.025
    vsclr__                  0.157    0.047    3.335    0.001    0.065    0.249
    eTIV                     0.057    0.046    1.234    0.217   -0.033    0.146
  sex ~~
    edyears                 -0.238    0.043   -5.569    0.000   -0.321   -0.154
    vsclr__                 -0.184    0.045   -4.128    0.000   -0.272   -0.097
    eTIV                    -0.668    0.021  -31.365    0.000   -0.710   -0.626
```

```
  edyears ~~
    vsclr__                -0.156    0.043   -3.625    0.000   -0.241   -0.072
    eTIV                    0.247    0.042    5.956    0.000    0.166    0.329
  vascular_risk_sumcorr ~~
    eTIV                    0.113    0.048    2.362    0.018    0.019    0.207

Intercepts:
                    est.std  Std.Err  z-value  P(>|z|) ci.lower ci.upper
   .iy               -0.131    0.043   -3.043    0.002   -0.215   -0.047
   .sy                1.113    0.109   10.170    0.000    0.899    1.328
   .y1                0.000                               0.000    0.000
   .y2                0.000                               0.000    0.000
   .y3                0.000                               0.000    0.000
   .y4                0.000                               0.000    0.000
   .y5                0.000                               0.000    0.000
   .ix               -0.020    0.049   -0.410    0.682   -0.117    0.077
   .sx                0.126    0.096    1.323    0.186   -0.061    0.314
   .x1                0.000                               0.000    0.000
   .x2                0.000                               0.000    0.000
   .x3                0.000                               0.000    0.000
   .x4                0.000                               0.000    0.000
   .x5                0.000                               0.000    0.000
    age_M00           0.000                               0.000    0.000
    edyears           0.000                               0.000    0.000
    sex               0.000                               0.000    0.000
    vsclr_rsk_smcr    0.000                               0.000    0.000
    eTIV              0.000                               0.000    0.000

Variances:
                    est.std  Std.Err  z-value  P(>|z|) ci.lower ci.upper
   .iy                0.811    0.032   25.314    0.000    0.748    0.874
   .sy                0.987    0.015   67.612    0.000    0.959    1.016
   .y1     (tht_y)    0.017    0.002    9.486    0.000    0.014    0.021
   .y2     (tht_y)    0.017    0.002    9.615    0.000    0.014    0.021
   .y3     (tht_y)    0.017    0.002    9.686    0.000    0.014    0.021
   .y4     (tht_y)    0.017    0.002    9.703    0.000    0.013    0.020
   .y5     (tht_y)    0.016    0.002    9.670    0.000    0.013    0.020
   .ix                0.916    0.027   34.169    0.000    0.863    0.968
   .sx                0.927    0.053   17.546    0.000    0.823    1.030
   .x1     (tht_x)    0.139    0.016    8.579    0.000    0.107    0.171
   .x2     (tht_x)    0.137    0.016    8.671    0.000    0.106    0.168
   .x3     (tht_x)    0.133    0.015    8.659    0.000    0.103    0.164
   .x4     (tht_x)    0.128    0.015    8.557    0.000    0.099    0.158
   .x5     (tht_x)    0.122    0.015    8.370    0.000    0.094    0.151
    ag_M00            1.000                               1.000    1.000
```

sex	1.000	1.000	1.000
edyers	1.000	1.000	1.000
vscl__	1.000	1.000	1.000
eTIV	1.000	1.000	1.000

BLGCM - Total WMH volumes vs temporal cortical thickness

Unstandardised solution

```
lavaan 0.6.17 ended normally after 112 iterations

  Estimator                                         ML
  Optimization method                           NLMINB
  Number of model parameters                        59
  Number of equality constraints                    12

  Number of observations                           451
  Number of missing patterns                        80

Model Test User Model:
                                              Standard      Scaled
  Test Statistic                               132.531     106.286
  Degrees of freedom                                88          88
  P-value (Chi-square)                           0.002       0.090
  Scaling correction factor                                  1.247
    Yuan-Bentler correction (Mplus variant)

Model Test Baseline Model:

  Test statistic                              7167.090    5222.802
  Degrees of freedom                               105         105
  P-value                                        0.000       0.000
  Scaling correction factor                                  1.372

User Model versus Baseline Model:

  Comparative Fit Index (CFI)                    0.994       0.996
  Tucker-Lewis Index (TLI)                       0.992       0.996

  Robust Comparative Fit Index (CFI)                         0.997
  Robust Tucker-Lewis Index (TLI)                            0.996

Loglikelihood and Information Criteria:

  Loglikelihood user model (H0)              -4612.824   -4612.824
  Scaling correction factor                                  1.035
      for the MLR correction
  Loglikelihood unrestricted model (H1)      -4546.558   -4546.558
  Scaling correction factor                                  1.265
```

```
      for the MLR correction

  Akaike (AIC)                                9319.648    9319.648
  Bayesian (BIC)                              9512.887    9512.887
  Sample-size adjusted Bayesian (SABIC)       9363.726    9363.726

Root Mean Square Error of Approximation:

  RMSEA                                          0.033       0.021
  90 Percent confidence interval - lower         0.021       0.000
  90 Percent confidence interval - upper         0.045       0.034
  P-value H_0: RMSEA <= 0.050                    0.993       1.000
  P-value H_0: RMSEA >= 0.080                    0.000       0.000

  Robust RMSEA                                               0.029
  90 Percent confidence interval - lower                     0.000
  90 Percent confidence interval - upper                     0.050
  P-value H_0: Robust RMSEA <= 0.050                         0.952
  P-value H_0: Robust RMSEA >= 0.080                         0.000

Standardized Root Mean Square Residual:

  SRMR                                           0.017       0.017

Parameter Estimates:

  Standard errors                             Sandwich
  Information bread                           Observed
  Observed information based on                Hessian

Latent Variables:
                   Estimate  Std.Err  z-value  P(>|z|)   Std.lv  Std.all
  iy =~
    y1                1.000                                0.998    0.991
    y2                1.000                                0.998    0.990
    y3                1.000                                0.998    0.984
    y4                1.000                                0.998    0.975
    y5                1.000                                0.998    0.963
  sy =~
    y1                0.000                                0.000    0.000
    y2                1.000                                0.063    0.063
    y3                2.000                                0.126    0.125
    y4                3.000                                0.190    0.185
    y5                4.000                                0.253    0.244
  ix =~
```

```
    x1                1.000                               0.953    0.947
    x2                1.000                               0.953    0.945
    x3                1.000                               0.953    0.935
    x4                1.000                               0.953    0.916
    x5                1.000                               0.953    0.892
  sx =~
    x1                0.000                               0.000    0.000
    x2                1.000                               0.098    0.097
    x3                2.000                               0.196    0.192
    x4                3.000                               0.295    0.283
    x5                4.000                               0.393    0.367

Regressions:
                   Estimate  Std.Err  z-value  P(>|z|)   Std.lv  Std.all
  iy ~
    age_M00           0.373    0.044    8.442    0.000    0.374    0.374
    sex               0.189    0.060    3.129    0.002    0.189    0.189
    edyears          -0.027    0.044   -0.618    0.537   -0.027   -0.027
    vsclr_rsk_smcr    0.101    0.045    2.271    0.023    0.102    0.102
    eTIV              0.259    0.056    4.638    0.000    0.260    0.260
  sy ~
    age_M00          -0.005    0.004   -1.102    0.270   -0.077   -0.077
    sex               0.000    0.006    0.080    0.936    0.007    0.007
    edyears          -0.004    0.004   -1.067    0.286   -0.071   -0.071
    vsclr_rsk_smcr   -0.001    0.004   -0.279    0.780   -0.019   -0.019
    eTIV             -0.002    0.005   -0.352    0.725   -0.027   -0.027
  ix ~
    age_M00          -0.326    0.046   -7.136    0.000   -0.342   -0.342
    sex              -0.074    0.060   -1.237    0.216   -0.078   -0.078
    edyears          -0.040    0.049   -0.819    0.413   -0.042   -0.042
    vsclr_rsk_smcr   -0.077    0.046   -1.659    0.097   -0.080   -0.080
    eTIV             -0.119    0.062   -1.920    0.055   -0.124   -0.124
  sx ~
    age_M00          -0.024    0.009   -2.789    0.005   -0.248   -0.248
    sex               0.012    0.011    1.112    0.266    0.125    0.125
    edyears           0.012    0.008    1.536    0.124    0.122    0.122
    vsclr_rsk_smcr    0.001    0.008    0.126    0.900    0.010    0.010
    eTIV              0.009    0.011    0.834    0.404    0.090    0.090

Covariances:
                           Estimate  Std.Err  z-value  P(>|z|)   Std.lv  Std.all
 .iy ~~
   .sy                        0.002    0.004    0.419    0.675    0.030    0.030
 .ix ~~
   .sx                       -0.011    0.010   -1.145    0.252   -0.134   -0.134
```

```
  .y1 ~~
    .x1      (tht_)           -0.002    0.001   -1.317    0.188   -0.002   -0.042
  .y2 ~~
    .x2      (tht_)           -0.002    0.001   -1.317    0.188   -0.002   -0.042
  .y3 ~~
    .x3      (tht_)           -0.002    0.001   -1.317    0.188   -0.002   -0.042
  .y4 ~~
    .x4      (tht_)           -0.002    0.001   -1.317    0.188   -0.002   -0.042
  .y5 ~~
    .x5      (tht_)           -0.002    0.001   -1.317    0.188   -0.002   -0.042
  .iy ~~
    .ix                       -0.090    0.041   -2.203    0.028   -0.113   -0.113
    .sx                       -0.015    0.007   -2.042    0.041   -0.180   -0.180
  .sy ~~
    .ix                       -0.008    0.004   -1.975    0.048   -0.135   -0.135
    .sx                       -0.000    0.001   -0.586    0.558   -0.083   -0.083
   age_M00 ~~
     sex                      -0.165    0.046   -3.607    0.000   -0.165   -0.165
     edyears                  -0.119    0.048   -2.478    0.013   -0.119   -0.119
     vsclr__                   0.157    0.047    3.335    0.001    0.157    0.157
     eTIV                      0.057    0.046    1.234    0.217    0.057    0.057
   sex ~~
     edyears                  -0.238    0.043   -5.569    0.000   -0.238   -0.238
     vsclr__                  -0.184    0.045   -4.128    0.000   -0.184   -0.184
     eTIV                     -0.668    0.021  -31.365    0.000   -0.668   -0.668
   edyears ~~
     vsclr__                  -0.156    0.043   -3.625    0.000   -0.156   -0.156
     eTIV                      0.247    0.042    5.956    0.000    0.247    0.247
   vascular_risk_sumcorr ~~
     eTIV                      0.113    0.048    2.362    0.018    0.113    0.113

Intercepts:
                   Estimate  Std.Err  z-value  P(>|z|)   Std.lv  Std.all
   .iy               -0.130    0.043   -3.063    0.002   -0.131   -0.131
   .sy                0.070    0.004   17.651    0.000    1.112    1.112
   .y1                0.000                                0.000    0.000
   .y2                0.000                                0.000    0.000
   .y3                0.000                                0.000    0.000
   .y4                0.000                                0.000    0.000
   .y5                0.000                                0.000    0.000
   .ix                0.056    0.045    1.254    0.210    0.059    0.059
   .sx               -0.028    0.008   -3.610    0.000   -0.283   -0.283
   .x1                0.000                                0.000    0.000
   .x2                0.000                                0.000    0.000
   .x3                0.000                                0.000    0.000
```

```
   .x4                 0.000                                       0.000    0.000
   .x5                 0.000                                       0.000    0.000
    age_M00            0.000                                       0.000    0.000
    edyears            0.000                                       0.000    0.000
    sex                0.000                                       0.000    0.000
    vsclr_rsk_smcr     0.000                                       0.000    0.000
    eTIV               0.000                                       0.000    0.000

Variances:
                    Estimate  Std.Err  z-value  P(>|z|)   Std.lv  Std.all
   .iy                 0.808    0.049   16.410    0.000    0.811    0.811
   .sy                 0.004    0.001    4.930    0.000    0.987    0.987
   .y1      (tht_y)    0.018    0.002   10.492    0.000    0.018    0.017
   .y2      (tht_y)    0.018    0.002   10.492    0.000    0.018    0.017
   .y3      (tht_y)    0.018    0.002   10.492    0.000    0.018    0.017
   .y4      (tht_y)    0.018    0.002   10.492    0.000    0.018    0.017
   .y5      (tht_y)    0.018    0.002   10.492    0.000    0.018    0.016
   .ix                 0.786    0.065   12.178    0.000    0.865    0.865
   .sx                 0.009    0.003    2.840    0.005    0.903    0.903
   .x1      (tht_x)    0.105    0.012    9.027    0.000    0.105    0.104
   .x2      (tht_x)    0.105    0.012    9.027    0.000    0.105    0.104
   .x3      (tht_x)    0.105    0.012    9.027    0.000    0.105    0.101
   .x4      (tht_x)    0.105    0.012    9.027    0.000    0.105    0.097
   .x5      (tht_x)    0.105    0.012    9.027    0.000    0.105    0.092
    ag_M00             1.000                                       1.000    1.000
    sex                1.000                                       1.000    1.000
    edyers             1.000                                       1.000    1.000
    vscl__             1.000                                       1.000    1.000
    eTIV               1.000                                       1.000    1.000

R-Square:
                    Estimate
    iy                 0.189
    sy                 0.013
    y1                 0.983
    y2                 0.983
    y3                 0.983
    y4                 0.983
    y5                 0.984
    ix                 0.135
    sx                 0.097
    x1                 0.896
    x2                 0.896
    x3                 0.899
    x4                 0.903
```

x5	0.908

Standardised solution

Latent Variables:

	est.std	Std.Err	z-value	P(>|z|)	ci.lower	ci.upper
iy =~						
y1	0.991	0.001	1076.052	0.000	0.990	0.993
y2	0.990	0.005	216.412	0.000	0.981	0.999
y3	0.984	0.008	115.816	0.000	0.968	1.001
y4	0.975	0.012	80.579	0.000	0.951	0.999
y5	0.963	0.015	62.625	0.000	0.932	0.993
sy =~						
y1	0.000				0.000	0.000
y2	0.063	0.007	9.593	0.000	0.050	0.075
y3	0.125	0.013	9.534	0.000	0.099	0.150
y4	0.185	0.019	9.536	0.000	0.147	0.223
y5	0.244	0.025	9.597	0.000	0.194	0.294
ix =~						
x1	0.947	0.007	139.117	0.000	0.933	0.960
x2	0.945	0.012	79.668	0.000	0.922	0.968
x3	0.935	0.018	51.166	0.000	0.899	0.971
x4	0.916	0.024	38.506	0.000	0.870	0.963
x5	0.892	0.029	31.249	0.000	0.836	0.947
sx =~						
x1	0.000				0.000	0.000
x2	0.097	0.016	5.985	0.000	0.065	0.129
x3	0.192	0.032	5.939	0.000	0.129	0.256
x4	0.283	0.047	5.990	0.000	0.190	0.376
x5	0.367	0.060	6.131	0.000	0.250	0.485

Regressions:

	est.std	Std.Err	z-value	P(>|z|)	ci.lower	ci.upper
iy ~						
age_M00	0.374	0.042	8.934	0.000	0.292	0.456
sex	0.189	0.060	3.161	0.002	0.072	0.306
edyears	-0.027	0.044	-0.619	0.536	-0.114	0.059
vsclr_rsk_smcr	0.102	0.044	2.303	0.021	0.015	0.188
eTIV	0.260	0.054	4.784	0.000	0.153	0.366
sy ~						
age_M00	-0.077	0.069	-1.112	0.266	-0.212	0.058
sex	0.007	0.088	0.080	0.937	-0.166	0.180
edyears	-0.071	0.067	-1.057	0.291	-0.202	0.060
vsclr_rsk_smcr	-0.019	0.066	-0.280	0.780	-0.148	0.111

```
    eTIV              -0.027    0.075   -0.356    0.722   -0.174    0.120
  ix ~
    age_M00           -0.342    0.046   -7.413    0.000   -0.432   -0.252
    sex               -0.078    0.063   -1.246    0.213   -0.201    0.045
    edyears           -0.042    0.051   -0.822    0.411   -0.142    0.058
    vsclr_rsk_smcr    -0.080    0.048   -1.676    0.094   -0.174    0.014
    eTIV              -0.124    0.064   -1.936    0.053   -0.250    0.002
  sx ~
    age_M00           -0.248    0.101   -2.452    0.014   -0.446   -0.050
    sex                0.125    0.106    1.181    0.237   -0.082    0.332
    edyears            0.122    0.074    1.660    0.097   -0.022    0.267
    vsclr_rsk_smcr     0.010    0.083    0.125    0.901   -0.153    0.173
    eTIV               0.090    0.105    0.862    0.388   -0.115    0.295

Covariances:
                           est.std  Std.Err  z-value  P(>|z|) ci.lower ci.upper
 .iy ~~
   .sy                       0.030    0.072    0.412    0.680   -0.111    0.170
 .ix ~~
   .sx                      -0.134    0.101   -1.328    0.184   -0.331    0.064
 .y1 ~~
   .x1      (tht_)          -0.042    0.031   -1.325    0.185   -0.103    0.020
 .y2 ~~
   .x2      (tht_)          -0.042    0.031   -1.325    0.185   -0.103    0.020
 .y3 ~~
   .x3      (tht_)          -0.042    0.031   -1.325    0.185   -0.103    0.020
 .y4 ~~
   .x4      (tht_)          -0.042    0.031   -1.325    0.185   -0.103    0.020
 .y5 ~~
   .x5      (tht_)          -0.042    0.031   -1.325    0.185   -0.103    0.020
 .iy ~~
   .ix                      -0.113    0.050   -2.248    0.025   -0.211   -0.014
   .sx                      -0.180    0.082   -2.197    0.028   -0.340   -0.019
 .sy ~~
   .ix                      -0.135    0.064   -2.122    0.034   -0.260   -0.010
   .sx                      -0.083    0.144   -0.572    0.567   -0.365    0.200
  age_M00 ~~
    sex                     -0.165    0.046   -3.607    0.000   -0.255   -0.075
    edyears                 -0.119    0.048   -2.478    0.013   -0.214   -0.025
    vsclr__                  0.157    0.047    3.335    0.001    0.065    0.249
    eTIV                     0.057    0.046    1.234    0.217   -0.033    0.146
  sex ~~
    edyears                 -0.238    0.043   -5.569    0.000   -0.321   -0.154
    vsclr__                 -0.184    0.045   -4.128    0.000   -0.272   -0.097
    eTIV                    -0.668    0.021  -31.365    0.000   -0.710   -0.626
```

```
  edyears ~~
    vsclr__              -0.156    0.043   -3.625    0.000   -0.241   -0.072
    eTIV                  0.247    0.042    5.956    0.000    0.166    0.329
  vascular_risk_sumcorr ~~
    eTIV                  0.113    0.048    2.362    0.018    0.019    0.207

Intercepts:
                    est.std  Std.Err  z-value  P(>|z|) ci.lower ci.upper
   .iy               -0.131    0.043   -3.043    0.002   -0.215   -0.047
   .sy                1.112    0.109   10.165    0.000    0.898    1.327
   .y1                0.000                               0.000    0.000
   .y2                0.000                               0.000    0.000
   .y3                0.000                               0.000    0.000
   .y4                0.000                               0.000    0.000
   .y5                0.000                               0.000    0.000
   .ix                0.059    0.047    1.260    0.208   -0.033    0.151
   .sx               -0.283    0.091   -3.108    0.002   -0.461   -0.104
   .x1                0.000                               0.000    0.000
   .x2                0.000                               0.000    0.000
   .x3                0.000                               0.000    0.000
   .x4                0.000                               0.000    0.000
   .x5                0.000                               0.000    0.000
    age_M00           0.000                               0.000    0.000
    edyears           0.000                               0.000    0.000
    sex               0.000                               0.000    0.000
    vsclr_rsk_smcr    0.000                               0.000    0.000
    eTIV              0.000                               0.000    0.000

Variances:
                    est.std  Std.Err  z-value  P(>|z|) ci.lower ci.upper
   .iy                0.811    0.032   25.310    0.000    0.748    0.874
   .sy                0.987    0.015   67.588    0.000    0.959    1.016
   .y1      (tht_y)   0.017    0.002    9.474    0.000    0.014    0.021
   .y2      (tht_y)   0.017    0.002    9.604    0.000    0.014    0.021
   .y3      (tht_y)   0.017    0.002    9.675    0.000    0.014    0.021
   .y4      (tht_y)   0.017    0.002    9.692    0.000    0.013    0.020
   .y5      (tht_y)   0.016    0.002    9.659    0.000    0.013    0.020
   .ix                0.865    0.032   26.909    0.000    0.802    0.928
   .sx                0.903    0.048   18.970    0.000    0.810    0.997
   .x1      (tht_x)   0.104    0.013    8.070    0.000    0.079    0.129
   .x2      (tht_x)   0.104    0.013    8.195    0.000    0.079    0.128
   .x3      (tht_x)   0.101    0.012    8.242    0.000    0.077    0.125
   .x4      (tht_x)   0.097    0.012    8.243    0.000    0.074    0.121
   .x5      (tht_x)   0.092    0.011    8.191    0.000    0.070    0.114
    ag_M00            1.000                               1.000    1.000
```

sex	1.000	1.000	1.000
edyers	1.000	1.000	1.000
vscl__	1.000	1.000	1.000
eTIV	1.000	1.000	1.000

BLGCM - Total WMH volumes vs cingulate cortical thickness

Unstandardised solution

```
lavaan 0.6.17 ended normally after 125 iterations

  Estimator                                         ML
  Optimization method                           NLMINB
  Number of model parameters                        59
  Number of equality constraints                    12

  Number of observations                           451
  Number of missing patterns                        79

Model Test User Model:
                                              Standard      Scaled
  Test Statistic                               115.287      98.629
  Degrees of freedom                                88          88
  P-value (Chi-square)                           0.027       0.206
  Scaling correction factor                                  1.169
    Yuan-Bentler correction (Mplus variant)

Model Test Baseline Model:

  Test statistic                              6982.899    5594.460
  Degrees of freedom                               105         105
  P-value                                        0.000       0.000
  Scaling correction factor                                  1.248

User Model versus Baseline Model:

  Comparative Fit Index (CFI)                    0.996       0.998
  Tucker-Lewis Index (TLI)                       0.995       0.998

  Robust Comparative Fit Index (CFI)                         0.996
  Robust Tucker-Lewis Index (TLI)                            0.995

Loglikelihood and Information Criteria:

  Loglikelihood user model (H0)              -4689.161   -4689.161
  Scaling correction factor                                  0.930
      for the MLR correction
  Loglikelihood unrestricted model (H1)      -4631.518   -4631.518
  Scaling correction factor                                  1.168
```

```
      for the MLR correction

  Akaike (AIC)                                9472.322    9472.322
  Bayesian (BIC)                              9665.561    9665.561
  Sample-size adjusted Bayesian (SABIC)       9516.401    9516.401

Root Mean Square Error of Approximation:

  RMSEA                                          0.026       0.016
  90 Percent confidence interval - lower         0.009       0.000
  90 Percent confidence interval - upper         0.039       0.030
  P-value H_0: RMSEA <= 0.050                    1.000       1.000
  P-value H_0: RMSEA >= 0.080                    0.000       0.000

  Robust RMSEA                                               0.030
  90 Percent confidence interval - lower                     0.000
  90 Percent confidence interval - upper                     0.049
  P-value H_0: Robust RMSEA <= 0.050                         0.956
  P-value H_0: Robust RMSEA >= 0.080                         0.000

Standardized Root Mean Square Residual:

  SRMR                                           0.015       0.015

Parameter Estimates:

  Standard errors                             Sandwich
  Information bread                           Observed
  Observed information based on                Hessian

Latent Variables:
                   Estimate  Std.Err  z-value  P(>|z|)   Std.lv  Std.all
  iy =~
    y1                1.000                               0.998    0.991
    y2                1.000                               0.998    0.990
    y3                1.000                               0.998    0.984
    y4                1.000                               0.998    0.975
    y5                1.000                               0.998    0.963
  sy =~
    y1                0.000                               0.000    0.000
    y2                1.000                               0.063    0.063
    y3                2.000                               0.127    0.125
    y4                3.000                               0.190    0.186
    y5                4.000                               0.253    0.244
  ix =~
```

```
    x1                1.000                               0.960    0.943
    x2                1.000                               0.960    0.950
    x3                1.000                               0.960    0.946
    x4                1.000                               0.960    0.929
    x5                1.000                               0.960    0.903
  sx =~
    x1                0.000                               0.000    0.000
    x2                1.000                               0.115    0.114
    x3                2.000                               0.230    0.226
    x4                3.000                               0.344    0.333
    x5                4.000                               0.459    0.432

Regressions:
                   Estimate  Std.Err  z-value  P(>|z|)   Std.lv  Std.all
  iy ~
    age_M00           0.373    0.044    8.443    0.000    0.374    0.374
    sex               0.188    0.060    3.125    0.002    0.188    0.188
    edyears          -0.027    0.044   -0.621    0.535   -0.027   -0.027
    vsclr_rsk_smcr    0.102    0.045    2.275    0.023    0.102    0.102
    eTIV              0.259    0.056    4.636    0.000    0.260    0.260
  sy ~
    age_M00          -0.005    0.004   -1.121    0.262   -0.078   -0.078
    sex               0.001    0.006    0.092    0.927    0.008    0.008
    edyears          -0.004    0.004   -1.048    0.294   -0.069   -0.069
    vsclr_rsk_smcr   -0.001    0.004   -0.328    0.743   -0.022   -0.022
    eTIV             -0.002    0.005   -0.343    0.731   -0.026   -0.026
  ix ~
    age_M00          -0.213    0.052   -4.136    0.000   -0.222   -0.222
    sex              -0.031    0.060   -0.520    0.603   -0.033   -0.033
    edyears          -0.057    0.050   -1.133    0.257   -0.059   -0.059
    vsclr_rsk_smcr   -0.098    0.046   -2.146    0.032   -0.102   -0.102
    eTIV             -0.091    0.066   -1.367    0.172   -0.094   -0.094
  sx ~
    age_M00          -0.027    0.009   -3.069    0.002   -0.235   -0.235
    sex               0.003    0.011    0.286    0.775    0.028    0.028
    edyears           0.018    0.009    2.122    0.034    0.157    0.157
    vsclr_rsk_smcr   -0.004    0.009   -0.434    0.664   -0.032   -0.032
    eTIV             -0.016    0.012   -1.320    0.187   -0.136   -0.136

Covariances:
                           Estimate  Std.Err  z-value  P(>|z|)   Std.lv  Std.all
 .iy ~~
   .sy                        0.002    0.004    0.407    0.684    0.029    0.029
 .ix ~~
   .sx                       -0.023    0.009   -2.441    0.015   -0.227   -0.227
```

```
  .y1 ~~
    .x1      (tht_)          -0.004    0.002   -2.334    0.020   -0.004   -0.082
  .y2 ~~
    .x2      (tht_)          -0.004    0.002   -2.334    0.020   -0.004   -0.082
  .y3 ~~
    .x3      (tht_)          -0.004    0.002   -2.334    0.020   -0.004   -0.082
  .y4 ~~
    .x4      (tht_)          -0.004    0.002   -2.334    0.020   -0.004   -0.082
  .y5 ~~
    .x5      (tht_)          -0.004    0.002   -2.334    0.020   -0.004   -0.082
  .iy ~~
    .ix                      -0.115    0.039   -2.938    0.003   -0.138   -0.138
    .sx                      -0.021    0.007   -2.924    0.003   -0.217   -0.217
  .sy ~~
    .ix                      -0.009    0.004   -2.143    0.032   -0.148   -0.148
    .sx                      -0.001    0.001   -0.712    0.476   -0.075   -0.075
   age_M00 ~~
     sex                     -0.165    0.046   -3.607    0.000   -0.165   -0.165
     edyears                 -0.119    0.048   -2.478    0.013   -0.119   -0.119
     vsclr__                  0.157    0.047    3.335    0.001    0.157    0.157
     eTIV                     0.057    0.046    1.234    0.217    0.057    0.057
   sex ~~
     edyears                 -0.238    0.043   -5.569    0.000   -0.238   -0.238
     vsclr__                 -0.184    0.045   -4.128    0.000   -0.184   -0.184
     eTIV                    -0.668    0.021  -31.365    0.000   -0.668   -0.668
   edyears ~~
     vsclr__                 -0.156    0.043   -3.625    0.000   -0.156   -0.156
     eTIV                     0.247    0.042    5.956    0.000    0.247    0.247
   vascular_risk_sumcorr ~~
     eTIV                     0.113    0.048    2.362    0.018    0.113    0.113

Intercepts:
                   Estimate  Std.Err  z-value  P(>|z|)   Std.lv  Std.all
    .iy              -0.130    0.043   -3.062    0.002   -0.131   -0.131
    .sy               0.070    0.004   17.686    0.000    1.108    1.108
    .y1               0.000                                0.000    0.000
    .y2               0.000                                0.000    0.000
    .y3               0.000                                0.000    0.000
    .y4               0.000                                0.000    0.000
    .y5               0.000                                0.000    0.000
    .ix               0.149    0.047    3.138    0.002    0.155    0.155
    .sx              -0.077    0.009   -9.032    0.000   -0.671   -0.671
    .x1               0.000                                0.000    0.000
    .x2               0.000                                0.000    0.000
    .x3               0.000                                0.000    0.000
```

```
   .x4                  0.000                                0.000    0.000
   .x5                  0.000                                0.000    0.000
    age_M00             0.000                                0.000    0.000
    edyears             0.000                                0.000    0.000
    sex                 0.000                                0.000    0.000
    vsclr_rsk_smcr      0.000                                0.000    0.000
    eTIV                0.000                                0.000    0.000

Variances:
                   Estimate  Std.Err  z-value  P(>|z|)   Std.lv  Std.all
   .iy                0.808    0.049   16.421    0.000    0.811    0.811
   .sy                0.004    0.001    4.908    0.000    0.987    0.987
   .y1      (tht_y)   0.018    0.002   10.504    0.000    0.018    0.017
   .y2      (tht_y)   0.018    0.002   10.504    0.000    0.018    0.017
   .y3      (tht_y)   0.018    0.002   10.504    0.000    0.018    0.017
   .y4      (tht_y)   0.018    0.002   10.504    0.000    0.018    0.017
   .y5      (tht_y)   0.018    0.002   10.504    0.000    0.018    0.016
   .ix                0.854    0.064   13.329    0.000    0.926    0.926
   .sx                0.012    0.002    5.351    0.000    0.887    0.887
   .x1      (tht_x)   0.116    0.010   11.307    0.000    0.116    0.111
   .x2      (tht_x)   0.116    0.010   11.307    0.000    0.116    0.113
   .x3      (tht_x)   0.116    0.010   11.307    0.000    0.116    0.112
   .x4      (tht_x)   0.116    0.010   11.307    0.000    0.116    0.108
   .x5      (tht_x)   0.116    0.010   11.307    0.000    0.116    0.102
    ag_M00            1.000                               1.000    1.000
    sex               1.000                               1.000    1.000
    edyers            1.000                               1.000    1.000
    vscl__            1.000                               1.000    1.000
    eTIV              1.000                               1.000    1.000

R-Square:
                   Estimate
    iy                0.189
    sy                0.013
    y1                0.983
    y2                0.983
    y3                0.983
    y4                0.983
    y5                0.984
    ix                0.074
    sx                0.113
    x1                0.889
    x2                0.887
    x3                0.888
    x4                0.892
```

```
    x5                 0.898
```

Standardised solution

```
Latent Variables:
                    est.std  Std.Err  z-value  P(>|z|) ci.lower ci.upper
  iy =~
    y1                0.991    0.001 1077.463    0.000    0.990    0.993
    y2                0.990    0.005  216.157    0.000    0.981    0.999
    y3                0.984    0.009  115.663    0.000    0.968    1.001
    y4                0.975    0.012   80.456    0.000    0.952    0.999
    y5                0.963    0.015   62.509    0.000    0.933    0.993
  sy =~
    y1                0.000                               0.000    0.000
    y2                0.063    0.007    9.553    0.000    0.050    0.076
    y3                0.125    0.013    9.495    0.000    0.099    0.151
    y4                0.186    0.020    9.499    0.000    0.147    0.224
    y5                0.244    0.026    9.561    0.000    0.194    0.295
  ix =~
    x1                0.943    0.006  157.804    0.000    0.931    0.954
    x2                0.950    0.010   93.941    0.000    0.930    0.970
    x3                0.946    0.017   55.373    0.000    0.912    0.979
    x4                0.929    0.024   39.279    0.000    0.883    0.976
    x5                0.903    0.029   30.877    0.000    0.846    0.961
  sx =~
    x1                0.000                               0.000    0.000
    x2                0.114    0.011   10.592    0.000    0.093    0.135
    x3                0.226    0.021   10.536    0.000    0.184    0.268
    x4                0.333    0.031   10.667    0.000    0.272    0.395
    x5                0.432    0.039   10.976    0.000    0.355    0.509

Regressions:
                    est.std  Std.Err  z-value  P(>|z|) ci.lower ci.upper
  iy ~
    age_M00           0.374    0.042    8.939    0.000    0.292    0.456
    sex               0.188    0.060    3.157    0.002    0.071    0.305
    edyears          -0.027    0.044   -0.622    0.534   -0.114    0.059
    vsclr_rsk_smcr    0.102    0.044    2.307    0.021    0.015    0.188
    eTIV              0.260    0.054    4.781    0.000    0.153    0.366
  sy ~
    age_M00          -0.078    0.069   -1.131    0.258   -0.213    0.057
    sex               0.008    0.088    0.092    0.927   -0.165    0.181
    edyears          -0.069    0.067   -1.039    0.299   -0.201    0.062
    vsclr_rsk_smcr   -0.022    0.066   -0.329    0.742   -0.152    0.108
```

```
    eTIV              -0.026    0.075   -0.347    0.729   -0.173    0.121
  ix ~
    age_M00           -0.222    0.052   -4.290    0.000   -0.324   -0.121
    sex               -0.033    0.062   -0.521    0.602   -0.155    0.090
    edyears           -0.059    0.052   -1.136    0.256   -0.162    0.043
    vsclr_rsk_smcr    -0.102    0.047   -2.162    0.031   -0.195   -0.010
    eTIV              -0.094    0.069   -1.370    0.171   -0.229    0.041
  sx ~
    age_M00           -0.235    0.075   -3.120    0.002   -0.383   -0.088
    sex                0.028    0.098    0.288    0.774   -0.164    0.221
    edyears            0.157    0.073    2.162    0.031    0.015    0.300
    vsclr_rsk_smcr    -0.032    0.075   -0.432    0.666   -0.179    0.114
    eTIV              -0.136    0.102   -1.331    0.183   -0.335    0.064

Covariances:
                           est.std  Std.Err  z-value  P(>|z|) ci.lower ci.upper
 .iy ~~
   .sy                       0.029    0.072    0.401    0.689   -0.112    0.169
 .ix ~~
   .sx                      -0.227    0.081   -2.808    0.005   -0.386   -0.069
 .y1 ~~
   .x1      (tht_)          -0.082    0.035   -2.337    0.019   -0.150   -0.013
 .y2 ~~
   .x2      (tht_)          -0.082    0.035   -2.337    0.019   -0.150   -0.013
 .y3 ~~
   .x3      (tht_)          -0.082    0.035   -2.337    0.019   -0.150   -0.013
 .y4 ~~
   .x4      (tht_)          -0.082    0.035   -2.337    0.019   -0.150   -0.013
 .y5 ~~
   .x5      (tht_)          -0.082    0.035   -2.337    0.019   -0.150   -0.013
 .iy ~~
   .ix                      -0.138    0.046   -3.022    0.003   -0.227   -0.048
   .sx                      -0.217    0.074   -2.953    0.003   -0.362   -0.073
 .sy ~~
   .ix                      -0.148    0.063   -2.366    0.018   -0.271   -0.025
   .sx                      -0.075    0.107   -0.702    0.483   -0.284    0.134
  age_M00 ~~
    sex                     -0.165    0.046   -3.607    0.000   -0.255   -0.075
    edyears                 -0.119    0.048   -2.478    0.013   -0.214   -0.025
    vsclr__                  0.157    0.047    3.335    0.001    0.065    0.249
    eTIV                     0.057    0.046    1.234    0.217   -0.033    0.146
  sex ~~
    edyears                 -0.238    0.043   -5.569    0.000   -0.321   -0.154
    vsclr__                 -0.184    0.045   -4.128    0.000   -0.272   -0.097
    eTIV                    -0.668    0.021  -31.365    0.000   -0.710   -0.626
```

```
  edyears ~~
    vsclr__                -0.156    0.043   -3.625    0.000   -0.241   -0.072
    eTIV                    0.247    0.042    5.956    0.000    0.166    0.329
  vascular_risk_sumcorr ~~
    eTIV                    0.113    0.048    2.362    0.018    0.019    0.207

Intercepts:
                   est.std  Std.Err  z-value  P(>|z|) ci.lower ci.upper
   .iy              -0.131    0.043   -3.042    0.002   -0.215   -0.046
   .sy               1.108    0.109   10.123    0.000    0.894    1.323
   .y1               0.000                               0.000    0.000
   .y2               0.000                               0.000    0.000
   .y3               0.000                               0.000    0.000
   .y4               0.000                               0.000    0.000
   .y5               0.000                               0.000    0.000
   .ix               0.155    0.049    3.135    0.002    0.058    0.252
   .sx              -0.671    0.092   -7.296    0.000   -0.852   -0.491
   .x1               0.000                               0.000    0.000
   .x2               0.000                               0.000    0.000
   .x3               0.000                               0.000    0.000
   .x4               0.000                               0.000    0.000
   .x5               0.000                               0.000    0.000
    age_M00          0.000                               0.000    0.000
    edyears          0.000                               0.000    0.000
    sex              0.000                               0.000    0.000
    vsclr_rsk_smcr   0.000                               0.000    0.000
    eTIV             0.000                               0.000    0.000

Variances:
                   est.std  Std.Err  z-value  P(>|z|) ci.lower ci.upper
   .iy               0.811    0.032   25.304    0.000    0.748    0.873
   .sy               0.987    0.015   67.176    0.000    0.958    1.016
   .y1     (tht_y)   0.017    0.002    9.482    0.000    0.014    0.021
   .y2     (tht_y)   0.017    0.002    9.613    0.000    0.014    0.021
   .y3     (tht_y)   0.017    0.002    9.686    0.000    0.014    0.021
   .y4     (tht_y)   0.017    0.002    9.704    0.000    0.013    0.020
   .y5     (tht_y)   0.016    0.002    9.671    0.000    0.013    0.020
   .ix               0.926    0.026   36.003    0.000    0.875    0.976
   .sx               0.887    0.044   20.256    0.000    0.801    0.973
   .x1     (tht_x)   0.111    0.011    9.900    0.000    0.089    0.134
   .x2     (tht_x)   0.113    0.012    9.724    0.000    0.090    0.136
   .x3     (tht_x)   0.112    0.012    9.406    0.000    0.089    0.136
   .x4     (tht_x)   0.108    0.012    9.025    0.000    0.085    0.132
   .x5     (tht_x)   0.102    0.012    8.634    0.000    0.079    0.126
    ag_M00           1.000                               1.000    1.000
```

sex	1.000	1.000	1.000
edyers	1.000	1.000	1.000
vscl__	1.000	1.000	1.000
eTIV	1.000	1.000	1.000

BLGCM - Total WMH volumes vs insular cortical thickness

Unstandardised solution

```
lavaan 0.6.17 ended normally after 108 iterations

  Estimator                                         ML
  Optimization method                           NLMINB
  Number of model parameters                        59
  Number of equality constraints                    12

  Number of observations                           451
  Number of missing patterns                        82

Model Test User Model:
                                              Standard      Scaled
  Test Statistic                                89.206      71.460
  Degrees of freedom                                88          88
  P-value (Chi-square)                           0.444       0.900
  Scaling correction factor                                  1.248
    Yuan-Bentler correction (Mplus variant)

Model Test Baseline Model:

  Test statistic                              6797.905    5046.912
  Degrees of freedom                               105         105
  P-value                                        0.000       0.000
  Scaling correction factor                                  1.347

User Model versus Baseline Model:

  Comparative Fit Index (CFI)                    1.000       1.000
  Tucker-Lewis Index (TLI)                       1.000       1.004

  Robust Comparative Fit Index (CFI)                         1.000
  Robust Tucker-Lewis Index (TLI)                            1.006

Loglikelihood and Information Criteria:

  Loglikelihood user model (H0)              -4777.608   -4777.608
  Scaling correction factor                                  0.989
      for the MLR correction
  Loglikelihood unrestricted model (H1)      -4733.005   -4733.005
  Scaling correction factor                                  1.246
```

```
    for the MLR correction

  Akaike (AIC)                                9649.216    9649.216
  Bayesian (BIC)                              9842.455    9842.455
  Sample-size adjusted Bayesian (SABIC)       9693.294    9693.294

Root Mean Square Error of Approximation:

  RMSEA                                          0.006       0.000
  90 Percent confidence interval - lower         0.000       0.000
  90 Percent confidence interval - upper         0.026       0.008
  P-value H_0: RMSEA <= 0.050                    1.000       1.000
  P-value H_0: RMSEA >= 0.080                    0.000       0.000

  Robust RMSEA                                               0.000
  90 Percent confidence interval - lower                     0.000
  90 Percent confidence interval - upper                     0.000
  P-value H_0: Robust RMSEA <= 0.050                         1.000
  P-value H_0: Robust RMSEA >= 0.080                         0.000

Standardized Root Mean Square Residual:

  SRMR                                           0.014       0.014

Parameter Estimates:

  Standard errors                             Sandwich
  Information bread                           Observed
  Observed information based on                Hessian

Latent Variables:
                   Estimate  Std.Err  z-value  P(>|z|)   Std.lv  Std.all
  iy =~
    y1                1.000                               0.998    0.991
    y2                1.000                               0.998    0.990
    y3                1.000                               0.998    0.984
    y4                1.000                               0.998    0.975
    y5                1.000                               0.998    0.962
  sy =~
    y1                0.000                               0.000    0.000
    y2                1.000                               0.063    0.063
    y3                2.000                               0.126    0.125
    y4                3.000                               0.190    0.185
    y5                4.000                               0.253    0.244
  ix =~
```

```
    x1                1.000                               0.940    0.929
    x2                1.000                               0.940    0.931
    x3                1.000                               0.940    0.923
    x4                1.000                               0.940    0.906
    x5                1.000                               0.940    0.882
  sx =~
    x1                0.000                               0.000    0.000
    x2                1.000                               0.101    0.100
    x3                2.000                               0.203    0.199
    x4                3.000                               0.304    0.293
    x5                4.000                               0.406    0.381

Regressions:
                   Estimate  Std.Err  z-value  P(>|z|)   Std.lv  Std.all
  iy ~
    age_M00           0.373    0.044    8.439    0.000    0.374    0.374
    sex               0.188    0.060    3.128    0.002    0.189    0.189
    edyears          -0.027    0.044   -0.618    0.536   -0.027   -0.027
    vsclr_rsk_smcr    0.101    0.045    2.271    0.023    0.102    0.102
    eTIV              0.259    0.056    4.633    0.000    0.260    0.260
  sy ~
    age_M00          -0.005    0.004   -1.064    0.287   -0.074   -0.074
    sex               0.000    0.006    0.074    0.941    0.007    0.007
    edyears          -0.004    0.004   -1.064    0.287   -0.070   -0.070
    vsclr_rsk_smcr   -0.001    0.004   -0.300    0.764   -0.020   -0.020
    eTIV             -0.001    0.005   -0.310    0.756   -0.024   -0.024
  ix ~
    age_M00          -0.194    0.050   -3.902    0.000   -0.206   -0.206
    sex              -0.083    0.064   -1.292    0.196   -0.088   -0.088
    edyears          -0.026    0.050   -0.532    0.595   -0.028   -0.028
    vsclr_rsk_smcr   -0.091    0.052   -1.738    0.082   -0.096   -0.096
    eTIV             -0.078    0.069   -1.134    0.257   -0.083   -0.083
  sx ~
    age_M00          -0.016    0.009   -1.787    0.074   -0.161   -0.161
    sex              -0.001    0.012   -0.056    0.955   -0.007   -0.007
    edyears           0.003    0.009    0.351    0.725    0.030    0.030
    vsclr_rsk_smcr    0.000    0.008    0.048    0.962    0.004    0.004
    eTIV             -0.010    0.011   -0.900    0.368   -0.099   -0.099

Covariances:
                           Estimate  Std.Err  z-value  P(>|z|)   Std.lv  Std.all
 .iy ~~
   .sy                        0.002    0.004    0.417    0.677    0.029    0.029
 .ix ~~
   .sx                       -0.010    0.010   -0.997    0.319   -0.112   -0.112
```

```
 .y1 ~~
   .x1      (tht_)         -0.001    0.002   -0.587    0.557   -0.001   -0.023
 .y2 ~~
   .x2      (tht_)         -0.001    0.002   -0.587    0.557   -0.001   -0.023
 .y3 ~~
   .x3      (tht_)         -0.001    0.002   -0.587    0.557   -0.001   -0.023
 .y4 ~~
   .x4      (tht_)         -0.001    0.002   -0.587    0.557   -0.001   -0.023
 .y5 ~~
   .x5      (tht_)         -0.001    0.002   -0.587    0.557   -0.001   -0.023
 .iy ~~
   .ix                     -0.165    0.042   -3.884    0.000   -0.201   -0.201
   .sx                     -0.025    0.009   -2.774    0.006   -0.280   -0.280
 .sy ~~
   .ix                     -0.009    0.004   -2.147    0.032   -0.154   -0.154
   .sx                     -0.002    0.001   -1.846    0.065   -0.274   -0.274
  age_M00 ~~
    sex                    -0.165    0.046   -3.607    0.000   -0.165   -0.165
    edyears                -0.119    0.048   -2.478    0.013   -0.119   -0.119
    vsclr__                 0.157    0.047    3.335    0.001    0.157    0.157
    eTIV                    0.057    0.046    1.234    0.217    0.057    0.057
  sex ~~
    edyears                -0.238    0.043   -5.569    0.000   -0.238   -0.238
    vsclr__                -0.184    0.045   -4.128    0.000   -0.184   -0.184
    eTIV                   -0.668    0.021  -31.365    0.000   -0.668   -0.668
  edyears ~~
    vsclr__                -0.156    0.043   -3.625    0.000   -0.156   -0.156
    eTIV                    0.247    0.042    5.956    0.000    0.247    0.247
  vascular_risk_sumcorr ~~
    eTIV                    0.113    0.048    2.362    0.018    0.113    0.113

Intercepts:
                   Estimate  Std.Err  z-value  P(>|z|)   Std.lv  Std.all
   .iy               -0.131    0.043   -3.066    0.002   -0.131   -0.131
   .sy                0.071    0.004   17.661    0.000    1.116    1.116
   .y1                0.000                                0.000    0.000
   .y2                0.000                                0.000    0.000
   .y3                0.000                                0.000    0.000
   .y4                0.000                                0.000    0.000
   .y5                0.000                                0.000    0.000
   .ix                0.023    0.047    0.491    0.624    0.025    0.025
   .sx               -0.012    0.009   -1.382    0.167   -0.118   -0.118
   .x1                0.000                                0.000    0.000
   .x2                0.000                                0.000    0.000
   .x3                0.000                                0.000    0.000
```

```
   .x4                0.000                                    0.000    0.000
   .x5                0.000                                    0.000    0.000
    age_M00           0.000                                    0.000    0.000
    edyears           0.000                                    0.000    0.000
    sex               0.000                                    0.000    0.000
    vsclr_rsk_smcr    0.000                                    0.000    0.000
    eTIV              0.000                                    0.000    0.000

Variances:
                   Estimate  Std.Err  z-value  P(>|z|)   Std.lv  Std.all
   .iy                0.808    0.049   16.419    0.000    0.811    0.811
   .sy                0.004    0.001    4.932    0.000    0.988    0.988
   .y1      (tht_y)   0.018    0.002   10.522    0.000    0.018    0.017
   .y2      (tht_y)   0.018    0.002   10.522    0.000    0.018    0.017
   .y3      (tht_y)   0.018    0.002   10.522    0.000    0.018    0.017
   .y4      (tht_y)   0.018    0.002   10.522    0.000    0.018    0.017
   .y5      (tht_y)   0.018    0.002   10.522    0.000    0.018    0.016
   .ix                0.835    0.063   13.187    0.000    0.944    0.944
   .sx                0.010    0.004    2.534    0.011    0.963    0.963
   .x1      (tht_x)   0.139    0.012   11.707    0.000    0.139    0.136
   .x2      (tht_x)   0.139    0.012   11.707    0.000    0.139    0.136
   .x3      (tht_x)   0.139    0.012   11.707    0.000    0.139    0.134
   .x4      (tht_x)   0.139    0.012   11.707    0.000    0.139    0.129
   .x5      (tht_x)   0.139    0.012   11.707    0.000    0.139    0.123
    ag_M00            1.000                                    1.000    1.000
    sex               1.000                                    1.000    1.000
    edyers            1.000                                    1.000    1.000
    vscl__            1.000                                    1.000    1.000
    eTIV              1.000                                    1.000    1.000

R-Square:
                   Estimate
    iy                0.189
    sy                0.012
    y1                0.983
    y2                0.983
    y3                0.983
    y4                0.983
    y5                0.984
    ix                0.056
    sx                0.037
    x1                0.864
    x2                0.864
    x3                0.866
    x4                0.871
```

```
    x5                0.877
```

Standardised solution

```
Latent Variables:
                   est.std  Std.Err  z-value  P(>|z|) ci.lower ci.upper
  iy =~
    y1               0.991    0.001 1078.261    0.000    0.990    0.993
    y2               0.990    0.005  215.888    0.000    0.981    0.999
    y3               0.984    0.009  115.467    0.000    0.967    1.001
    y4               0.975    0.012   80.299    0.000    0.951    0.999
    y5               0.962    0.015   62.382    0.000    0.932    0.993
  sy =~
    y1               0.000                               0.000    0.000
    y2               0.063    0.007    9.596    0.000    0.050    0.075
    y3               0.125    0.013    9.538    0.000    0.099    0.150
    y4               0.185    0.019    9.541    0.000    0.147    0.223
    y5               0.244    0.025    9.603    0.000    0.194    0.294
  ix =~
    x1               0.929    0.007  126.803    0.000    0.915    0.944
    x2               0.931    0.012   80.410    0.000    0.908    0.953
    x3               0.923    0.017   53.517    0.000    0.889    0.956
    x4               0.906    0.023   40.000    0.000    0.862    0.950
    x5               0.882    0.028   31.332    0.000    0.827    0.937
  sx =~
    x1               0.000                               0.000    0.000
    x2               0.100    0.020    5.087    0.000    0.062    0.139
    x3               0.199    0.039    5.090    0.000    0.122    0.276
    x4               0.293    0.057    5.183    0.000    0.182    0.404
    x5               0.381    0.071    5.364    0.000    0.242    0.520

Regressions:
                   est.std  Std.Err  z-value  P(>|z|) ci.lower ci.upper
  iy ~
    age_M00          0.374    0.042    8.930    0.000    0.292    0.456
    sex              0.189    0.060    3.160    0.002    0.072    0.306
    edyears         -0.027    0.044   -0.619    0.536   -0.114    0.059
    vsclr_rsk_smcr   0.102    0.044    2.303    0.021    0.015    0.188
    eTIV             0.260    0.054    4.779    0.000    0.153    0.366
  sy ~
    age_M00         -0.074    0.069   -1.073    0.283   -0.210    0.061
    sex              0.007    0.088    0.074    0.941   -0.167    0.180
    edyears         -0.070    0.067   -1.054    0.292   -0.201    0.060
    vsclr_rsk_smcr  -0.020    0.066   -0.301    0.764   -0.150    0.110
```

```
    eTIV              -0.024    0.075   -0.313    0.754   -0.171    0.124
  ix ~
    age_M00           -0.206    0.052   -3.992    0.000   -0.308   -0.105
    sex               -0.088    0.068   -1.294    0.196   -0.221    0.045
    edyears           -0.028    0.053   -0.532    0.595   -0.131    0.075
    vsclr_rsk_smcr    -0.096    0.055   -1.746    0.081   -0.205    0.012
    eTIV              -0.083    0.073   -1.135    0.256   -0.226    0.060
  sx ~
    age_M00           -0.161    0.087   -1.844    0.065   -0.333    0.010
    sex               -0.007    0.115   -0.056    0.955   -0.233    0.220
    edyears            0.030    0.085    0.358    0.721   -0.137    0.198
    vsclr_rsk_smcr     0.004    0.081    0.048    0.962   -0.156    0.164
    eTIV              -0.099    0.110   -0.905    0.365   -0.314    0.116

Covariances:
                          est.std  Std.Err  z-value  P(>|z|) ci.lower ci.upper
 .iy ~~
   .sy                      0.029    0.072    0.410    0.682   -0.111    0.170
 .ix ~~
   .sx                     -0.112    0.098   -1.140    0.254   -0.303    0.080
 .y1 ~~
   .x1      (tht_)         -0.023    0.039   -0.585    0.559   -0.099    0.054
 .y2 ~~
   .x2      (tht_)         -0.023    0.039   -0.585    0.559   -0.099    0.054
 .y3 ~~
   .x3      (tht_)         -0.023    0.039   -0.585    0.559   -0.099    0.054
 .y4 ~~
   .x4      (tht_)         -0.023    0.039   -0.585    0.559   -0.099    0.054
 .y5 ~~
   .x5      (tht_)         -0.023    0.039   -0.585    0.559   -0.099    0.054
 .iy ~~
   .ix                     -0.201    0.049   -4.088    0.000   -0.297   -0.105
   .sx                     -0.280    0.101   -2.773    0.006   -0.478   -0.082
 .sy ~~
   .ix                     -0.154    0.070   -2.202    0.028   -0.292   -0.017
   .sx                     -0.274    0.131   -2.097    0.036   -0.531   -0.018
  age_M00 ~~
    sex                    -0.165    0.046   -3.607    0.000   -0.255   -0.075
    edyears                -0.119    0.048   -2.478    0.013   -0.214   -0.025
    vsclr__                 0.157    0.047    3.335    0.001    0.065    0.249
    eTIV                    0.057    0.046    1.234    0.217   -0.033    0.146
  sex ~~
    edyears                -0.238    0.043   -5.569    0.000   -0.321   -0.154
    vsclr__                -0.184    0.045   -4.128    0.000   -0.272   -0.097
    eTIV                   -0.668    0.021  -31.365    0.000   -0.710   -0.626
```

```
  edyears ~~
    vsclr__                 -0.156    0.043   -3.625    0.000   -0.241   -0.072
    eTIV                     0.247    0.042    5.956    0.000    0.166    0.329
  vascular_risk_sumcorr ~~
    eTIV                     0.113    0.048    2.362    0.018    0.019    0.207

Intercepts:
                    est.std  Std.Err  z-value  P(>|z|) ci.lower ci.upper
   .iy               -0.131    0.043   -3.045    0.002   -0.215   -0.047
   .sy                1.116    0.110   10.169    0.000    0.901    1.331
   .y1                0.000                                0.000    0.000
   .y2                0.000                                0.000    0.000
   .y3                0.000                                0.000    0.000
   .y4                0.000                                0.000    0.000
   .y5                0.000                                0.000    0.000
   .ix                0.025    0.050    0.490    0.624   -0.074    0.123
   .sx               -0.118    0.089   -1.316    0.188   -0.293    0.058
   .x1                0.000                                0.000    0.000
   .x2                0.000                                0.000    0.000
   .x3                0.000                                0.000    0.000
   .x4                0.000                                0.000    0.000
   .x5                0.000                                0.000    0.000
    age_M00           0.000                                0.000    0.000
    edyears           0.000                                0.000    0.000
    sex               0.000                                0.000    0.000
    vsclr_rsk_smcr    0.000                                0.000    0.000
    eTIV              0.000                                0.000    0.000

Variances:
                    est.std  Std.Err  z-value  P(>|z|) ci.lower ci.upper
   .iy                0.811    0.032   25.326    0.000    0.748    0.874
   .sy                0.988    0.014   69.848    0.000    0.960    1.016
   .y1     (tht_y)    0.017    0.002    9.495    0.000    0.014    0.021
   .y2     (tht_y)    0.017    0.002    9.626    0.000    0.014    0.021
   .y3     (tht_y)    0.017    0.002    9.699    0.000    0.014    0.021
   .y4     (tht_y)    0.017    0.002    9.717    0.000    0.013    0.020
   .y5     (tht_y)    0.016    0.002    9.685    0.000    0.013    0.020
   .ix                0.944    0.023   41.435    0.000    0.899    0.989
   .sx                0.963    0.033   29.554    0.000    0.899    1.027
   .x1     (tht_x)    0.136    0.014    9.986    0.000    0.109    0.163
   .x2     (tht_x)    0.136    0.014   10.080    0.000    0.110    0.163
   .x3     (tht_x)    0.134    0.013    9.993    0.000    0.108    0.160
   .x4     (tht_x)    0.129    0.013    9.758    0.000    0.103    0.155
   .x5     (tht_x)    0.123    0.013    9.331    0.000    0.097    0.148
    ag_M00            1.000                                1.000    1.000
```

sex	1.000	1.000	1.000
edyers	1.000	1.000	1.000
vscl__	1.000	1.000	1.000
eTIV	1.000	1.000	1.000
